# Differential dysregulation of granule subsets in WASH-deficient neutrophil leukocytes resulting in inflammation

**DOI:** 10.1038/s41467-022-33230-y

**Published:** 2022-09-21

**Authors:** Jennifer L. Johnson, Elsa Meneses-Salas, Mahalakshmi Ramadass, Jlenia Monfregola, Farhana Rahman, Raquel Carvalho Gontijo, William B. Kiosses, Kersi Pestonjamasp, Dale Allen, Jinzhong Zhang, Douglas G. Osborne, Yanfang Peipei Zhu, Nathan Wineinger, Kasra Askari, Danni Chen, Juan Yu, Scott C. Henderson, Catherine C. Hedrick, Matilde Valeria Ursini, Sergio Grinstein, Daniel D. Billadeau, Sergio D. Catz

**Affiliations:** 1 Department of Molecular Medicine, The Scripps Research Institute, La Jolla, CA USA; 2 Division of Inflammation Biology, La Jolla Institute for Immunology, La Jolla, CA USA; 3 The Division of Oncology Research, Schulze Center for Novel Therapeutics, Mayo Clinic, Rochester, MN USA; 4 Research Translational Institute, Statistics, The Scripps Research Institute, La Jolla, CA USA; 5 Institute of Genetics and Biophysics A. Buzzati Traverso CNR, 80131 Naples, Italy; 6 Department of Biochemistry, University of Toronto, Toronto, ON Canada; 7 Present Address: Telethon Institute of Genetics and Medicine (TIGEM), Via Campi Flegrei 34, 80078 Pozzuoli, Naples Italy

**Keywords:** Inflammation, Membrane trafficking, Innate immunity, Neutrophils

## Abstract

Dysregulated secretion in neutrophil leukocytes associates with human inflammatory disease. The exocytosis response to triggering stimuli is sequential; gelatinase granules modulate the initiation of the innate immune response, followed by the release of pro-inflammatory azurophilic granules, requiring stronger stimulation. Exocytosis requires actin depolymerization which is actively counteracted under non-stimulatory conditions. Here we show that the actin nucleator, WASH, is necessary to maintain azurophilic granules in their refractory state by granule actin entrapment and interference with the Rab27a-JFC1 exocytic machinery. On the contrary, gelatinase granules of WASH-deficient neutrophil leukocytes are characterized by decreased Rac1, shortened granule-associated actin comets and impaired exocytosis. Rac1 activation restores exocytosis of these granules. In vivo, WASH deficiency induces exacerbated azurophilic granule exocytosis, inflammation, and decreased survival. WASH deficiency thus differentially impacts neutrophil granule subtypes, impairing exocytosis of granules that mediate the initiation of the neutrophil innate response while exacerbating pro-inflammatory granule secretion.

## Abstract (web summary)

Responsive exocytosis in neutrophil leukocytes involves actin depolymerisation-dependent sequential release of gelatinase granules, then strongly pro-inflammatory azurophilic granules. Here authors show that the actin nucleator protein WASH facilitates the initial step of innate immune activation by gelatinase granules while inhibiting release of pro-inflammatory granules.

## Introduction

Exocytosis is a crucial event in inflammation and host defense. Neutrophils contain several subsets of secretory organelles that hold a variety of highly toxic readily-releasable proteins and peptides and act as signaling hubs, so neutrophil granules are important for host defense against infections^1^. Neutrophil exocytosis is sequential. While highly responsive granules secrete cargoes that modulate the initiation of the innate immune response, the release of protease-loaded granules involved in pathogen killing requires priming and stronger stimulation, a process hereafter referred to as sequential exocytosis. Dysregulated secretion of this granule subtype results in indiscriminate protease and cell-permeabilizing peptide secretion. Therefore, dysregulation of neutrophil secretion mediates systemic inflammation, exacerbates the damage to the endothelium associated with endotoxemia and sepsis, and contributes to the development of auto-inflammatory and autoimmune diseases^2–7^. Both secretion and signaling are regulated by vesicular trafficking, but the mechanisms regulating the sequential, differential, exocytosis of neutrophil secretory organelles remain unknown.

Neutrophil secretory organelles are characterized by different propensities to undergo exocytosis, a mechanism thought to be regulated by different trafficking effectors^1^. Gelatinase and specific granules constitute a gradient of organelles with overlapping functions; they secrete MMP-9 (matrix-metalloprotein 9), which contributes to neutrophil migration, and regulate the production of reactive oxygen species (ROS) by the upregulation of the membrane-associated subunit of the NADPH oxidase at the plasma and phagosomal membranes. Azurophilic granules contain the most toxic cargoes including myeloperoxidase (MPO) and the serine proteases elastase, cathepsin G and proteinase 3, and require the strongest stimulation to undergo exocytosis, a process that if dysregulated, induces systemic inflammation^8^. For example, MPO, an abundant microbicidal protein of azurophilic granules, mediates endothelial damage^2,6,9^, is present in atherosclerotic lesions^10^ and is involved in the pathogenesis of coronary artery disease (CAD), sepsis and SIRS^10–13^. A positive correlation between plasma levels of neutrophil proteins and CAD in humans is well established^11,14^. In mice, MPO inhibition alters the inflammatory tone of atherosclerotic lesions and helps prevent atherosclerotic plaque rupture^15^ while neutrophil permanence in circulation aggravates myocardial infarction, and temporary depletion reduces atherogenesis^16^. Different from azurophilic granules, gelatinase-positive granules respond to relatively weak stimulation, a property that correlates with their earlier participation in the neutrophil response^8^. Based on the different impact of the exocytosis of granule subtypes on innate immunity and inflammation, the identification of exocytosis regulators with differential or opposing functions in the regulation of different granule subsets has important clinical significance; however, the mechanisms regulating neutrophil sequential exocytosis remain unknown.

Granule exocytosis in neutrophils is regulated by the trafficking regulatory protein Rab27a and its effectors UNC13D (Munc13-4) and JFC1/Slp1^17–19^. While Munc13-4 regulates docking of several granule subsets, JFC1 selectively regulates azurophilic granule secretion^19–21^. Just before exocytosis, dynamic azurophilic granules move to areas near the plasma membrane partially deprived of polymerized actin, maintaining an apparent actin-free environment in their surroundings, a mechanism regulated by granular-associated proteins including JFC1, a phosphatidyl-inositol 3-phospahate-binding adaptor^22^. Consequently, in JFC1-deficiency, azurophilic granules are trapped in polymerized actin and exocytosis is inhibited. Although this suggests that actin is a negative regulator of azurophilic granules exocytosis, the molecular mechanisms regulating granule-associated actin remodeling is unknown, and how a balance between putative positive and negative effects mediated by actin remodeling regulates the fate of different neutrophil secretory organelles needs further elucidation.

The Wiskott-Aldrich syndrome protein (WASP) family, including WASP, N-WASP, WAVE1-3, WHAMM, JMY and WASH, control actin cytoskeletal dynamics by regulating the actin nucleating activity of the Arp2/3 complex^23–25^. Different from other actin nucleators of the WASP family, WASH (Wiskott-Aldrich Syndrome Protein and Scar Homolog) interacts with intracellular organelles and is proposed to regulate not only actin remodeling but also endolysosomal trafficking mechanisms and lysosomal biogenesis^25–28^. FAM21, a protein known to stabilize WASH through its interaction with WASH N-terminus is a core component of the WASH complex^25^.

Despite the impact of neutrophil secretory proteins in human disease, a thorough molecular understanding of the regulation of neutrophil exocytosis is lacking. Here, we show that WASH differentially regulates secretion of cargoes from different neutrophil granule subsets favouring gelatinase granule exocytosis but maintaining the pro-inflammatory azurophilic granule in a refractory state. Our work identifies WASH as a molecular switch that regulates sequential exocytosis to control the timely neutrophil response and shows its dysfunction causes inflammation. The current study proposes that WASH activation is a potential therapeutic approach to target neutrophil-mediated inflammation.

## Results

### WASH localizes at neutrophil granules but *Wash*-deficiency does not cause quantitative granule or cargo alterations

Inhibition of actin polymerization in neutrophils dismantles the cortical actin barrier and facilitates granule access to the plasma membrane^20^, highlighting an inhibitory involvement of actin in exocytosis. However, although inhibition of actin polymerization does not abolish granule movement, it reduces motility and speed^20^, supporting that, in addition to acting as a barrier, actin remodeling exerts an active role in granule trafficking. How this putative dual role of actin remodeling regulates neutrophil exocytosis is currently unknown. Neutrophil azurophilic granules contain a molecular machinery that enables the dismantling of granule-associated actin to favor exocytosis during the activation of regulated secretion^20^. However, because exacerbated exocytosis of azurophilic granules is deleterious to the host, neutrophils maintain this granule type in a refractory state even under mild stimulatory conditions that permit the secretion of more responsive secretory organelles including gelatinase granules, and thus allowing for the initiation of the innate immune response without inducing inflammation. However, the molecular mechanisms that differentially regulate the exocytosis of neutrophil granules subtypes is currently unknown. To study this mechanism, we focused on the relationship between actin remodeling, trafficking and exocytosis of different granule subsets.

**Fig. 1:**
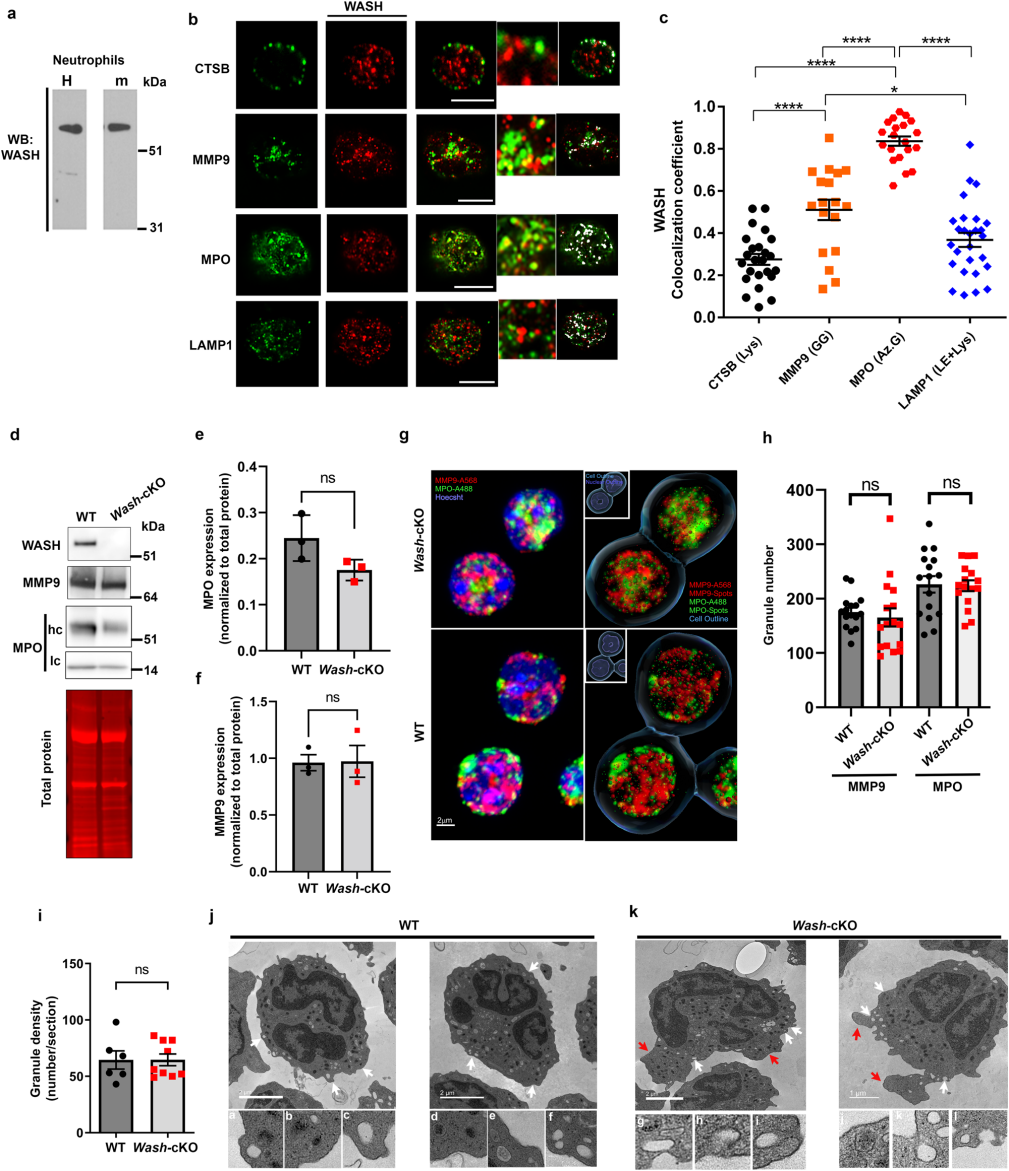
WASH associates with neutrophil granules. **a** Immunoblot analysis of WASH expression in human and mouse neutrophils. Representative of 3 independent samples. **b** Enhanced-resolution microscopy (Airyscan) of WASH related to neutrophil subcellular organelles by immunofluorescence of endogenous proteins. CTSB (cathepsin B); MMP-9 (matrix-metalloprotein 9); MPO (myeloperoxidase); LAMP1 (lysosome-associate membrane protein 1). Scale bar: 5 µm. Colocalized zones in cells are pseudo colored in white (binary). Representative of the data quantified in **c**. **c** Quantitative analysis of WASH localization at neutrophil organelles. *n* = 24, 18, 20 and 28 cells for CTSB, MMP-9, MPO and LAMP1 groups. Each symbol represents one cell. Representative of two independent experiments. Mean ± SEM, *p = 0.0172 and ****p < 0.0001. one-way ANOVA, Tukey’s multiple comparisons test. Lys (lysosome); GG (Gelatinase granule); Az. G, (azurophilic granule); LE (late endosome). **d** Immunoblot analysis of protein expression in wild-type (WT) and *Wash*-cKO *(Washc1*^*Δhaemo*^) neutrophils. MMP-9, (Gelatinase granule); MPO, (Azurophilic granule); hc and Ic, MPO heavy and light chains, respectively. Equal loading, Revert 700 Total Protein Stain. Representative of 3 independent mice quantified in **e**, **f**. **e**, **f** Quantitative analysis of MPO and MMP-9 expression, respectively. Mean ± SEM, *n* = 3 independent mice. Two-tailed Mann-Whitney test. **g** 3D quantification of granule numbers using Airyscan. Left, 3D stacks, presented as maximum intensity projections. Right, Imaris rendered 3D isosurfaces: cell outline (blue) and nuclei (in inset,) purple with quantified spots marking the centroids of the MMP-9 (red) and MPO (green) vesicles based on size and intensity. Scale bar: 2 µm. Representative of 3 independent experiments quantified in **h**. **h** Quantitative analysis of neutrophil granule numbers from 491 WT and 484 *Wash*-cKO cells,. Mean ± SEM, of 16 fields (symbols) containing 8 to 50 cells each. ns, not significant, Two-tailed Student’s *t-*test. **i** Transmission electron microscopy (TEM) analysis of neutrophil granule density. Mean ± SEM, Two-tailed Mann-Whitney test from 6 WT and 9 *Wash*-cKO micrographs. **j**, **k** TEM analysis of neutrophil morphology and granule fusion. **j** Wild-type and **k**, *Wash*-cKO neutrophils (two cells per group are shown, representative of 3 independent mice analyzed in two independent experiments). **j** (**a**–**f**), Granules appear distant from the plasma membrane in WT neutrophils. **k** (**g**–**k**), Docked or fused granules in *Wash*-cKO neutrophils. **k** (**l**), post-fusion event. White arrows, areas of magnification. Red arrows, areas of membrane expansion. **e**, **f**, **h** and **i**, ns, not significant. See Source Data file.

We first studied the possible regulation of neutrophil secretion by WASH. Here, we show that WASH is expressed in both human and mouse neutrophils (Fig. 1a). Next, we analyzed the subcellular localization of WASH by immunofluorescence analyses of endogenous proteins. We show that WASH localizes at neutrophil granules but not at the plasma membrane. In particular, WASH was found to distribute with a population of azurophilic granules and to colocalize with the granule marker myeloperoxidase (MPO) (Fig. 1b, c). FAM21 was also found to localize at azurophilic granules (Supplementary Fig. 1). Furthermore, WASH-deficiency, which does not affect FAM21 expression^25^, was associated with a significant decrease in FAM21 localization at this granule subset (Supplementary Fig. 1). WASH is also present at gelatinase granules in neutrophils, albeit to a lesser extent than at azurophilic granules (Fig. 1c). Similar to macrophages, WASH partially colocalized with the late endosome marker LAMP1 and with lysosomal cathepsin B in neutrophils (Fig. 1b, c). Neutrophils from *Vav1*-driven *Washc1-*knockout mice lacking WASH expression in the haemopoietic lineage (*Washc1*^*Δhaemo*^, hereon *Wash-*cKO) present normal expression levels of myeloperoxidase (Fig. 1d, e), and elastase (Supplementary Fig. 2), two azurophilic granule cargoes produced during the promyelocytic stage. The expression of MMP-9 (gelatinase granules), formed at a late stage in the maturation process, was also similar in WT and *Wash*-cKO neutrophils (Fig. 1d, f), suggesting normal granule maturation in *Wash*-deficient neutrophils. Furthermore, 3D reconstruction fluorescence microscopy analysis confirmed that the number of azurophilic and gelatinase granules in *Wash-*cKO neutrophils was not significantly different from the number of granules present in WT cells (Fig. 1g, h), further confirming that granulopoiesis is normal in neutrophils lacking WASH.

Next, we analyzed the morphology and ultrastructure of *Wash*-cKO neutrophils. Transmission electron microscopy (TEM) analysis confirmed that neutrophils lacking WASH present normal granularity (granule density, Fig. 1i). As expected, in wild-type cells, all granules appeared separated from the plasma membrane, at a distance of around ~80–180 nm (Fig. 1j, inserts a–f), which is thought to be mediated by an actin barrier^19^. This differed from that observed in *Wash-*cKO neutrophils where many granules are docked (Fig. 1k, insert g) or have fused (Fig. 1k, inserts h–k) with the plasma membrane, which in some areas appears to have been compromised (Fig. 1k, insert l), after an exocytosis event has taken place. Of note, although *Wash*-cKO neutrophils show areas with apparent abnormal plasma membrane expansion (Fig. 1k, red arrows), the spontaneous fusion events observed in *Wash*-cKO neutrophils do not take place at sites of membrane extensions, instead, many of these events were observed at sites of membrane bending (white arrows in Fig. 1k).

### *Wash*-deficiency causes exacerbated azurophilic granule exocytosis

**Fig. 2:**
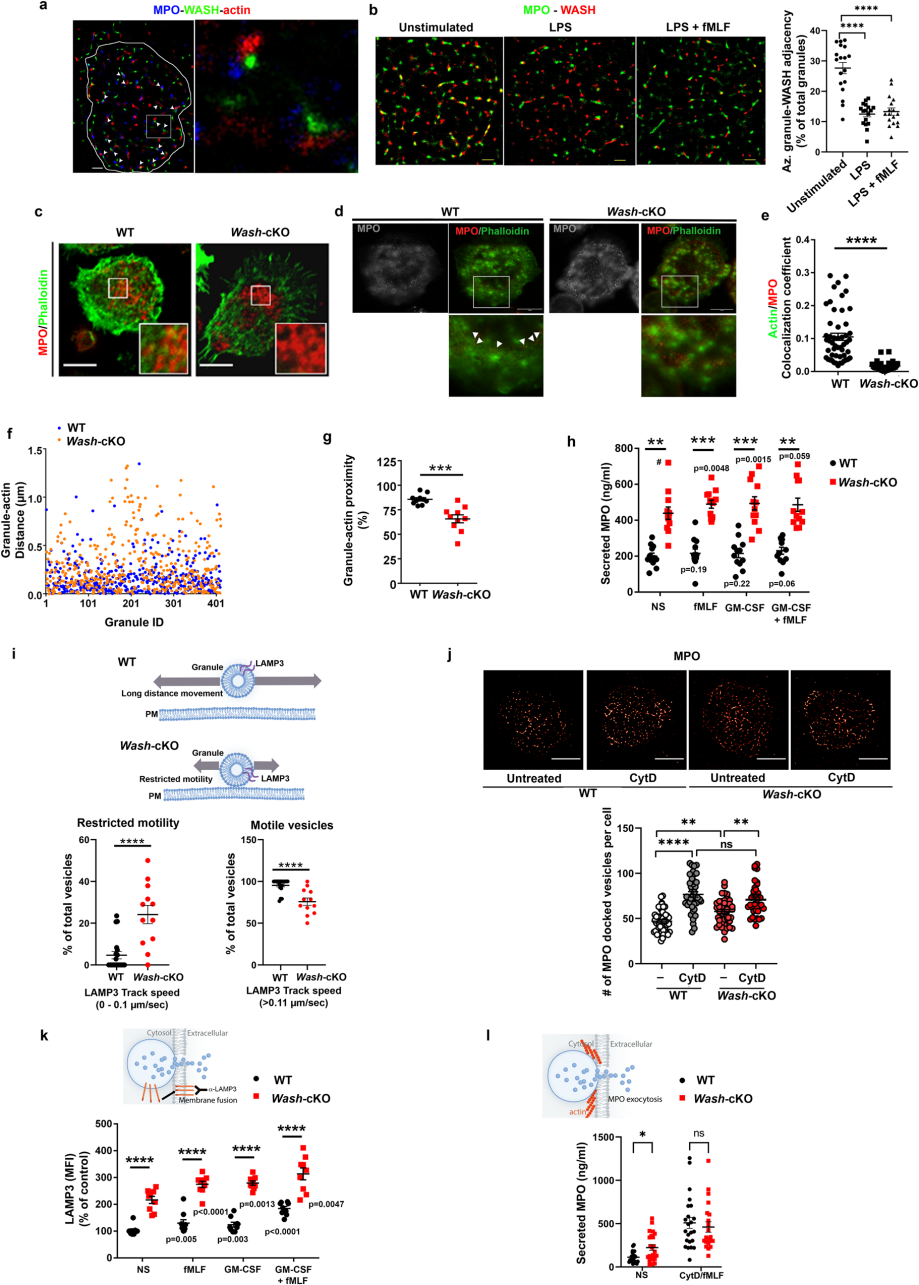
WASH is necessary for azurophilic granule entrapment in polymerized actin and its deficiency unlocks exocytosis of this granule subtype. **a** Super-resolution microscopy analysis (STORM) of the tripartite complex, WASH (green), F-actin (phalloidin, red), and azurophilic granules, (myeloperoxidase, MPO, blue) in wild-type (WT) neutrophils. Scale bar: 1 µm. Representative of two independent experiments. **b** Association of WASH with azurophilic granules in wild-type neutrophils. Left, STORM images. WASH (red), MPO (green). Scale bar: 1 µm. Right, Quantification of the proximity of WASH to the azurophilic granule marker under unstimulated (*n* = 18), primed (lipopolysaccharide, LPS) (*n* = 17) or stimulated (LPS + fMLF) (*n* = 17) conditions. Mean ± SEM of % of total granules adjacent (200 nm) to WASH molecular clusters per cell. Each symbol represents one cell. ****p < 0.0001. One-way ANOVA, Tukey’s multiple comparisons test. **c** Analysis of F-actin (green) and azurophilic granule (MPO, red) near the plasma membrane of WT and *Wash*-cKO *(Washc1*^*Δhaemo*^) neutrophils by confocal microscopy. Scale bar: 5 µm. *n* = 3, representative of the data quantified in **e**. **d** Super-resolution STED microscopy analysis of granule-actin network. Left, granules, shown in grey scale. Right, Arrowheads, azurophilic granule (MPO, red) entrapped in F-actin (phalloidin, green). Scale bar: 5 µm. Representative of 15 WT and 16 *Wash*-cKO neutrophils from 2 independent mice. **e** Quantification of F-actin distribution at the granule surrounding areas from confocal microscopy analysis shown in **c**. Mean ± SEM. ****p < 0.0001. Two-tailed unpaired Student’s *t*-test. *n* = 51 WT and 32 *Wash*-cKO cells from 3 independent mice. **f** Spot to Surface Distance analysis between granules and their most proximal actin fiber puncta. WT, 10 neutrophils (439 granules), *Wash*-cKO, 10 neutrophils (440 granules). **g** Percentage of azurophilic granules in each cell with actin puncta at <300 nm. Mean ± SEM. ***p = 0.0002, two-tailed unpaired Student’s *t*-test. *n* = 10. **h** Azurophilic granule exocytosis measured as secreted MPO. NS, non-stimulated. Mean ± SEM. WT vs *Wash-cKO*: NS **p = 0.0027; fMLF ***p = 0.0005; GM-CSF ***p = 0.0007; GM-CSF + fMLF **, 0.0039; Kruskal-Wallis ANOVA multiple comparisons. *n* = 12 independent mice. The *p* values in the figure correspond to stimulated conditions compared to their unstimulated controls (paired Student’s *t*-test, one-tailed). #, outlier (Grubb’s test). **i** Analysis of vesicular dynamics of neutrophil granules expressing EGFP-LAMP3, at the exocytosis active zone using TIRFM (Scheme). Vesicles were binned in two groups: restricted motility (docked, 0.1 μm/s) and high motility (>0.11 μm/s), and plotted as a percentage of total vesicles in each group for a given cell. Mean ± SEM from 20 WT cells and 12 *Wash-*cKO cells from two independent experiments. ****p < 0.0001. Two-tailed, unpaired *t*-test. **j** Docking of azurophilic granules in the exocytic active zone by TIRFM and Super-Resolution Radial Fluctuations (SRRF). Each symbol represents a cell. ns, not significant; Mean ± SEM. WT vs *Wash-*cKO, **p = 0.0085; *Wash-*cKO, untreated (–) vs cytochalasin D (CytD), **p = 0.0019; ****p < 0,0001 (one-way ANOVA, Tukey’s multiple comparisons test). *n* = 48, 42, 44 and 36 cells from three independent mice analyzed for each condition. Scale bar: 5 µm. **k** Analysis of granule fusion (LAMP3 translocation to the plasma membrane). *n* = 9 independent mice from 3 independent experiments. Mean ± SEM. ****p < 0.0001 (one-way ANOVA, Tukey’s multiple comparisons test). In figure *p* values: stimulated conditions compared to their unstimulated controls (one-tailed paired Student’s *t*-test). **l** MPO secretion after treatment with CytD and the indicated stimuli analyzed by ELISA. Mean ± SEM. *p = 0.0279; ns, not significant, Kruskal-Wallis ANOVA multiple comparisons uncorrected Dunn’s test; NS, not stimulated. Each symbol corresponds to an independent mouse (*n* = 23 wild-type and 24 *Wash*-cKO mice) from 3 independent experiments. ns, not significant. Source data are provided as a Source Data file.

To study the putative dynamic changes of WASH in association with neutrophil secretory organelles during neutrophil priming and activation, we first analyzed the distribution of WASH in relationship to F-actin and the azurophilic granules in wild-type cells by Stochastic Optical Reconstruction super-resolution Microscopy (STORM). We first show that, under unstimulated conditions, WASH appears as a tripartite complex together with F-actin and MPO at the exocytic active zone (130 nm from the plasma membrane) (Fig. 2a). Only around 20% of total azurophilic granules are exocytable under maximum stimulation with, for example, calcium ionophores^29^. Under physiological conditions, azurophilic granule secretion requires priming with, for example, LPS or GM-CSF, which amplify the neutrophil response to a second stimuli but are themselves weak exocytosis inducers. To test whether priming or stimulation alters the distribution of WASH related to the azurophilic granule, we quantified the proximity of WASH molecules to the nearest azurophilic granule cargo (MPO) using STORM. We found that priming with LPS (Fig. 2b) is sufficient to induce WASH withdrawal from azurophilic granules, and this phenotype is maintained after stimulation with the neutrophil chemotactic peptide, formyl-Met-Leu-Phe (fMLF) (Fig. 2b). Next, we analyzed the distribution of F-actin in relationship to neutrophil azurophilic granules in wild-type and *Wash*-cKO neutrophils. Azurophilic granules in *Wash*-cKO neutrophils were deprived of polymerized actin (Fig. 2c). This differed from that observed in wild-type neutrophils, in which azurophilic granules are surrounded by F-actin (Fig. 2c). To further visualize this phenomenon at higher resolution, we used STED (Stimulated Emission Depletion) super-resolution microscopy. Similar to that observed by confocal microscopy (Fig. 2c), STED imaging shows that while azurophilic granules are surrounded by polymerized actin in wild-type cells (Fig. 2d, white arrowheads), the azurophilic granules of *Wash*-cKO neutrophils were deprived of polymerized actin at their neighboring areas (Fig. 2d). To support these observations, we performed two independent quantitative analyses of the association of actin to azurophilic granules. In neutrophils, azurophilic granules are surrounded by F-actin, with overlapping (colocalized) pixels at the edges of granules but not inside the granules. First, we studied granule-actin adjacency, by analysis of their colocalization in the contiguous overlapping margins between granules cargoes and F-actin. We found a significant decrease in the amount of F-actin in close proximity to and in the surrounding areas of azurophilic granules of *Wash*-cKO neutrophils as compared to wild-type cells (Fig. 2e). Next, we use Spot to Surface Distance Transformation to analyze the distance between granule molecular cargoes and their most proximal actin molecular cluster. Again, this study confirmed that a significant number of azurophilic granules from *Wash*-cKO neutrophils are deprived (increased distance, Fig. 2f) of F-actin. This was also visualized as a net decrease in the number of granules with detectable F-actin at <300 nm from the granule (Fig. 2g). We hypothesized that azurophilic granules deprived of F-actin in *Wash*-cKO neutrophils, could gain access to the plasma membrane and engage in exocytosis, a scenario that, in wild-type cells, only occurs under the stimulatory conditions that favor the full dismantling of the actin network surrounding the azurophilic granule. To test this experimentally, we isolated highly purified mature neutrophils from wild-type and *Wash-*cKO mice and analyzed their ability to undergo exocytosis in response to a variety of priming agents and stimuli known to induce azurophilic granule exocytosis. We found that *Wash*-cKO neutrophils have a marked increase of azurophilic granule exocytosis under non-stimulatory conditions (Fig. 2h), as determined by the release of the azurophilic granule marker MPO. These data suggest that the inhibitory mechanism that prevents the release of highly toxic components from azurophilic granules in wild-type cells is not functional in *Wash*-cKO neutrophils. GM-CSF and the bacteria-derived mimicking peptide fMLF, are mild inducers of azurophilic granules secretion in wild-type neutrophils (Fig. 2h). The exacerbated release of azurophilic granule cargoes in *Wash*-cKO neutrophils in relationship to wild-type cells was maintained even under stimulated conditions (Fig. 2h). Because exocytosis assays were performed using neutrophils in suspension, the exacerbated secretion observed in *Wash-*cKO neutrophils was independent of putative defects in neutrophil attachment or caused by different levels of β_2_ integrin expression, which were normal in *Wash*-cKO neutrophils (shown below). This differs from the reported function of WASH in integrin (α5β1) recycling in invasive ovarian cancer cells^30^ and highlights unique mechanisms mediated by WASH in neutrophils. Furthermore, increased azurophilic granule exocytosis in *Wash*-cKO neutrophils was not caused by differential expression of granular proteins (Fig. 1d, e and Supplementary Fig. 2). Importantly, although GM-CSF and fMLF stimuli were individually too weak to induce secretion of this granule type in WT cells, they activate exocytosis in *Wash*-cKO neutrophils (Fig. 2h), suggesting that a subpopulation of granules that requires activation, remodeling or maturation of their secretory machinery through stimulation, is then able to exocyte in the absence of priming only if WASH is not present.

Next, we hypothesized that azurophilic granule docking at the plasma membrane could be increased in *Wash*-cKO neutrophils, a phenotype that was also suggested by the TEM data presented in Fig. 1. To test whether increased azurophilic granule exocytosis is mediated by increased docking and/or fusion, we studied LAMP3 (CD63), a granule membrane-associated protein that, in neutrophils, is present at the membranes of azurophilic granules^31^ and is translocated to the plasma membrane upon granule-plasma membrane fusion. Here, we first studied whether the defects in actin remodeling observed in the absence of WASH expression decreased granule motility associated with increased granule docking. To this end, we analyzed the trafficking of LAMP3-positive granules in wild-type and *Wash*-cKO neutrophils by Total Internal Reflection Fluorescence Microscopy (TIRFM), a technique that allows the visualization of organelles that reside in close proximity (∼100 nm) to the plasma membrane, while maintaining a high signal to background ratio. Quantitative analyses of vesicular trafficking showed increased numbers of azurophilic granules with restricted motility (speed < 0.1 µm/sec, docked) located adjacent to the plasma membrane in *Wash*-cKO neutrophils. (Fig. 2i, Supplementary Fig. 3 and Supplementary Movies 1 and 2). To further analyze whether the docking of azurophilic granules was increased in *Wash*-cKO neutrophils, we quantified the number of azurophilic granules localized proximal to the plasma membrane using a combinatorial TIRFM and Super-Resolution Radial Fluctuations (SRRF) approach. Here, we show that, under unstimulated conditions, *Wash*-cKO neutrophils have significantly higher numbers of azurophilic granules in the exocytic active zone compared to wild-type neutrophils (Fig. 2j) independently of cell spreading which was decreased in *Wash*-cKO neutrophils (shown below). Disruption of actin polymerization with cytochalasin D (Cyt D), a cell-permeable alkaloid that induces neutrophil exocytosis by permitting granule access to the plasma membrane^32^, increased access of granules into the exocytic active zone to similar levels in both wild-type and *Wash*-cKO neutrophils (Fig. 2j). Altogether, these data support the granule dynamics studies and suggest that WASH negatively regulates the docking of azurophilic granules to the plasma membrane.

Next, we asked whether excess azurophilic granule secretion in WASH deficiency was caused by increased fusion or could be explained instead by an increment in kiss-and-run exocytic events. To answer this, we analyzed azurophilic granule fusion at the plasma membrane by the detection of the extracellular domain of the granule membrane-associated protein LAMP3 (Fig. 2k). Using this assay, we established that the increased azurophilic granule exocytosis phenotype in *Wash*-cKO neutrophils was caused by full fusion of azurophilic granule membranes with the plasma membrane (PM) manifested as the increment of LAMP3 at the PM under both stimulated and unstimulated conditions (Fig. 2k). Of note, different to that shown in Fig. 2h, LAMP3 incorporation into the plasma membrane was evident even in the absence of priming in both WT and *Wash*-cKO cells, thus highlighting differences between fusion mechanisms and azurophilic granule cargo release. Despite these differences, our data indicate that the exacerbated secretion in the absence of WASH is manifested at both the fusion and cargo release levels.

To establish whether the increased azurophilic granule exocytosis observed in *Wash*-cKO neutrophils is caused by defective actin remodeling, we next treated wild-type and *Wash*-cKO neutrophils with cytochalasin D. In secretion assays, we found that only when treated with cytochalasin D, wild-type neutrophils showed high levels of azurophilic cargo secretion similar to those observed in *Wash*-cKO neutrophils (Fig. 2l). Importantly, inhibition of the Arp2/3 complex with the small molecule CK666 increased azurophilic granule exocytosis in wild-type cells (Supplementary Fig. 4A), further supporting that both genetic or pharmacological interference with actin nucleation increases azurophilic granule exocytosis. However, MPO secretion in wild-type cells after treatment with CK666 is significantly lower than the exocytosis observed in CK666-treated *Wash*-deficient neutrophils (Supplementary Fig. 4A), suggesting that WASH may negatively regulate docking and exocytosis by actin-dependent and independent mechanisms. Of note, neutrophils treated with either 50 or 150 µM CK666, showed similar levels of Arp2/3 complex inhibition measured as the decreased colocalization of F-actin with Arp2 (Supplementary Fig. 4B–E). CK666 also decreased the association of F-actin with azurophilic granules in wild-type cells to the levels observed in *Wash*-cKO cells at both 50 and 150 µM, and no differences were observed between treatments at these CK666 concentrations (Supplementary Fig. 4C). CK666 also decreased the localization of Arp2 at azurophilic granules in wild-type cells, at both concentrations, to a similar extent (Supplementary Fig. 4D), supporting that Arp2/3 regulates F-actin near or at azurophilic granules and that 50 µM CK666 is sufficient to inhibit the complex in these cells.

### WASH interferes with the Rab27a-JFC1 axis to impede azurophilic granule secretion

**Fig. 3:**
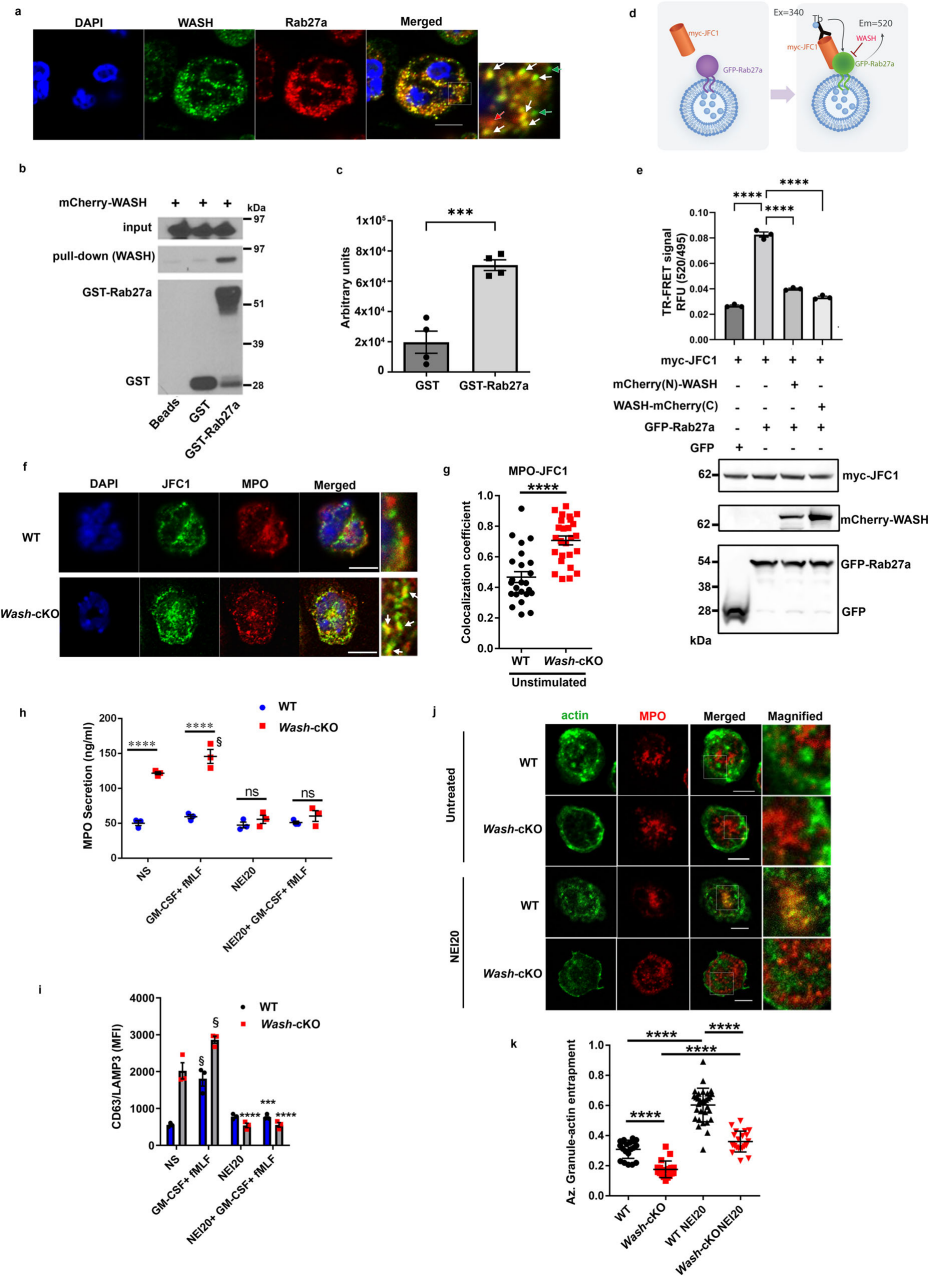
WASH interacts with the small GTPase Rab27a and controls the recruitment of JFC1 to azurophilic granules. **a** Immunofluorescence analysis of endogenous WASH and Rab27a in human neutrophils. Scale bar: 5 µm. Inset: White arrows denote colocalization. Red arrow, Rab27a-positive vesicles lacking WASH, green arrow, vesicles expressing WASH but lacking Rab27a. *n* = 3. **b** Pull-down assay, Rab27a and WASH interaction. Representative of 4 independent experiments quantified in **c**. **c** Quantification of pull-down assays. Mean ± SEM, *n* = 4. ***p = 0.0008. Two-tailed unpaired *t*-test. **d**, **e** TR-FRET assay of the binding of JFC1 to Rab27a in the presence or absence of WASH. **d** Schematic representation. **e** Upper panel, Quantitative analysis of TR-FRET reactions. Mean ± SEM of three biological replicates representative of two independent experiments. ****p < 0.0001. One-way ANOVA, Tukey’s multiple comparisons test. Bottom panel, Immunoblots of the tagged proteins representative of two independent experiments. **f** Immunofluorescence analysis of endogenous JFC1 localization at azurophilic granules (myeloperoxidase, MPO) in wild-type and *Wash*-cKO *(Washc1*^*Δhaemo*^) neutrophils. Representative of 3 independent experiments. Scale bar: 5 µm. **g** Quantitative analysis of **f**. Mean ± SEM. Each symbol represents an individual cell from one experiment, representative of 3 independent experiments, shown in Supplementary Fig. 5. *n* = 23 WT and 25 *Wash*-cKO cells. ****p < 0.0001. Two-tailed unpaired Student’s *t*-test. **h** Effect of the inhibition of Rab27a-JFC1 binding on azurophilic granule exocytosis in *Wash*-cKO neutrophils. Neutrophils were treated with the neutrophil-exocytosis specific inhibitor Nexinhib20 (NEI20) and stimulated with fMLF after GM-CSF priming. Mean ± SEM from 3 independent mice. NS, not stimulated; ns: not significant; ****p < 0.0001. one-way ANOVA Tukey’s multiple comparison test. **i** Analysis of vesicle fusion (LAMP3) with the plasma membrane in neutrophils treated with NEI20 and stimulated with fMLF with GM-CSF priming. *n* = 3 independent mice. ***p = 0.0002; ****p < 0.0001, NEI20 *vs* same condition without NEI20. one-way ANOVA, Tukey’s multiple comparisons. **j**, **k** Analysis of the effect Nexinhib20 on granule entrapment into F-actin. **j** Confocal microscopy images of F-actin (phalloidin) and azurophilic granules (MPO) representative of the data quantified in **k**. Scale bar: 5 µm. **k** Quantitative analysis of azurophilic granule entrapment. ****p < 0.0001. *n* = 20, 19, 31 and 21 cells from 3 independent mice. One-way ANOVA, Tukey’s multiple comparisons test. ^§^*p* = 0.05 vs unstimulated control (one tailed, paired, Mann Whitney test). Source data are provided as a Source Data file.

The small GTPase Rab27a is a master regulator of exocytosis and together with its effectors JFC1 and Munc13-4, they control the docking, fusion and exocytosis of azurophilic granules^19^. Because Rab27a regulates azurophilic granule exocytosis, and Rab27a effectors are postulated to regulate actin remodeling, we next studied a possible crosstalk between WASH-deficiency and Rab27a activation. First, we studied the distribution of WASH related to Rab27a-positive granules by immunofluorescence analysis of the endogenous proteins and found that WASH colocalizes at puncta with Rab27a both in neutrophils (Fig. 3a). This indicates that WASH and Rab27a are present at the same secretory organelle. Next, to analyze whether Rab27a and WASH physically interact, we performed pull-down analysis of WASH using either recombinant GST-Rab27a or GST as control. We found that Rab27a efficiently and specifically interacts with WASH (Fig. 3b). The interaction was further confirmed by quantitative analysis in four independent experiments (Fig. 3c). Because the interaction between Rab27a and JFC1 is essential for azurophilic granules to undergo exocytosis^33^, we next studied whether WASH interferes with this interaction. To this end, we used a time resolved-FRET assay, consisting of the analysis of the interaction between the Rab GTPases and its effector on intact organelles, thus preserving the natural environment where the interaction takes place (Fig. 3d)^33^. We found that WASH significantly interferes with the interaction of JFC1 with Rab27a (Fig. 3e). Next, we reasoned that if WASH interferes with the Rab27a-JFC1 interaction, the GTPase would more efficiently recruit the effector when WASH is absent. To analyze this, we performed immunofluorescence analysis of endogenous proteins and show significant increased recruitment of the Rab27a-effector JFC1 at MPO-positive azurophilic granules in *Wash*-cKO neutrophils as compared to wild-type cells (Fig. 3f, g), which is also manifested under stimulatory conditions (Supplementary Fig. 5). These data suggested that the binding of WASH to Rab27a at the granule membrane interferes with the recruitment of JFC1 by Rab27a. Because the interaction of Rab27a with JFC1 is essential for azurophilic granule secretion, the data also suggest that increased amounts of JFC1 interacting with Rab27a at azurophilic granule membranes may be partially responsible for the increased azurophilic granule exocytosis observed in WASH-deficient neutrophils.

To further investigate if the increased azurophilic granule exocytosis in *Wash*-cKO neutrophils is Rab27a- and JFC1-dependent, we utilized a recently developed neutrophil exocytosis inhibitor, Nexinhib20 (NEI20), that interferes with the Rab27a-JFC1 interaction^33^. Here, we show that treatment with NEI20 decreases azurophilic granule exocytosis in *Wash*-cKO neutrophils as measured by two independent approaches: MPO secretion (Fig. 3h) and the upregulation of LAMP3 (CD63) at the plasma membrane (Fig. 3i). Nexinhib20 inhibited both stimulated and unstimulated secretion in *Wash*-cKO neutrophils, further supporting that exacerbated exocytosis in these cells is mediated by the dysregulation of the Rab27a/JFC1 secretory machinery. Next, we showed that treatment with Nexinhib20 increased the amount of F-actin localized at azurophilic granules (Fig. 3j, k) supporting the previous finding that the Rab27a-dependent secretory machinery, which includes JFC1, decreases localized actin polymerization in the areas surrounding the azurophilic granules during exocytosis^20^. We also show here that azurophilic granule entrapment in polymerized actin is likely a WASH-dependent mechanism because *Wash*-cKO neutrophils showed decreased entrapment compared to wild-type cells (Fig. 3k). However, although, NEI20 partially increased granule entrapment in the F-actin network in the absence of WASH (Fig. 3k, *Wash-cKO* vs *Wash-cKO* NEI20), *Wash*-cKO neutrophils showed lower entrapment even after NEI20 treatment compared to NEI20-treated wild-type cells, indicating that the difference in F-actin association with MPO granules in this model is indeed independent of the Rab27a-JFC1 interaction. Altogether, our data support a dual mechanisms regulated by WASH: On the one hand, WASH inhibits azurophilic granule secretion by an actin-dependent mechanism; on the other, WASH blocks azurophilic granule exocytosis by preventing vesicular docking, a mechanism mediated by inhibition of the recruitment of JFC1 to the azurophilic granule, a molecule known to regulate docking through the binding of its C2A domain to phosphoinositides present in the plasma membrane^22^, which is further supported by the observed increased docking of azurophilic granules in WASH-deficient cells (Fig. 2j).

### WASH is necessary for the formation of actin comets and gelatinase granule exocytosis

**Fig. 4:**
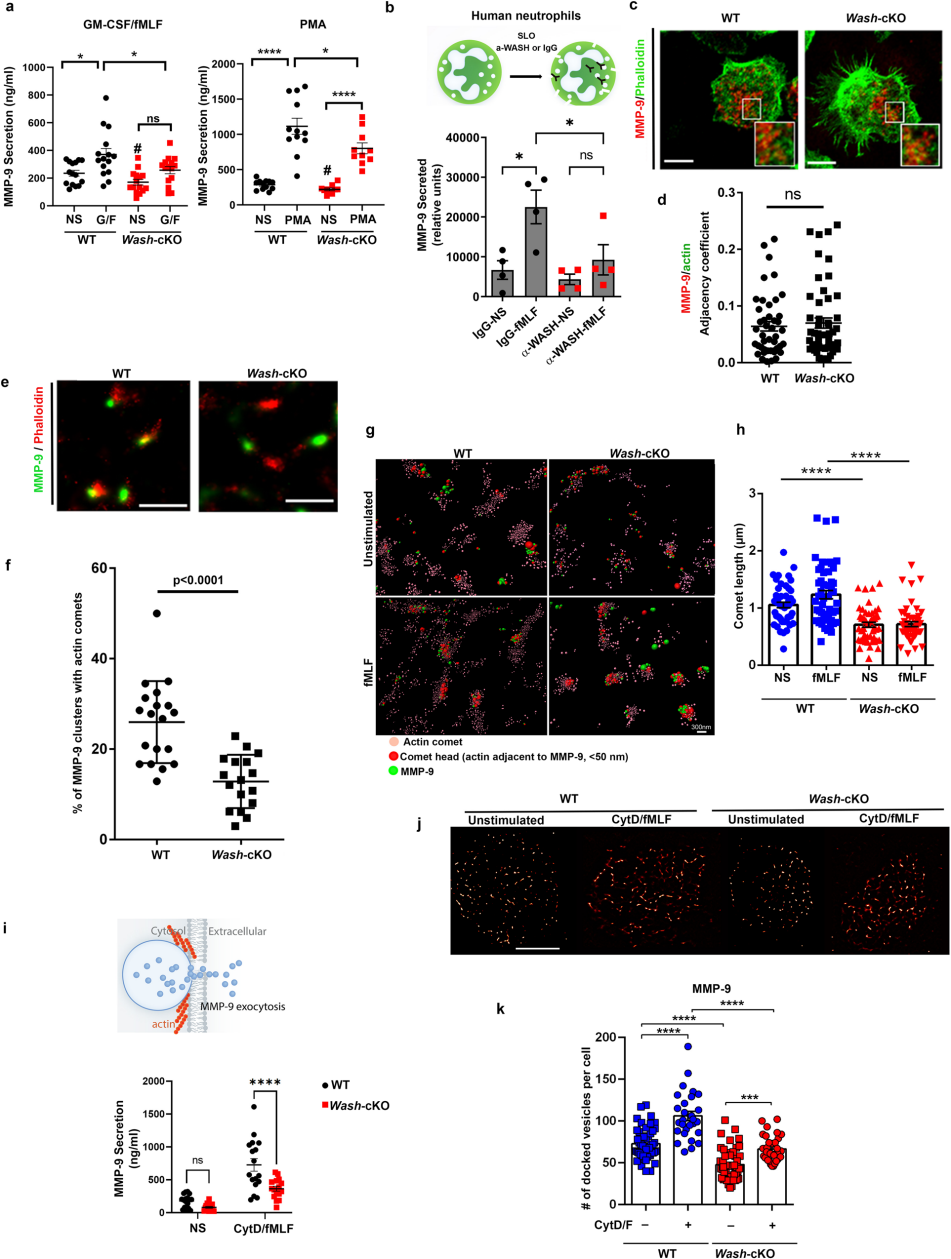
WASH forms actin comets polarized at gelatinase granules and *Wash*-deficiency impairs exocytosis of this granule subtype. **a** Gelatinase granule (MMP-9) exocytosis. Mean ± SEM. Left panel, *n* = 15 independent WT and *Wash-cKO* mice; Right panel, 12 WT, and 10 *Wash-cKO* independent mice analyzed in 4 independent experiments. Left panel, G/F (GM-CSF/formyl-Met-Leu-Phe). WT non stimulated (NS) vs G/F: *p = 0.01; WT vs *Wash*-cKO (G/F): *p = 0.042. ns, not significant. Right panel, WT vs *Wash*-cKO (PMA): *p = 0.0217; ****p < 0.0001. #, not significant vs WT unstimulated. **b** Gelatinase granule exocytosis in human neutrophils permeabilized with streptolysin-O (SLO) in the presence of anti-WASH inhibitory antibodies or IgG control. Mean ± SEM. *p = 0.0176 (IgG-NS vs. IgG-fMLF) and *p = 0.0481 (IgG-fMLF vs. α-WASH-fMLF). *n* = 4 healthy donors. **c** Distribution of F-actin (Phalloidin, green) and gelatinase granules (MMP-9, red) in proximity to the plasma membrane of wild-type (WT) and *Wash*-cKO *(Washc1*^*Δhaemo*^) neutrophils, analyzed as in Fig. 2c. Scale bar: 5 µm. Representative of 3 experiments. **d** Quantitative analysis of granule-actin adjacency, which detects the contiguous overlapping margins between granules and F-actin. *n* = 44 WT and 52 *Wash*-cKO cells. Mean ± SEM. ns, not significant. Two-tailed unpaired *t*-test. **e** Super-resolution STORM microscopy analysis of F-actin (phalloidin, red) comets in association with MMP-9-granule molecular clusters (green) in unstimulated wild-type and *Wash*-cKO neutrophils. Scale bar: 1 µm. *n* = 3 experiments. **f** Quantitative analysis of the number of MMP-9 clusters associated with actin comets from 19 WT and 17 *Wash-*cKO cells from 2 independent experiments. Mean ± SD. Two-tailed unpaired t-test. **g** Representation of comets by localization coordinates for single molecule actin clusters reconstructed according to their polarization and size. Representative of the data quantified in h. Scale bar: 300 nm. **h** Quantification of comet length. Each symbol represents one comet from 5 independent cells either not stimulated (NS) or stimulated with fMLF. A total of 50 comets per condition were analyzed. Mean ± SEM. ****p < 0.0001. **i** Effect of cytochalasin D treatment on gelatinase granules exocytosis. Upper panel, Representative scheme. Lower panel, Secreted MMP-9 was analyzed by ELISA. NS, not stimulated, CytD/F, cytochalasin D + fMLF. *n* = 17 WT and 16 *Wash*-cKO mice. Mean ± SEM. ****p < 0.0001. Four independent experiments. **j**, **k** Analysis and quantification of the distribution of gelatinase granules in the exocytic active zone by TIRFM and SRRF. Scale bar = 5 µm. Each symbol represents a cell treated with cytochalasin D/fMLF or vehicle. Mean ± SEM. ***p = 0.0005; ****p < 0,0001. A total of 28 to 48 cells per group were analyzed. **a**, **b**, **h**, **i** and **k**, one-way ANOVA multiple comparisons test (Tukey’s). ns, not significant. Source data are provided as a Source Data file.

The secretion of neutrophil granule subsets is sequential. Thus, secondary and tertiary granules are involved in neutrophil functions that precede the functions executed by the cargoes of azurophilic granules. In neutrophils, this is regulated by the increased susceptibility of secondary and tertiary granules to undergo exocytosis, which is achieved by the relatively weaker stimulation required to mobilize these granule subsets as compared to azurophilic granules. Here, to investigate the role of WASH in the regulation of secondary/tertiary granule exocytosis, we analyzed the secretion of the cargo matrix MMP-9 (metalloproteinase-9/gelatinase B), which is present at both these granule subsets but is absent from azurophilic granules and is expressed at normal levels in *Wash*-cKO neutrophils (Fig. 1d, f). We found that MMP-9 secretion is not increased under basal, unstimulated, conditions, in WASH-deficiency (Fig. 4a). Furthermore, basal secretion of secondary granules was also not affected as determined by quantitative analysis of lactoferrin and neutrophil gelatinase-associated lipocalin (NGAL), two markers of specific granules (Supplementary Fig. 6). However, *Wash*-cKO neutrophils present defective MMP-9 secretion in response to stimuli. Thus, significantly impaired secretion of MMP-9 was observed both in response to the physiological stimuli GM-CSF and fMLF, and when neutrophils were treated with the phorbol ester PMA (Fig. 4a). Despite these differences, mass spectrometric analysis of the secretomes of wild-type and *Wash*-cKO neutrophils show that differences in gelatinase granule exocytosis are driven by functional differences but not qualitative cargo composition as gelatinase granules components were commonly detected in the secretomes of both cell types (Supplementary Table 1). Thus, mass spectrometry, identified mostly common cargoes including lactoferrin, protein S100-A9, pre-complement component C3 and NGAL in both wild-type and *Wash-*cKO supernatants. We also identified neutrophil secretory factors, including epithelin 1 and 2, which were not identified in previous analysis of neutrophil granules, in both wild-type and *Wash-*cKO secretomes. These data suggested that defective gelatinase granule exocytosis does not affect, selectively, a subpopulation of granule subtype with specific cargoes in *Wash-*cKO cells but instead is likely caused by functional differences in the secretory machinery.

To analyze whether WASH regulates gelatinase granule exocytosis in human neutrophils, we utilized streptolysin O (SLO)‐permeabilized human polymorphonuclear leukocytes and anti-WASH inhibitory antibodies (Fig. 4b), a method previously utilized to characterize the function of other components of the neutrophil secretory machinery^20^. Here, we show that inhibition of WASH significantly impairs MMP-9 secretion in human neutrophils (Fig. 4b), thus recapitulating the phenotypes observed in *Wash-*cKO mouse cells. Of note, WASH inhibitory antibodies increased azurophilic granule exocytosis (Supplementary Fig. 7), further confirming the phenotype observed in *Wash-*cKO neutrophils and supporting the idea that exocytosis dysregulation is mediated by defects triggered by the absence of functional WASH, not by maturation defects caused by WASH deficiency.

To further understand the relationship between actin remodeling and gelatinase granule exocytosis, we next performed immunofluorescence analysis of the distribution of F-actin related to endogenous MMP-9, as described in Fig. 2 for MPO. Different from that observed for azurophilic granules, gelatinase granules proximal to the plasma membrane were surrounded by F-actin in unstimulated *Wash-*cKO cells (Fig. 4c). Quantitative analysis of granule-actin adjacency at relatively low resolution showed no significant differences in the distribution of F-actin relative to gelatinase granules between wild-type and *Wash-*cKO cells (Fig. 4d), an observation that correlates with the absence of spontaneous secretion of MMP-9 under unstimulated conditions.

Next, we analyzed the distribution of F-actin single molecular clusters at gelatinase granules using a super-resolution approach. We observed F-actin foci polarized, in comet shapes, at MMP-9-positive granules in wild-type cells (Fig. 4e). We suggest that this polarized distribution may have a positive role on granule movement by inducing propulsion through the formation of structures previously referred to as comet tails^34^. Contrarily to that observed in wild-type cells, actin foci where localized surrounding MMP-9 cluster molecules in *Wash-*cKO cells, but granule-associated actin comets are not properly assembled in *Wash-*cKO neutrophils (Fig. 4e, f). Quantitative analysis of actin polarized around granule protein clusters show increased numbers of comets in wild-type neutrophils as compared to *Wash-*cKO cells (Fig. 4f). Next, to further analyze comet formation, localization coordinates for single molecule actin clusters were reconstructed and comets analyzed according to their polarization and size (Fig. 4g). In this assay, single molecular clusters obtained from all fluorescent N-storm confirmed blinks, which are previously filtered for drift and background signals as a sphere on an image grid, were imported and a localization coordinate map built, where the centroid of the sphere is the central coordinate position in three-dimensional space, and the diameter of the sphere (spot) is the localization accuracy error (Fig. 4g)^35^. Actin spots that lie within a defined distance from the MMP-9 signal (<50 nm) were considered in contact with the granule marker (red in Fig. 4g) and distant spots that form the comets were represented in pale red. Using this approach, we established that, compared to *Wash*-cKO neutrophils, wild-type cells are characterized by increased comet sizes (Fig. 4g, h). We also established that comet size is significantly decreased in *Wash*-cKO neutrophils under both basal conditions and after stimulation with the bacteria-derived mimetic peptide fMLF (Fig. 4g, h) suggesting that WASH regulates the formation of actin comets which may help regulate the exocytosis of gelatinase granules by recruiting granules to the plasma membrane.

To better understand the crosstalk between WASH function, actin polymerization and gelatinase granule exocytosis, we treated wild-type and *Wash*-cKO neutrophils with the cell-permeable inhibitor of actin polymerization cytochalasin D, which increases secretion of gelatinase granules by disrupting cortical actin and by allowing access of the granules to the plasma membrane^32^. Here, we show that although treatment with cytochalasin D increased the secretion of MMP-9 in both wild-type and *Wash*-cKO neutrophils, the actin depolymerizing agent was unable to increase gelatinase B secretion in *Wash-*cKO cells to the levels observed in wild-type cells (Fig. 4i), further indicating that WASH regulates azurophilic and gelatinase granule exocytosis in different ways. In principle, depolymerization agents would inhibit both the construction of a cortical actin barrier and actin comets that mediate granule propulsion. So why doesn’t cytochalasin D treatment equalize gelatinase granule exocytosis in wild-type and *Wash*-cKO neutrophils? To answer this question, we performed analysis of vesicular docking, a process that is induced by cytochalasin D treatment as demonstrated by transmission electron and fluorescence microscopy^20^. To analyze this in further detail, we quantified the number of gelatinase granules proximal to the plasma membrane using super-resolution analysis of vesicular docking at <100 nm. Using this combinatorial TIRFM and Super-Resolution Radial Fluctuations (SRRF) approach, we found that wild-type cells show significantly higher numbers of gelatinase granules in the exocytic active zone than *Wash*-cKO neutrophils even under unstimulated conditions (Fig. 4j, k). Disruption of cortical actin allows for the increased access of granules into the exocytic active zone in both wild-type and *Wash*-cKO neutrophils but differences between the two groups were further exacerbated (Fig. 4j, k), suggesting that WASH is necessary to recruit a primed pool of gelatinase granules to the exocytic active zone. This agrees with the difference in exocytosis observed after treatment with cytochalasin D observed in Fig. 4i and with the observation that wild-type neutrophils show increased comet formation polarized at gelatinase granules even under unstimulated conditions (Fig. 4g, h). The decreased number of gelatinase granules at the exocytic active zone in both untreated and cytochalasin D-treated *Wash*-cKO cells (Fig. 4j, k), suggest that gelatinase granules of WASH-deficient neutrophils may be altered before they access the exocytic active zone despite no differences in cargo or granule maturation.

**Fig. 5:**
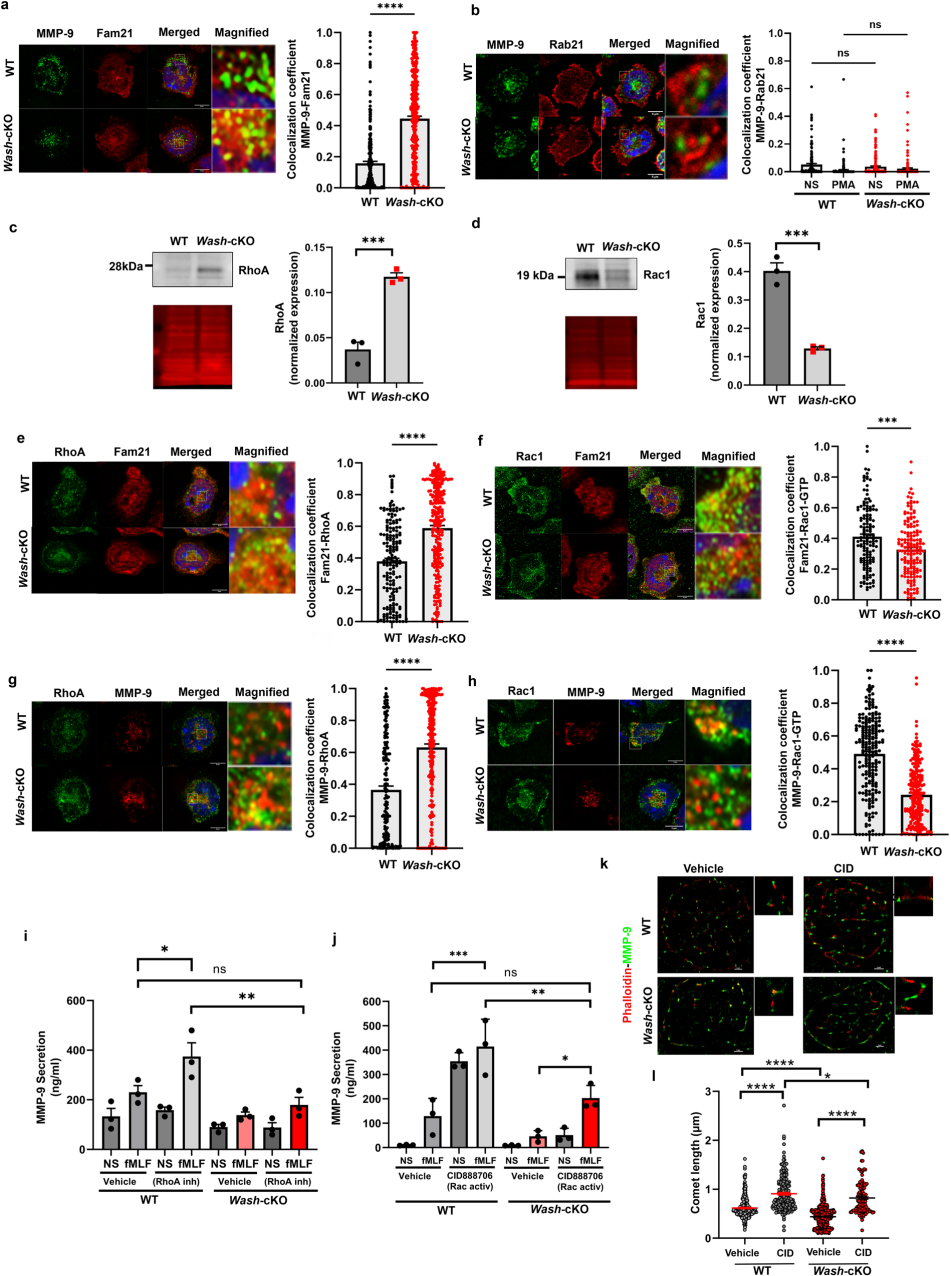
RhoA/Rac1 inversion is associated with impaired gelatinase secretion in *Wash*-deficiency. **a** Immunofluorescence analysis of the localization of endogenous FAM21 at gelatinase granules (MMP-9). Left, representative Airyscan images of WT and *Wash*-cKO (*Washc1*^*Δhaemo*^) neutrophils; Right, Quantitative analysis from 3 independent mice. A total of 165 WT and 169 *Wash*-cKO cells were analyzed. Mean ± SEM. ****p < 0.0001. **b** Analysis of the localization of Rab21 at gelatinase granules. Left, representative Airyscan images of WT and *Wash*-cKO neutrophils; Right, Quantitative analysis from 3 independent mice. A total of 152 and 188 WT, and 205 and 176 *Wash*-cKO, not stimulated (NS) or stimulated (PMA) cells, respectively, were analyzed. Mean ± SEM. Immunoblot analysis of the endogenous expression of total RhoA (**c**) and Rac1 (**d**) in neutrophils. Left, representative immunoblots (grey) and total protein loading (red). Right, Mean ± SEM, 3 independent mice. **c** ***p = 0.001; **d** ***p = 0.0007. **e**, **f** Colocalization of endogenous RhoA and Rac1-GTP with FAM21, respectively, by immunofluorescence. Left, representative Airyscan images of WT and *Wash*-cKO neutrophils; Right, Quantitative analysis from 3 independent mice. Mean ± SEM. ***p = 0.0001 and ****p < 0.0001. A total of 99 and 103 (**e**) and 107 and 117 (**f**) WT and *Wash*-cKO cells were analyzed. **g**, **h** Immunofluorescence analysis of the localization of RhoA and Rac1-GTP at gelatinase granules (*n* = 3). ****p < 0.0001. A total of 176 and 269 (**g**) and 100 and 115 (**h**) WT and *Wash*-cKO cells were analyzed. **a**–**h** Scale bar: 5 µm. **a**–**h** Two-tailed unpaired Student’s *t*-test. Three independent experiments. **i**, **j** Effect of RhoA inhibition (cell-permeable C3 Transferase, 2 µg/m) and Rac1 activation (CID888706, 10 µM) on gelatinase granule exocytosis (MMP-9 secretion). Mean ± SEM, *n* = 3. **i** *p = 0.0428; **p = 0.0038; **j** *p = 0.0410; **p = 0.0037 and ***p = 0.0002; ns, not significant. Ordinary one-way ANOVA, Tukey’s multiple comparisons test. **k**, **l** Effect of Rac1 activation on the actin comet defective phenotype of *Wash*-deficient neutrophils. **k** STORM super resolution images of actin comets (phalloidin, red) at gelatinase granules shown as MMP-9 molecular clusters (green). Representative of the data analyzed in **l** from three independent mice. Scale bar: 1 µm. **l** Quantitative analysis of actin comet length described in **k**. Mean ± SEM, from the analysis of 17, 13, 18 and 12 cells and 292, 205, 498 and 128 comets, from wild-type, Rac1-activated wild-type, *Wash*-cKO and Rac1-activated *Wash*-cKO cells respectively. CID, CID888706. Ordinary one-way ANOVA, Tukey’s multiple comparisons test. One outlier value in the WT-vehicle group (Grubb’s alpha = 0.05), length = 4.3 µm, (out of scale). Where indicated, *p = 0.0168; ****p < 0.0001. ns, not significant. Source data are provided as a Source Data file.

To analyze whether the molecular constitution of the trafficking molecules associated to gelatinase granules in WASH-deficiency is modified before engaging in exocytosis, we analyzed their endogenous molecular composition. FAM21 is significantly increased in gelatinase granules of *Wash*-cKO neutrophils (Fig. 5a). This represents an inverted phenotype to that observed in azurophilic granules in which FAM21 was significantly decreased in the absence of WASH (Supplementary Fig. 1). Because WASH complex subunits, including FAM21, were demonstrated to directly interact and colocalize with the small GTPase Rab21^36^, and Rab21 is both known to regulate neutrophil function^37^ and shown to mediate inhibition of secretion in myeloid cells^38^, we next analyzed whether increased FAM21 affected Rab21 localization at gelatinase granules. We show that Rab21 localizes mainly at the plasma membrane of both wild-type and *Wash*-cKO neutrophils with minimal recruitment of Rab21 to gelatinase granules under either resting or stimulated (Fig. 5b) conditions, suggesting that it is highly unlikely that Rab21 is a regulator of trafficking of this granule subtype. Next, we focused on the Ras GTPases RhoA and Rac1, both shown to interact with the WASH complex^39,40^. Western blot analysis of endogenous protein expression showed increased RhoA expression but significantly decreased expression of Rac1 in *Wash*-cKO neutrophils (Fig. 5c, d). Subcellular localization analysis of the endogenous proteins shows increased FAM21 colocalization with RhoA in *Wash*-cKO neutrophils, while its colocalization with Rac1 was decreased (Fig. 5e, f). This correlated with a marked increase of RhoA but decreased localization of active Rac1 at gelatinase granules in WASH-deficient neutrophils (Fig. 5g, h). We next analyzed the effect of Rac1 and RhoA function in gelatinase granule secretion. Treatment with the RhoA inhibitor CT04 significantly increased secretion in wild-type cells but only moderately in *Wash*-cKO cells (Fig. 5i). Contrarily, the activation of Rac1 using CID888706, a small molecule that increases the pool of active Rac1 to the levels observed with physiological agonists in several cellular systems^41^, activates Rac1 with five times more potency than Cdc42 (EC_50_: wild-type Rac1 = 20.17 nM; wild-type Cdc42 = 100nM^42^ (https://www.ncbi.nlm.nih.gov/books/NBK47359/table/ml099.tu1/) and, in our hands, markedly activates Rac1 (Supplementary Fig. 8), significantly increased gelatinase secretion in *Wash*-cKO cells under fMLF stimulation conditions, which reached the levels observed in wild-type cells treated with fMLF in the absence of exogenous Rac1 activation (Fig. 5j). Both RhoA inhibition and Rac1 activation also increased gelatinase granule exocytosis in wild-type cells beyond the levels observed in *Wash*-cKO cells (Fig. 5i, j). These differences are partially explained by the relative increased expression levels of total RhoA and decreased levels of total Rac1 present in *Wash*-cKO cells (Fig. 5c, d). Recent research indicated that Rac1 activity is necessary for the rapid formation of signal-dependent actin filamentous structures in the cell periphery, and the specific characteristics of these structures led to the suggestion that they are formed without Arp2/3 involvement^43^. Our results showing that *Wash*-cKO neutrophils present decreased active Rac1 and reduced actin comet formation, together with the observation that treatment with CK666 does not inhibit MMP9 secretion (Supplementary Fig. 9) suggest that decreased Rac1 recruitment to gelatinase granules together with impaired formation of actin comets in *Wash*-deficient cells are major contributors to the secretory defect of gelatinase granules. Supporting this, using quantitative super-resolution microscopy, we show that Rac1 activation rescues the phenotype in *Wash*-cKO neutrophils manifested as significant increases in the length of actin comets associated with gelatinase granules (Fig. 5k, l). Because the activation of Rac1 increases comet length and enhances secretion in the absence of WASH, it is possible that these structures are formed without Arp2/3 involvement as suggested before^43^. However, the putative compensation of other nucleation promoting factors (NPF), such as WAVE and N-WASP, for the absence of WASH cannot be ruled out at this time; however, treatment with 50 or 150 µM CK666 does not inhibit MMP9 secretion (Supplementary Fig. 9A, B). Of note, the Rac1 activator is also reported to activate Cdc42 albeit with less potency. In this regard, the participation of Rac1 not Cdc42 in the process under study in this work is more likely because a) in addition to the activator effect, we observed a decreased presence of Rac1 at the granules in the *Wash*-cKO cells and b) inhibition, not activation of Cdc42 is reported to activate neutrophil granule secretion^44^ albeit not tested in gelatinase granules. Altogether, our data indicate that gelatinase granules in WASH-deficiency have an altered molecular composition of trafficking molecules before engaging in exocytosis, and that RhoA/Rac1 inversion, decreased Rac1-GTP levels at gelatinase granules and decreased comet formation may all contribute to the defective secretory phenotype of gelatinase granules in *Wash*-deficiency.

### WASH-deficiency impairs neutrophil function including the production of reactive oxygen species and cell migration

**Fig. 6:**
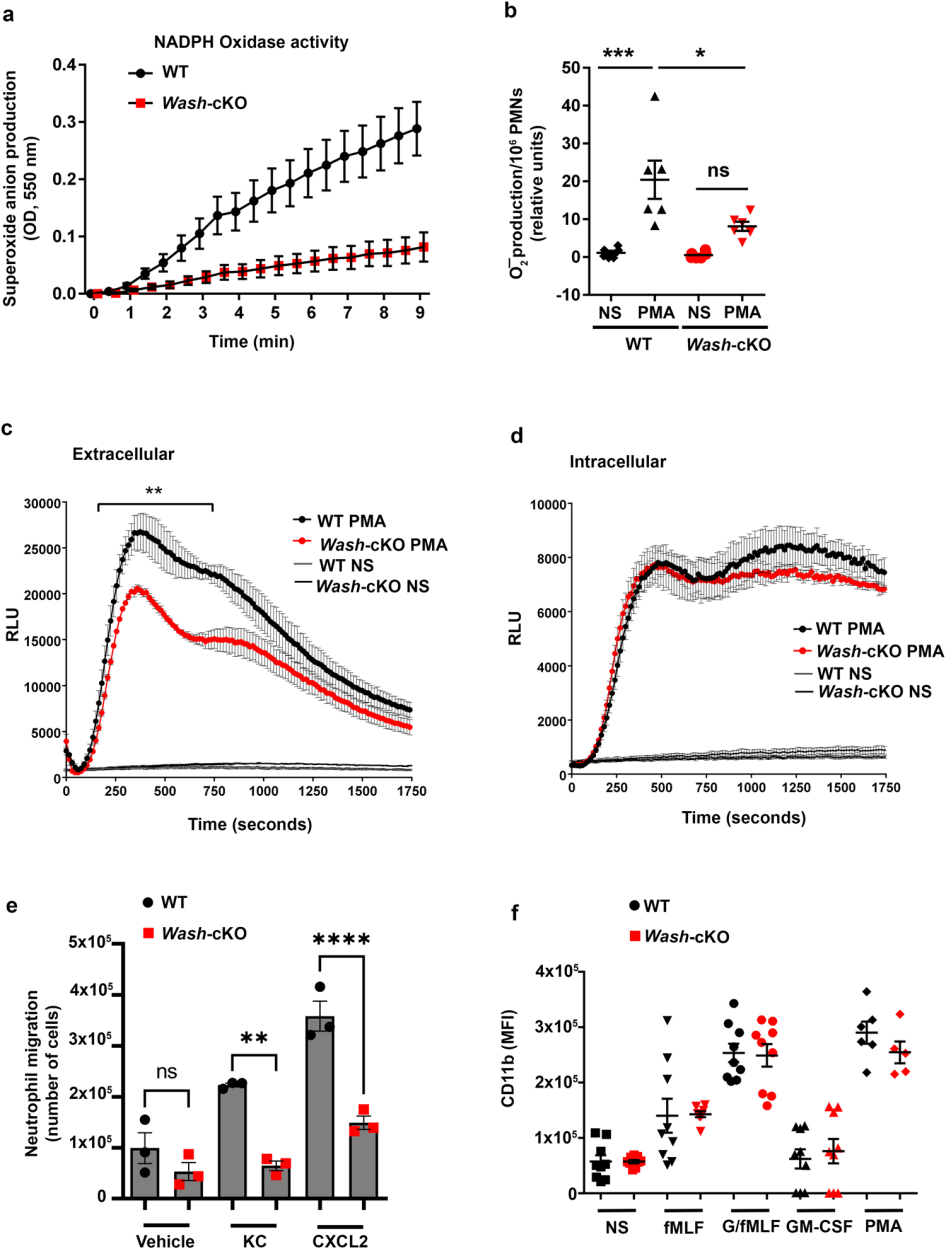
*Wash*-deficiency is associated with impaired ROS production and migration. **a** Representative kinetic analysis of the production of superoxide anion by WT and *Wash*-cKO *(Washc1*^*Δhaemo*^) neutrophils stimulated with PMA and measured by the cytochrome *c* reduction assay. Mean ± SEM, *n* = 3. **b** Quantitative analysis of superoxide anion production. Each symbol represents an individual mouse from 2 independent experiments. *n* = 6. Mean ± SEM. *p = 0.0166; ***p = 0.0002. One-way ANOVA, Tukey’s multiple comparisons. **c** Study of the production of extracellular reactive oxygen species by WT and *Wash*-cKO neutrophils as analyzed by chemiluminescence using the cell impermeant probe isoluminol. Mean ± SEM, *n* = 3. **p = 0.0193 (area under the curve), two-tailed unpaired Student’s *t*-test; **d** Study of the production of total reactive oxygen species by WT and *Wash*-cKO neutrophils as analyzed by chemiluminescence using the cell permeant probe luminol. Mean ± SEM, *n* = 3. **e** Analysis of wild-type and *Wash*-cKO neutrophil migration in response to CXCL1 (KC) and CXCL2. Mean ± SEM, *n* = 3. **p = 0.0012 and ****p < 0.0001. One-way ANOVA, Tukey’s multiple comparisons. **f** Analysis of the plasma membrane expression of the *β*_2_ integrin subunit CD11b in wild-type and *Wash*-cKO neutrophils by flow cytometry. Each symbol corresponds to an individual mouse from 3 independent experiments. Mean ± SEM. *n* = 9 for all conditions except PMA (*n* = 6). ns, not significant. Source data are provided as a Source Data file.

Neutrophils produce superoxide anion through the activation of the NADPH oxidase, which dismutates to hydrogen peroxide and is subsequently utilized by MPO to produce stronger oxidants. Because most of the membrane-associated subunits of the NADPH oxidase reside at gelatinase/specific granules, it is suggested that exocytosis and the oxidase are functionally linked. To establish the functional impact of defective secondary/tertiary granule exocytosis in WASH-deficiency, we studied the NADPH oxidase, whose activation at the plasma membrane depends on the translocation of its membrane-associated subunit, the cytochrome *b*_558_ (p22^*phox*^/gp91^*phox*^), from the gelatinase and secondary granules to the plasma membrane. To this end, neutrophils from wild-type and *Wash*-deficient mice were analyzed using the cytochrome *c*-reduction assay, the gold standard method to detect extracellular superoxide anion production in neutrophils. Here we show that the production of superoxide anion is significantly impaired in *Wash*-cKO neutrophils (Fig. 6a, b), a phenotype that correlates with the decreased fusion of gelatinase-positive granules with the plasma membrane. To further understand the defective production of ROS in *Wash*-cKO neutrophils we utilized a chemiluminescence-based assay for the detection of reactive oxidative species, products of both the NADPH oxidase and myeloperoxidase. Using the cell-impermeant probe isoluminol, we confirmed that extracellular ROS production is significantly impaired in *Wash*-cKO neutrophils despite exacerbated MPO exocytosis (Fig. 6c). Contrarily, intracellular ROS production in response to phorbol ester and detected by the cell-permeant probe, luminol, was not significantly affected in *Wash*-cKO neutrophils (Fig. 6d) supporting that defective gelatinase/secondary granule exocytosis and impaired NADPH oxidase activation are functionally linked in WASH-deficiency.

Because impaired actin remodeling and defective protease release are associated with impaired exocytosis and defective uropod detachment, respectively, and therefore it may affect neutrophil chemotaxis, we next analyzed the ability of *Wash*-cKO neutrophils to migrate in response to chemotactic gradients. We found that *Wash*-cKO neutrophils have defective migration, and this defect was specially marked when neutrophils were challenged with the chemokine CXCL2 or with CXCL1 (keratinocyte-derived chemokine, KC) (Fig. 6e). Although chemotaxis in neutrophils is regulated by protease-dependent cleavage of CD11b to facilitate uropod detachment, we show that the CD11b expression at the plasma membrane is normal in *Wash*-cKO neutrophils under either stimulated or resting conditions (Fig. 6f) thus ruling out a possible involvement of CD11b in the defective migration phenotype. Actin remodeling associated with spreading was also defective in *Wash*-deficient neutrophils which was manifested as reduced cycles of protrusion and retraction at the edge of lamellipodia (Supplementary Fig. 10 and Supplementary Movies 3 and 4) usually associated with spreading or motile cells^45^. Contrarily, *Wash*-cKO neutrophils formed increased filopodia as observed, for example, in Figs. 2c and 4c.

### *Wash*-cKO mice present neutrophil-mediated inflammation despite neutropenia

**Fig. 7:**
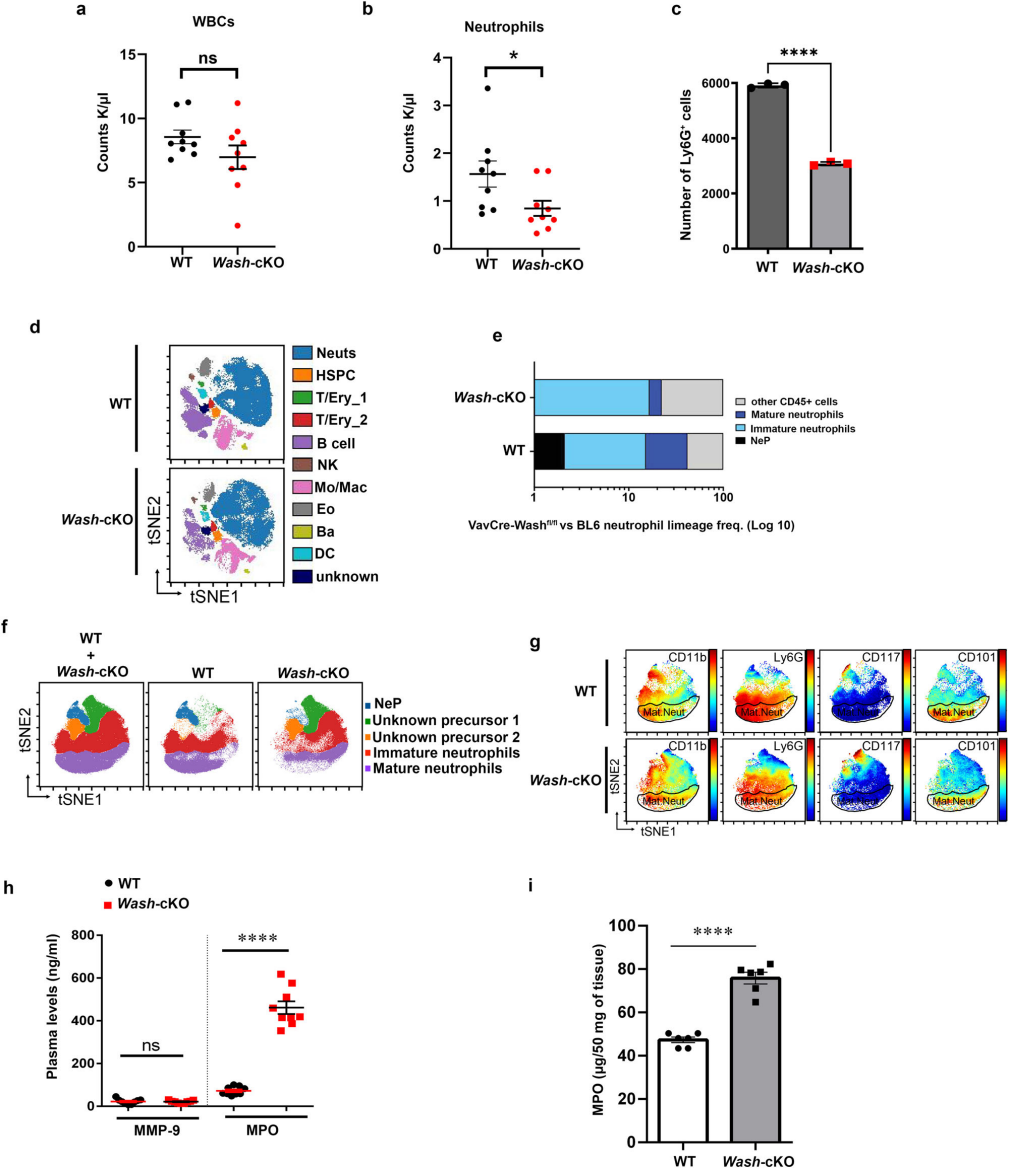
*Wash*-cKO mice present neutrophil-mediated inflammation despite neutropenia. **a**, **b** Hematologic analysis of WT and *Wash-*cKO *(Washc1*^*Δhaemo*^) mice. **a** White blood cells (WBC) counts. Mean ± SEM, *n* = 9 independent mice; ns, not significant. Two-tailed Mann-Whitney test. **b** Neutrophil counts in WT and *Wash-*cKO mice. Mean ± SEM, *n* = 9. *p = 0.0229. Two-tailed Mann-Whitney test. **c** Flow cytometry analysis of neutrophils (Ly6G+) in bone marrows from WT and *Wash*-cKO neutrophils. *n* = 3. Mean ± SEM. ****p < 0.0001. Two-tailed Student’s *t*-test. **d** CyTOF identifies similar leukocyte characteristics in WT and *Wash-*cKO mice. Bone marrow cells were stained with a panel of 43 surface markers and analyzed by CyTOF. Live CD45 + cells were selected for viSNE and FLOWSOM analysis. **e**, **f** Analysis of neutrophil lineage in wild-type and *Wash*-cKO mice. **e** Flow cytometry analysis of WT and *Wash-*cKO bone marrow neutrophil lineage maturation status. *n* = 4. **f** CyTOF analysis of wild-type and *Wash-*cKO bone marrows identifying distinctive neutrophil progenitor and precursor phenotypes. Bone marrow cells were stained with a panel of 43 surface markers and analyzed by CyTOF. Neutrophil lineage clusters from **d** were selected for viSNE and FLOWSOM analysis. *n* = 3. **g** *Wash-*cKO mature neutrophils show a similar developmental phenotype as in the WT mice. viSNE maps show neutrophil lineage clusters’ expression patterns of each CD marker as spectrum-colored dots. Blue = low expression. Red = high expression. *n* = 3. **h**, **i** *Wash-*cKO mice present neutrophil-mediated systemic inflammation. **h** Plasma levels of MMP-9 (Gelatinase granules) or MPO (myeloperoxidase, azurophilic granules) quantified by ELISA. Each symbol represents an individual mouse. *n* = 9. Mean ± SEM. ns, not significant. ****p < 0.0001. Two-tailed unpaired Student’s *t*-test. **i** Analysis of neutrophil infiltration into kidneys of wild-type and *Wash-*cKO mice measured as tissue-associated myeloperoxidase by ELISA. Each symbol represents an individual mouse. *n* = 6. Mean ± SEM, ****p < 0.0001. Two-tailed unpaired Student’s *t*-test. ns, not significant. Source data are provided as a Source Data file.

Hematologic analysis of *Wash-*cKO mice indicate that these animals present with generally lower levels of leukocytes (Fig. 7a), mainly originated by the low numbers of circulating neutrophils (Fig. 7b), a phenotype further confirmed by the reduced numbers of Ly6G^+^ cells in circulation of *Wash-*cKO mice (Fig. 7c). These mice also show reduced numbers of eosinophils (Supplementary Fig. 11). To better understand the neutropenic phenotype, we next utilized high-dimensional mass cytometry (cytometry by time of flight [CyTOF]) to study putative differences in mouse unipotent neutrophil progenitors (NeP)^46^ and neutrophil sub-populations in the bone marrow of *Wash-*cKO mice. First, we show that bone marrows from *Wash-*cKO mice show similar leukocyte characteristics to control mice of identical background (Fig. 7d). Next, studies of neutrophil-lineage frequency show reduced numbers of mature neutrophils in the bone marrow of *Wash-*cKO mice (Fig. 7e). Furthermore, bone marrow neutrophils from *Wash-*cKO mice show distinct neutrophil progenitor characteristics (Fig. 7f) suggesting that WASH regulates the rate of neutrophil development. Despite differences in the numbers of mature neutrophils, CD11b^hi^Ly6G^hi^ (mature) neutrophils of *Wash*-cKO and wild-type mice show similar profile expression of cluster of differentiation (CD) markers that indicate the maturation status on the neutrophil development hierarchy (Fig. 7g), including high expression of the immunomodulator CD101 and lack of expression of the hematopoietic cell proliferating factor c-Kit (CD117). This indicates that the neutrophil defective development phenotype in *Wash*-cKO is quantitative not qualitative. This is further supported by the observation that mature neutrophils from wild-type and *Wash-*cKO mice express similar levels of gelatinase B (Fig. 1), a granule protein that is generated late during development because tertiary granules are the last set of granules formed during neutrophil maturation^8^.

Next, to establish whether WASH-deficiency induces a pro-inflammatory phenotype in vivo, we analyzed plasma levels of neutrophil secretory proteins. In Fig. 7h, we show that despite their reduced neutrophil numbers, *Wash-*cKO mice are characterized by a marked increase in azurophilic granule cargoes in circulation. These levels of azurophilic granule cargoes in circulation were like those observed in wild-type mice after systemic pro-inflammatory insult^47^, and are therefore an indication of neutrophil-mediated inflammation. Azurophilic but not gelatinase granule cargoes were increased in the plasma of *Wash*-deficient mice, thus confirming, in vivo, the granule selectivity of the exacerbated exocytosis phenotype in *Wash-*cKO mice. Furthermore, neutrophil activation correlated with increased myeloperoxidase levels in kidney tissue (Fig. 7i) which may reflect increased secretion, further supporting that neutrophil-mediated pro-inflammatory mechanisms are activated in hematopoietic-specific WASH-deficiency. Of note MMP-9 plasma levels were not increased in *Wash-*cKO mice and pro-inflammatory cytokines were present at normal levels in plasma of unchallenged *Wash-*cKO mice (Supplementary Fig. 12) supporting that dysregulated azurophilic granule secretion in *Wash-*cKO mice is, in principle, a neutrophil-intrinsic defect. To further analyze this point, we crossed the *Wash*^fl/fl^ mice with the *Mrp8-Cre* + which expresses *Cre* recombinase under the promoter of the neutrophil-specific cargo *S100A8* (*Mrp8*). *Wash*^*fl/fl*^*/Mrp8-Cre* + mice (hereon *Wash*^ΔPMN^) were validated by genotyping and flow cytometry analysis of GFP which is bicistronically expressed with *Cre* in this model (Supplementary Fig. 13a, b). Like that shown for other *S100A8*-*Cre* models^48,49^, this approach yielded partial rather than total deletion of WASH in isolated mature (Ly6G + ) bone marrow neutrophils, as confirmed by western blotting (Supplementary Fig. 13c). Importantly, similar to *Wash*^*fl/fl*^-*Vav*-Cre + , in vivo analysis of *Wash*^ΔPMN^ mice presented dysregulated azurophilic granule secretion characterized by significantly increased levels of plasma MPO (Supplementary Fig. 13d) despite normal MMP-9 levels (Supplementary Fig. 13e). Different from the *Wash*^*fl/fl*^-*Vav*-*Cre* + , the *Wash*^*fl/fl*^-*S100A8*-*Cre* + model presented normal neutrophil numbers (Supplementary Fig. 13f). Whether this difference is caused by a secondary effect due to *Wash-*deficiency in other hematopoietic lineages in *Wash*-cKO or by the fact that WASH downregulation is only partial in the *Wash*^ΔPMN^ model, is currently unknown. Finally, isolated mature neutrophils from *Wash*^ΔPMN^ mice also presented exacerbated secretion of azurophilic granules but not gelatinase granules (Supplementary Fig. 13g, h), further supporting a neutrophil-intrinsic defect associated with dysregulated exocytosis in *Wash*-deficiency.

### WASH-deficiency contributes to the development of systemic inflammation during endotoxemia

**Fig. 8:**
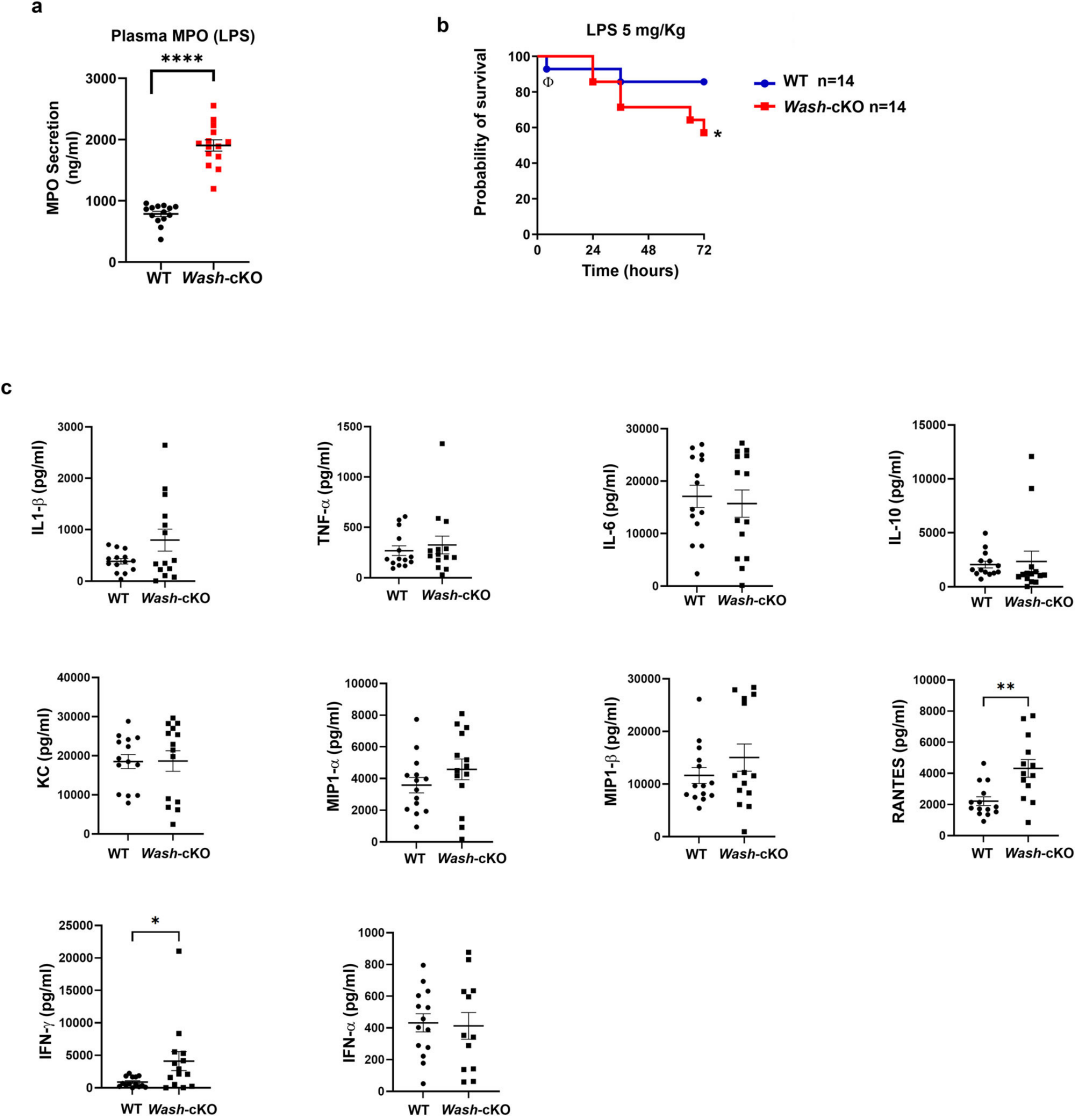
Increased neutrophil secretion and decreased survival in *Wash*-deficiency in a model of endotoxin-induced systemic inflammation. Wild-type (WT) and *Wash*-cKO mice were challenged with a single intraperitoneal injection of LPS (5 mg/Kg) (*E. coli* 0111:B4, Enzo). Blood samples were collected at 4 h after injection and mice were subsequently monitored for sickness and survival. **a** Blood samples from wild-type and *Wash*-cKO mice were spun down, and plasma was collected and analyzed for the presence of myeloperoxidase (MPO) by ELISA. A total of 14 mice for each group were analyzed in two independent experiments. Mean ± SEM. ****p < 0.0001, Two-tailed Student’s *t*-test. **b** Kaplan-Meier survival plots for wild-type (WT), and *Wash*-cKO mice after challenge with a single intraperitoneal injection of LPS (5 mg/kg). Survival curves were generated from two independent experiments with a total of 14 mice in each group. The early deceased WT mouse, Φ, denotes a statistical outlier (Grubb’s, alpha = 0.05). The difference in survival between the wild-type and *Wash*-cKO mice was significant by the log-rank test (Mantel-Cox) (*p = 0.0404). **c** Inflammatory cytokines in plasma of WT and *Wash*-cKO mice after LPS challenge (4 hours). Each symbol represents one mouse from 2 independent experiments. *n* = 14 independent mice. IL1β, TNFα, IL6, IL10, KC, MIP1α and MIP1β, IFNα, not significant. Rantes, **p = 0.0022; IFNγ *p = 0.0367, Two-tailed unpaired Student’s *t*-test. All data in **c** is represented as mean ± SEM. Source data are provided as a Source Data file.

We next studied the effect of *Wash*-deficiency in a mouse model of LPS-induced systemic inflammation. *Wash-*cKO mice treated with a single i.p. injection of LPS (5 mg/Kg), presented two to three-fold increase of azurophilic granule cargoes in circulation compared to wild-type mice, four hours after LPS insult, a time point at which the levels of this inflammatory mediator are maximal in this model (Fig. 8a). This correlated with decreased survival of *Wash*-cKO mice in response to LPS insult with 44% reported death at 72 h *vs* 14% death in wild-type mice, further supporting increased systemic inflammation in *Wash*-deficiency (Fig. 8b). This difference was observed despite no significant differences in the levels of the pro-inflammatory cytokines IL1β, IL6, IL10, MIP1-α, MIP1β and TNFα between wild-type and *Wash*-cKO mice in response to LPS challenge (Fig. 8c). However, the levels of RANTES (CCL5), a chemokine proposed to exert neutrophil recruitment^50^, and IFNγ, were elevated in *Wash*-cKO mice after LPS insult (Fig. 8c).

## Discussion

Actin remodeling regulates vesicular trafficking and exocytosis by several mechanisms. In neutrophils, we showed that actin remodeling at the azurophilic granule surface facilitate trafficking and exocytosis of the neutrophil granules with the most toxic cargoes^20^. We now show that WASH, a protein that regulates branched actin filament networks by the Arp2/3 complex^51^, localizes at neutrophil azurophilic granules where it interferes with the Rab27a-JFC1 axis and is essential for the repression of azurophilic granule exocytosis through the generation of an actin barrier at the granule membrane. Contrarily, WASH is necessary for the formation of polarized actin comets and positively regulates gelatinase granule exocytosis and their associated functions (i.e., NADPH oxidase activation). We propose that WASH contributes to the regulation of sequential exocytosis by favoring gelatinase granule secretion while maintaining azurophilic granules in a refractory state. Because WASH differentially regulates the exocytosis of neutrophil granule subtypes, the manipulation of WASH function could lead to strategies to selectively reduce neutrophil granule cargo-mediated inflammation while maintaining innate immune functions.

Azurophilic granule membranes contain a secretory machinery that favors actin depolymerization manifested at the granule surrounding areas to facilitate movement toward the plasma membrane and favor fusion^20^ but, whether the regulation of actin remodeling effectors is necessary for this mechanism is currently unknown. We previously showed that secretory organelles move in areas near the plasma membrane partially deprived of polymerized actin and that dynamic vesicles maintain an apparent actin-free environment in their surroundings^20^. However, motility is attenuated by inhibition of actin polymerization^20^, highlighting positive and negative roles for actin in granule dynamics. How actin remodeling executes this dual role both facilitating and impeding neutrophil granule motility is currently not understood. Here, we show that deficiency of the nucleation-promoting factor, WASH, influences neutrophil secretion of cargoes from different granules in different ways, inhibiting gelatinase but exacerbating azurophilic granule exocytosis.

Our data support a model where WASH-mediated actin polarization around azurophilic granules maintains this granule subset in an arrested, exocytosis-restricted, state thus preventing the secretion of the neutrophils’ most toxic cargoes. Not only does WASH obstruct azurophilic granule exocytosis by constructing an actin trap surrounding these granules, but also counteracts the azurophilic granule secretory machinery by interfering with the Rab27a-mediated recruitment of the effector JFC1, which mediates granule docking through binding to Rab27a by its amino-terminal SHD domain (synaptotagmin-homology domain) and to the plasma membrane through binding to phosphoinositol 3,4,5 triphosphate (see model in Supplementary Fig. 14)^52^. This double negative regulation on azurophilic granule secretion exerted by WASH appears to guarantee an additional level of control to ensure that the organelle with some of the most toxic, readily releasable cargoes of the organism, is maintained inactive. On the contrary, dysregulation of this mechanisms caused by the absence of WASH, induces systemic inflammation.

Although some WASP family members contain a phospholipid binding domain that mediates their recruitment to cellular membranes, WASH recruitment was proposed to be mediated by the WASH-complex protein FAM21^25^, which has been shown to harbor two basic regions that can interact with phospholipids^40^. Coincidently, in neutrophils, FAM21 puncta localized at azurophilic granules and thus it is possible that WASH recruitment to the granules is mediated by FAM21. However, because FAM21 localization at azurophilic granules decreases in WASH-deficiency, WASH may be necessary to stabilize the complex at the granule membrane.

WASH regulates actin cytoskeletal dynamics by activation of the actin nucleating activity of the Arp2/3 complex. A role for WASH-mediated Arp2/3 complex activation in the mechanism of azurophilic granule entrapment is supported by the observation that pharmacological inhibition of this complex in wild-type cells increases azurophilic granule exocytosis. However, the observation that CK666-induced azurophilic granule secretion does not reach the levels observed in WASH-deficient cells suggest that WASH plays additional, actin-independent roles in the regulation of azurophilic granule cargo exocytosis. Our data show that WASH competes with JFC1 for Rab27a binding and thus, this additional function of WASH is mediated by interfering with the azurophilic granule secretory machinery. Therefore, at the azurophilic granule, WASH performs two functions (a) it induces actin polarization and granule entrapment and (b) displaces JFC1 from Rab27a+ vesicles thus preventing JFC1 from mediating docking through PIP_3_ binding^22^.

Gelatinase but not azurophilic granule secretion is associated with WASH-dependent, polarized actin-induced trafficking but not cortical actin remodeling. This is supported by secretion data showing that treatment with cytochalasin D, which favors exocytosis by dismantling cortical actin but also impairs trafficking^20^, does not rescue the MMP-9 exocytosis defect in *Wash-*cKO cells, and by super-resolution microscopy analysis showing that actin is polarized at gelatinase granules in wild-type but not in *Wash*-cKO cells. To characterize these defects, we focused on small GTPases which are known to regulate the function of the WASP family. In particular, the Rho family GTPase, Cdc42, was demonstrated to release the autoinhibition of WASP and N-WASP by competing with the VCA (Verprolin homologous, central hydrophobic, and acidic) domain, thus exposing the binding site for Arp2/3 complex on VCA^53,54^. Similarly, WAVE proteins are regulated by the Rho GTPase Rac1 through IRSp53^55^ but, whether WASH is regulated by small GTPases is still unclear. WASH has been suggested to operate downstream of RhoA during *Drosophila* oocyte development^39^. Furthermore, Jia et al. demonstrated that WASH binds to Rac1^40^. However, the binding of Rac1 to WASH was shown to be weak and Rac1 does not activate the WASH regulatory complex^40^, suggesting that other cellular processes may be affected by this interaction. Here, we show that in the absence of WASH, neutrophils present decreased Rac1 expression, decreased recruitment of active Rac1 at gelatinase granules, and decreased granule-associated comet formation, while pharmacological activation of Rac1 increases comet size and rescues the gelatinase granule-defective secretory phenotype in *Wash*-deficiency. Our data support a mechanism where Rac1 activation facilitates the formation of actin comets and rescues exocytosis, although, as mentioned above, the contribution of additional factors to this process cannot be ruled out at this point. For instance, FAM21 presence at gelatinase granules increases in WASH-deficient cells. Park et al. demonstrated that in the absence of FAM21 excessively active actin polymerization leads to the formation of large comet tails at lysosomes leading to the conclusion that FAM21 limits or inactivates actin polymerization^56^. In agreement with these findings, here we show that in the absence of WASH, gelatinase granules fail to form actin comets which also correlates with excessive accumulation of FAM21 on these granules. Our data suggest that Rac1 activation induces comet formation and gelatinase exocytosis, but whether FAM21 is involved in this process and how actin comets may facilitate granule directional movement or secretion requires further investigation. Thus, gelatinase granules show unique requirements for WASH-mediated polarized comet formation at these secretory organelles which are not shared by azurophilic granules. We suggest a model where WASH-mediated polarized actin remodeling may facilitate the propulsion for gelatinase/specific granule trafficking to favor exocytosis of this granule subpopulation (Supplementary Fig. 14).

Defective gelatinase granule exocytosis is associated with decreased translocation of the membrane-associated subunit of the NADPH oxidase, the cytochrome *b*558, to the plasma membrane leading to impaired ROS production in *Wash*-cKO neutrophils. Here, we show that deficiency of the actin nucleator WASH is associated with impaired activation of the NADPH oxidase in response to a bacterial-derived peptide which induces the assembly of the oxidase at the plasma membrane and activates extracellular ROS. Furthermore, although PMA activates both intracellular and extracellular assembly of the oxidase, extracellular but not intracellular ROS production was defective in response to the phorbol ester, suggesting that exocytosis defects are the main cause of oxidase impairment in WASH-deficiency. However, whether WASH directly modulates the oxidase activity is currently unknown and a possible mechanism mediated by actin nucleation on the modulation of ROS production is plausible. For instance, actin filament growth is proposed to prolong the NADPH oxidase lifetime during its activation^57^ and dismantling F-actin in a cell-free system decreases oxidase activity. Independently of the mechanism, which may require further investigation, our data suggest that an increase of WASH activity may favor the activation of the oxidase while blocking azurophilic granule exocytosis, a scenario that may be beneficial under conditions in which enhancing the innate immune response without inducing inflammation may be required.

In vivo experiments show that *Wash-*cKO mice present high levels of the azurophilic granule cargo myeloperoxidase in circulation, a pro-oxidant enzyme that also has nitric oxide oxidase activity^9^ and contributes to the development of endothelial malfunction in sepsis and SIRS. Because azurophilic cargo exocytosis is not selective for the cargo, these data indicate that all azurophilic granule cargoes are elevated in the plasma of *Wash-*cKO mice including potent serine proteases like elastase, proteinase 3 and cathepsin G^58^. Contrarily, gelatinase is not detected in circulation in *Wash-*cKO mice. Azurophilic granule-mediated systemic inflammation in *Wash-*cKO mice occurs despite normal levels of pro-inflammatory mediators and normal gelatinase levels supporting that this is largely due to a neutrophil-intrinsic cellular defect, although a possible contribution of RANTES and IFNγ, which were elevated in the endotoxin-induced inflammation model in *Wash*-deficiency, cannot be ruled out at this time. The phenotype is also manifested regardless of the lower number of mature neutrophils present in the blood of *Wash-*cKO mice. An increased number of immature neutrophils are present in the bone marrow of *Wash-*cKO mice, and immature cells are frequently released to circulation in some pro-inflammatory conditions^46^, it is therefore possible that immature neutrophils contribute to the neutrophil-mediated pro-inflammatory genotype in *Wash-*cKO mice. However, a neutrophil intrinsic defect of exacerbated azurophilic granule secretion in *Wash*-deficiency is supported by three additional observations: (a) neutrophil-specific *Wash*^*fl/fl*^*/Mrp8-Cre* + mice, which are not neutrophilic, also have significantly increased plasma MPO levels, albeit not as high as those observed in *Wash*^*fl/fl*^*/Vav-Cre* + mice. (b) isolated *Wash*^ΔPMN^ neutrophils maintain the dysregulated secretory phenotype, which is also in part recapitulated in wild-type neutrophils by treatment of with an Arp2/3 inhibitor; (c) inactivation of WASH in mature human neutrophils recapitulates the secretory defective phenotypes of *Wash*-deficient neutrophils. Altogether, we propose that neutrophil intrinsic mechanisms contribute to the in vivo pro-inflammatory phenotype in *Wash*-deficiency and that this may be aided by the increased release of pro-inflammatory cytokines and immature neutrophils in the *Wash*-cKO model.

In conclusion, we have characterized the molecular mechanisms linking defective, granule-associated actin remodeling, with impaired sequential neutrophil exocytosis and have complemented these findings with data showing differentially dysregulated cargo exocytosis in vivo leading to systemic inflammation. In particular, we show that deficiency of the actin nucleator WASH is associated with decreased Rac1 and defective actin comet formation, leading to impaired gelatinase granule secretion, while increased azurophilic granule exocytosis in WASH-deficiency is caused by excessive recruitment of the Rab27a effector JFC1 and decreased entrapment of this granule subtype in polymerized actin. We postulate that genetic or pharmacological approaches to promote WASH activation could lead to translational mechanisms to reduce neutrophil-mediated inflammation caused by exacerbated secretion of azurophilic granule proteases and pro-oxidant enzymes while maintaining some of the essential neutrophil responses to infection.

## Methods

### Animals

C57BL/6 *Washc1*^*flox/flox*^*/Vav-cre*^*+*^ mice (*Wash-*cKO) and co-housed C57BL/6^*+/flox/*^*Vav-cre*^*-*^ *(WT) or* C57BL/6 controls were used in this work. The *Washc1*^*flox/flox*^ */Vav-cre*^*+*^ mouse conditional knockout model, lacking WASH expression in the haemopoietic linage (*Washc1*^*Δhaemo*^, hereon *Wash-*cKO) was generated as previously described^59^. *Wash*^*fl/fl*^*/Mrp8-Cre* + mice (hereon *Wash*^ΔPMN^) were generated by crossing the *Wash*^fl/fl^ mice with the *Mrp8-Cre* + (Tg-S100A8-cre,ires-EGFP) 1Ilw/J mice, strain #:021614, The Jackson Laboratory, which expresses *Cre* recombinase under the promoter of the neutrophil specific cargo *S100A8* (*Mrp8*), and directs bicistronic *Cre* and EGFP protein expression to CD11b + , Ly6G + , granulocytes. The characterization of this model was performed by genotyping using Transnetyx technology for the detection of both the presence of the deleted allele and the *Cre* allele, PCR for confirmation, flow cytometry and immunoblotting analysis of WASH expression in *Wash*^*fl/fl*^*/Mrp8-Cre* + mice (Supplemental Fig. 13a–c). Mice were maintained in a pathogen-free environment and had access to food and water ad libitum. Housing conditions included 12-h dark/light cycle (light 6 a.m. to 6 p.m.), ambient temperature 24 °C and humidity 50%. The mice genotypes were determined by Transnetyx and lack of WASH protein expression confirmed by Western blot. Animals were euthanized by over-anesthetization by standard and approved protocols. All animal studies were performed in compliance with the Department of Health and Human Services Guide for the Care and Use of Laboratory Animals. All studies were conducted according to National Institutes of Health and institutional guidelines and with approval of the animal review boards at The Scripps Research Institute.

### Mouse neutrophil isolation

Bone marrow cells from experimental males and females, 6 to 10-week old mice and sex and age-matched control mice were collected from the long bones of mouse legs by flashing the bones with 5 ml of PRF-RPMI using a 30 Gauge ½” hypodermic needle and a BD Luer-Lok™ 10 ml syringe. Bone marrow–derived mature neutrophils were isolated by positive selection using Anti-Ly6G MicroBead Kit or Anti-Ly6G MicroBeads UltraPure purification kit (Miltenyi Biotec, 130-120-337). The information for reagents providers and catalogue numbers is provided in Supplementary Table 2.

### Isolation of human neutrophils

Human neutrophils were isolated from normal donor’s blood by Ficoll density centrifugation as previously described^60^. All procedures regarding human subjects have been reviewed and approved by the Human Subjects Committee at The Scripps Research Institute and were conducted in accordance with the requirements set forth by the mentioned Human Subjects Committee and in accordance to NIH guidelines. Informed consent was provided by the donors.

### Western blotting

Proteins were separated by gel electrophoresis using BOLT^TM^ gels and 3-(*N*-morpholino) propanesulfonic acid buffer (Life Technologies, NW04120BOX). Proteins were transferred onto nitrocellulose membranes for 60 min at 100 volts, at 4 °C. The membranes were blocked with tris-buffered saline (TBS) containing 5% (wt/vol) blotting-grade nonfat dry milk blocker (Rockland, Limerick, PA) and 0.1% (wt/vol) Tween 20. Proteins were detected by probing the membranes with the indicated primary antibodies at appropriate dilutions and using a detection system consisting of horseradish peroxidase–conjugated secondary antibodies (Bio-Rad Laboratories, Hercules, CA) and the chemiluminescence substrates SuperSignal, WestPico, and WestFemto (Thermo Scientific) and then visualized using the c600 Azure Biosystem or by film development. All original blots are shown as either source data or in Supplementary Fig. 15.

### Immunofluorescence microscopy analysis

Neutrophils were seeded on untreated coverglasses using 4-Chamber 35 mm dish with 20 mm microwells (Cellvis LLC Cat # D35C4-20-1.5-N) and incubated at 37 °C for 30 min, then fixed with 3.7% paraformaldehyde for 8 min, permeabilized with 0.01% saponin, and blocked with 1% BSA in PBS. The samples were labeled with the indicated primary antibodies overnight at 4 °C in the presence of 0.01% saponin and 1% BSA. Samples were washed and subsequently incubated with appropriate secondary antibodies for 2 hours at room temperature. The cells were then stained with DAPI and mounted with Fluormount G. Where indicated, F-actin was labelled with 5 µl of a 6.6 µM concentration of Alexa 488- or Alexa 647-Phalloidin in 200 µl PBS with 0.05% Triton X-100. Samples were analyzed with a Zeiss LSM 880 laser scanning confocal microscope attached to a Zeiss Observer Z1 microscope at 21 °C, using a 63× oil Plan Apo, 1.4 numerical aperture (NA) objective. Where indicated, the images were collected using enhanced resolution microscopy (Airyscan) which generates images with substantially increased SNR (signal-to-noise ratio)^61^. Images were collected and fluorescence intensity and colocalization quantified using ZEN-LSM software. Actin colocalization at the edges of granules was analyzed using Manders’ correlation coefficient as described below. The images were processed using ImageJ.

### Images acquisition and colocalization analysis

All 8-bit images were acquired using the full dynamic intensity range (0–256) of the specified fluorophores. Stacks (on average 20 slices) were acquired with Nyquist resolution parameters using a 0.3 µm step size and optimal frame size of 1932 × 1932. Images were processed for quantitative colocalization in either Zen Pro in 2D (Zeiss) or Image Pro Premier 10 (IPP10) (Media Cybernetics). Briefly, maximum intensity projections (MIP) were generated in Zen then processed using the Zen or IPP10 colocalization modules. Samples stained for secondary antibodies and unlabeled controls alone were used to define thresholds of real signal above background and non-specific signal, which were typically and conservatively set at (50–80 to 256). In Zen or IPP10, regions of interest (ROI)s were drawn around each individual cell, previously defined minimum thresholds were input, two fluorescent channels signals are selected and the software automatically calculates pixel intensity spatial overlap coefficients between them (both Mander’s and Pearsons are scored). Mander’s overlap coefficient (MOC) was primarily used and is described as MOC = ∑i(Ri × Gi)∑iRi2 x ∑iGi2 where Ri is the intensity of the first fluorophore in an individual pixel, whereas Gi is the corresponding intensity for the second fluorophore in the same pixel. Where indicated areas of overlap were pseudo-colored in white (binary) to mathematically and spatially defined colocalized zones in cells.

### Quantification of F-actin proximity to neutrophil granules

To establish granular entrapment in F-actin, we analyzed F-actin adjacency to neutrophil granules. To this end we utilized Spot to Surface Distance Transformation (SSDT) module to define the granular centroid location and automatically calculate its distance to the most proximal actin fiber in three-dimensional space. Specifically, within the Imaris software, we utilized the three-dimensional SSDT module to define and mark the centroid of all MPO fluorescent positive bright regions of interest (ROI) based on their voxel space and was defined as a spot. Each spot of a defined intensity and voxel volume was associated with the most proximal F-actin fiber which was iso-surfaced in Imaris. The SSDT module scored the proximity for each pair of MPO spots and iso-surfaced phalloidin F-actin fiber. The SSDT module scored the proximity for each pair of MPO spots and phalloidin puncta. The data were binned in the nm scale at predefined distance intervals and plotted using Prism.

### Transfection of mouse neutrophils

Mouse neutrophil nucleofection was carried out using the 4D-Nucleofector X Unit system (Lonza) following the manufacturer’s instructions. In brief, mouse neutrophils were counted, and 1 × 10^6^ cells were resuspended in 20 μl of Lonza P3 solution with 1 μg of DNA and the solutions were transferred into the X Unit and then subjected to nucleofection in the 4D-Nucleofector using program EA-100. The cells were then resuspended in phenol red–free RPMI (Life Technologies) and seeded into four-chamber 35 mm glass-bottom dishes (No. 1.5 borosilicate coverglass). The cells were incubated at 37 °C in a tissue culture incubator (5% CO_2_), and used in fluorescence-based assays 3–4 h after transfection.

### Total Internal Reflection Fluorescence (TIRF) Microscopy

TIRFM experiments were performed using a 100×/1.45 NA TIRF objective (Nikon Instruments, Melville, NY) on a Nikon TE2000U microscope custom modified with a TIRF illumination module as described^20^. Images were acquired on a 14-bit, cooled charge-coupled device (CCD) camera (Hamamatsu) controlled through NIS-Elements software. After placing the cells on the stage, the position of the individual laser beams was adjusted with the TIRF illuminator to impinge on the coverslip at an angle to yield a calculated evanescent field depth of a 100 or 400 nm for TIRFM and pseudo-TIRFM modes, respectively. For TIRFM, the objective is pre-calibrated for Z-position assignment and for chromatic shift between the channels using 100 nm Tetraspeck beads as specified by Nikon. For pseudo-TIRFM, the calculation of the penetration depth was performed by measuring the position at which the beam is exiting from the center of the objective and subsequently correlated to the penetration depth *vs* the displacement of the beam from the center using the exponential decay depth equation described in ref. 62 and incorporated in the Nikon software. The penetration depth for different wavelengths is estimated plus or minus 20 nm for a given beam position. For live experiments, the images were recorded using 200–300 ms exposures depending on the fluorescence intensity of the sample. Vesicles and actin dynamics was tracked using surface rendering or spots modules in IMARIS.

### Stochastic Optical Reconstruction Microscopy (STORM) Imaging

STORM analysis was performed as we described previously^35^. Briefly, super-resolution microscopy using the Nikon STORM system is based on the stochastic optical reconstruction microscopy (STORM) technology that was developed by Xiaowei Zhuang’s laboratory at Harvard University^63^. In this method, the position of individual molecules is localized with high accuracy by switching them on and off sequentially using appropriate laser power settings and buffer conditions. For neutrophil experiments, cells seeded in glass bottom dishes (#1.5 borosilicate cover glass) are fixed with freshly made 4% paraformaldehyde for 10 min at room temperature, permeabilized, and blocked for 1 h with 1% BSA in PBS. The cells are labeled with primary antibodies in blocking solution for 2 h at room temperature, washed, and then incubated with the following secondary antibodies: For two-color STORM, Alexa-647- and Atto-488 or Alexa-488-conjugated secondary antibodies are used to label the proteins of interest^35^. For three-color super-resolution imaging, Alexa-568-conjugated Phalloidin was used as a third channel in addition to the above two sets of secondary antibodies. The samples were washed, post-fixed with freshly made 4% paraformaldehyde, and re-washed with water. Prior to STORM imaging, the samples were incubated in freshly prepared STORM buffer (50 mM Tris buffer (pH 8.0), 10 mM NaCl, 10% glucose, 0.1 M mercaptoethanolamine, 56 units/ml glucose oxidase, and 340 units/mL catalase). The STORM buffer is prepared by adding 100 μl of MEA solution and 10 μl of GLOX solution to 890 μl of buffer B (50 mM Tris buffer (pH 8.0), 10 mM NaCl, 10% Glucose). The samples were placed on the microscope stage, and then imaged using a 100 × 1.49 NA Apo TIRF objective either with or without TIRF illumination. Images were collected at a frame rate of about 15 ms on 256 × 256 pixel region of the EMCCD camera using the multicolor sequential mode setting of the Nikon STORM module in Elements software^35^. Three-dimensional (3D) STORM images are generated by introducing a cylindrical lens in the light path, which enables the assignment of Z position based on the shape of the point spread function. Before STORM imaging, the objective is pre-calibrated for Z position assignment and for chromatic shift between the channels using 100-nm Tetraspeck beads as specified by Nikon. After initially photo bleaching the samples using high laser power, the power on the lasers is adjusted, so that between 50 and 300 molecules can be mapped per frame for each channel. Acquisition is stopped after a sufficient number of frames are collected (yielding 1-2 million molecules), and the super-resolution images are reconstructed with the Nikon STORM software. Automatic correction for lateral and axial drift was performed by using an autocorrelation method. Axial drift over the course of the acquisition is also minimized by engaging the Nikon perfect focus system. The position of well-separated molecules is estimated from the diffraction-limited images produced by the software and the precision of the localization during a switching cycle from the photon count of the individual switching event. Mean localization accuracy of about 20 nm is in good agreement with those reported previously, which was also confirmed using Alexa-488, −568, and −647 labeled IgG molecules that had been spread on coverslips at a low density. A Gaussian fit is used by the software to localize the position of each event to the final super-resolution image.

### Three-dimensional STORM Data analysis

Images obtained on the Nikon N-storm (Nikon Inc) system were converted to high resolution images, fully calibrated, and imported into Imaris (Bitplane Inc) where they were analyzed using two well established modules: Spots, to mark the centroid location, and Colocalized Spots, to mark and score paired spots that lie within a defined distance from each other in three-dimensional space as previously described^64^. The spots created are analyzed using the Colocalized Spots module, to mark and score paired spots that lie within a defined distance from each other in three-dimensional space. Specifically, the imported localization coordinate map of all fluorescent N-storm confirmed blinks, which are previously filtered for drift and background signals in the NIKON software, are represented as spheres in IMARIS where the diameter of the sphere represents the localization accuracy, and their centroid is used to compare distances between same and different paired molecules. For comet analysis, single molecular clusters obtained from all fluorescent N-storm confirmed blinks, were imported and a localization coordinate map built, where the centroid of the sphere is the central coordinate position in three-dimensional space, and the diameter of the sphere (spot) is the localization accuracy error. Actin spots that lie within a defined distance (<50 nm) from the MMP-9 signal (green) were considered in contact with the granule marker (red) and distant spots that form the comets were represented in pale red.

### STED super resolution microscopy

Neutrophils (200,000) were seeded in a 4-quadrant dish for 30 min at 37 °C, fixed and permeabilized using 400 μL of 0.3% glutaraldehyde and 0.25% Triton X-100 in Cytoskeletal Buffer (10 mM MES, pH 6.1, 150 mM NaCl, 5 mM EGTA, 5 mM glucose and 5 mM MgCl_2_) for 2 min. This was followed by a second fixation step using 400 μl of 2% glutaraldehyde in Cytoskeletal Buffer for 10 min. The samples were treated with 1 ml 0.1% NaBH4 (freshly prepared in PBS) for 7 min to reduce background fluorescence and washed with PBS for three times, each time allowing for 10 min incubation. The cells were permeabilized with PBS containing 1% BSA and 0.2% TX-100 for 15 min at room temperature, subsequently blocked with PBS with 1% BSA for 1 hour at room temperature and incubate with anti-mouse MPO primary antibody (Hycult) overnight at 4 °C. The samples were stained with STAR-red secondary antibody and Alexa-594-phalloidin. The samples were analyzed, and images acquired using an Abberior STEDYCON STED super resolution microscope.

### Super-resolution radial fluctuations (SRRF)

Super resolution images of neutrophil granules near the adherent cell surface were obtained by processing TIRF microscopy images using the SRRF method^65^. Neutrophils were seeded on 8-well chamber slides with #1.5 coverglass bottoms, fixed, and immunostained for MMP-9 and MPO with an AlexaFluor-488 or AlexaFluor-647 secondary antibodies, as above. When indicated, cells had been treated with cytochalasin D. Images were acquired on the Oxford Nanoimager (Oxford Nanoimaging) with a 63x/1.49 NA oil objective and a 117 nm pixel size. The 473 nm excitation laser was set at the critical angle to achieve TIRF illumination. For each image, 50 frames were acquired. These image sets were processed with the FIJI plugin SRRF^66^ using default settings, resulting in a single image with improved resolution (23.4 nm pixel size), in which individual granules could be visualized. At least 5 fields-of-view were acquired for each condition in 3 separate experiments.

### Neutrophil stimulation, ELISA, cytokine analysis and flow cytometry analysis

Mouse neutrophils (1 × 10^6^) isolated from the bone marrow of WT and *Wash*-cKO mice, were resuspended in phenol-red free RPMI and either treated with GM‐CSF (10 ng/ml), LPS (100 ng/ml), cytochalasin D (1 µg/ml) or left untreated for 30 min at 37 °C, at which point the cells were stimulated with 10 µM fMLF for 10 min. Where indicated, cells were stimulated with 100 ng/ml PMA for 30 min at 37 °C. Also, where specified, the cells were treated for 1 hour with a RhoA inhibitor (cell-permeable C3 Transferase, 2 µg/ml, Cytoskeleton, CT04), a Rac1 activator (CID888706, 10 µM), the Arp2/3 inhibitor CK666 (50 µM or 150 µM) or the Rab27a-JFC1 binding-inhibitor, Nexinhib20 (10 µM) before stimulation. The cells were spun down and the supernatants were collected for ELISA analysis. MMP-9 ELISA was performed using the R&D mouse MMP-9 ELISA duo kit. MPO ELISA was performed using the R&D mouse MPO ELISA duo kit. Mice cytokines were analyzed using multiplex technology (Millipore). To evaluate the plasma membrane expression of CD11b or CD63, the cells were blocked in PBS with 1% BSA for 10 min and stained with anti–mouse CD11b or CD63-Alexa 647 (clone M1/70; BD Biosciences, San Jose, CA). Ly6G staining was used to gate the neutrophil population using anti–mouse-Ly6G-fluorescein isothiocyanate (clone 1A8; BD Biosciences). The cells were then washed, fixed with 1.5% paraformaldehyde and the samples were analyzed using the NovoCyte 3000 flow cytometer with BD FACS Diva 6 software, and the data were processed using FlowJo (Ashland, OR) software.

### TR-FRET protein binding assays

TR-FRET reactions were performed as described before^33^. Briefly, cell lysates, obtained using nitrogen cavitation to preserve the integrity of organelles, expressing Myc-JFC1 or EGFP-Rab27a and mCherry-WASH were mixed and incubated with a terbium-conjugated anti-Myc antibody for 10 minutes. The samples were excited at 340 nm. The emission peak of terbium (centered at 490 nm) overlaps with the excitation spectrum of GFP. FRET signal is measured by detecting GFP emission at 520 nm, and results are expressed as the emission ratio of the acceptor (GFP, 520 nm)/donor (terbium, 490 nm, used as internal control). An increased emission ratio is indicative of specific binding.

### Mass cytometry analysis (CyTOF) of mouse neutrophils

BM cells were harvested from femurs, and tibias of 6-10 week old mice as previously described^67^. Briefly, bones were centrifuged for the collection of marrow into ice cold D-PBS (Gibco) with 2 mM EDTA to prevent cation-dependent cell-cell adhesion. Prior to staining cells, cells were subject to a red blood cell lysis (RBC lysis buffer, eBiosciences) at room temperature. Cells were washed and filtered through a 70 μm strainer. Cell suspensions were prepared by sieving and gentle pipetting to reach final concentration of 3 × 10^6^ cells per 100 µl buffer.

Metal-conjugated antibodies were purchased directly from Fluidigm for available targets. For all other targets, antibody conjugations were prepared using the Maxpar Antibody Labeling Kit according to the recommended protocol provided by Fluidigm. All antibodies used in CyTOF are described in Supplementary Table 3 and their working dilutions in Supplementary Table 4. Maxpar-conjugated antibodies were stored in PBS-based antibody stabilization solution (Candor Biosciences) supplemented with 0.05% NaN3 at 4 °C. All antibodies were titrated before use.

Flow cytometry staining was performed in FACS buffer (D-PBS + 1% BSA + 0.1% sodium azide + 2 mM EDTA) on ice. Prior to surface staining, anti-CD16/32 (FITC) antibody was added for 15 min to stain and block the Fc receptors. Surface staining was performed for 30 minutes in a final volume of 100 µl. Cells were washed twice in at least 200 µl FACS buffer before acquisition. Results were acquired using an LSRII or an LSR Fortessa (BD Biosciences). All flow cytometry was performed on live cells. Calculations of percentages of CD45^+^ immune cells were based on live cells as determined by forward and side scatter and viability analysis. All analyses were repeated at least 3 times, and purity of sorted fractions was checked visually and by reanalysis of the surface markers. Data were analyzed using FlowJo (version 10.1r5). A figure exemplifying the gating strategy can be found in the Supplementary Information (Supplementary Fig. 16).

CyTOF was performed following previously described protocols^67,68^. Briefly, 5 μM Cisplatin (Fluidigm) were used to determine viability. Prior to surface staining, RBC-lysed WB cells were resuspended in staining buffer for 15 minutes on ice to block Fc receptors. The surface antibody cocktail was added into cell suspensions for 1 hour at 4 °C. The cells were then washed with staining buffer and fixed with 1.6% paraformaldehyde (Thermo Fisher) for 15 minutes at RT. Afterwards, 1 ml of intercalation solution for each sample was prepared by adding Cell-ID Intercalator-Ir (Fluidigm) into Maxpar Fix and Perm Buffer (Fluidigm) to a final concentration of 125 nM (a 1000X dilution of the 125 μM stock solution) and vortex to mix. After fixation, the cells were resuspended with the intercalation solution and incubated overnight at 4 °C. Cells were then washed in staining buffer, and then with subsequent washes in Cell Acquisition Solution (CAS) (Fluidigm), to remove buffer salts. Next, the cells were resuspended in CAS with a 1:10 dilution of EQ Four Element Calibration beads (Fluidigm) and filtered through a 35 μm nylon mesh filter cap (Corning, Falcon). Samples were analyzed on a Helios 2 CyTOF Mass Cytometer (Fluidigm) equipped with a Super Sampler (Victorian Airship & Scientific Apparatus) at an event rate ≤ 500 events/second. Mass cytometry data files were normalized using the bead-based Normalizer^69^ and analyzed using Cytobank analysis software (https://www.cytobank.org/).

### Stimulated secretion in SLO‐permeabilized human neutrophils

Secretory assays using SLO‐permeabilized cells were performed as previously described^19^. Briefly, neutrophils were washed twice with PBS and resuspended in permeabilization medium. Human neutrophils (2.5 × 10^6^) were resuspended in permeabilization buffer (100 μl) and transferred to a tube containing 2 μl of 2500 units/mL SLO in the presence of the indicated antibodies (50 μg/ml) and incubated at 37 °C for 5 min. Where indicated, the cells were stimulated with fMLP (1 μM) for additional 15 min. The reactions were stopped by transferring the samples to ice and immediately centrifuged at 16,000 × *g* at 4 °C for 5 min. Supernatants were stored at −20 °C until the assays were performed. MMP9 in the condition medium was analyzed by immunoblotting. Plasma membrane upregulation of CD63 (LAMP3) was analyzed by flow cytometry. The anti-mWASH (360–472) antibody used in this assay was described previously (25).

### Pulldown assay

Cells (293 T cells, ATCC, CRL-3216) were transfected with mCherry-WASH1-C-18 construct for 48 hours, and then lysed using lysis buffer (20 mM Tris pH: 8, 137 mM NaCl+ 0.5% Triton X-100), the samples were cleared by centrifugation and the supernatants were collected. Glutathione Sepharose 4B beads were incubated with 100 pmol of GST-Rab27a protein for 75 min at 4 °C, followed by 3 washes with PBS. The conjugated protein-bead mixture was then incubated with the cell lysates, overnight, at 4 °C. Following three washes with wash buffer (PBS, 0.05% Tween-20), the beads were resuspended in NuPAGE sample buffer, boiled and the pulldown samples were subjected to Western blotting.

### Expression vectors

The mCherry-WASH1-C-18 construct was obtained from Addgene. GST-Rab27a, YFP-actin and GFP-LAMP3 were described previously^19,70^.

### Antibodies and staining reagents

Primary antibodies used for Western blotting (WB), immunofluorescence (IF) staining and flow cytometry (FC) were as follows with dilutions inticated in parenthesis: MPO, clone 8F4 (HyCult) (IF: 1:200); MPO, AF3667, R&D (1:400); MMP9, AF909, R&D (WB 1:1000); the anti-WASH (WB 1:1000) and anti-FAM21 (IF 1:200) antibodies were described before^25,28^. The anti-CD63 (clone NVG-2), anti-CD11b (clone M1/70) and anti-Ly6G (clone 1A8, 127610) (flow cytometry) antibodies were from Biolegend (FC 1:50). The anti-neutrophil elastase, Ab68672, was from Abcam (FC 1:50) and phalloidin was from Thermo Fischer. The anti-Rac1 antibody was from Proteintech (24072-1-AP) (WB 1:1000) and the anti-Rac1-GTP from NewEast Biosciences (26903) (IF 1:100); anti-RhoA was from Santa Cruz Biotechnology (SC-418) (WB 1:1000; IF 1:100), anti-Rab21 from Novus Biologicals (NBP1-81544) (IF 1:200) and anti-Arp2 (ab49674) (IF 1:200) was from Abcam. Myc-Tag (9B11) Mouse mAb #2276 (WB:1:1000). Anti-mCherry antibody Abcam, [EPR20579] (ab213511), WB, 1:1000.

### Transmission electron microscopy

Cells were fixed using 4% paraformaldehyde in 0.1 M phosphate buffer, pH 7.4, overnight at 4 °C. Fixed cells were washed with 0.15 M glycine/phosphate buffer, embedded in 10% gelatin/phosphate buffer, and infused with 2.3 M sucrose/phosphate buffer overnight at 4 °C. Frozen sections of 80–90 nm were placed onto Formvar- and carbon-coated copper grids. Grids were placed on 2% gelatin at 37 °C for 20 min and rinsed with 0.15 M glycine/PBS, and the sections were blocked using 1% cold-water fish-skin gelatin. Grids were viewed using FEI Tecnai™ Spirit transmission electron microscope.

### Analysis of reactive oxygen species production

ROS Production, measured by the luminol (intracellular) or isoluminol (extracellular)-dependent chemiluminescence assays, was carried out as previously described^68,71^. Briefly, 1 × 10^6^ neutrophils resuspended in RPMI medium in a final volume of 50 µl were placed in a 384-well microtiter plate, warmed to 37 °C, and luminol was added to reach a final concentration of 100 µM (DMSO final concentration was 0.1%). The cells were stimulated with fMLF (10 µM), PMA (0.1 µg/ml). Chemiluminescence was continuously monitored using an Envision 2105 reader (PerkinElmer).

### Superoxide anion production

Superoxide anion production was continuously monitored using the SOD-inhibitable cytochrome *c* reduction assay at 37 °C^60^. Neutrophils (1 × 10^6^) were washed twice with phosphate-buffered saline (PBS) and resuspended in HBSS with Ca^2+^ and Mg^2+^ The cells were stimulated with phorbol ester (0.1 µg/ml) or fMLF (10 µM). Superoxide anion production was continuously monitored using the SOD-inhibitable cytochrome *c* reduction assay at 37 °C as described previously^60^.

### Neutrophil migration assay

Neutrophil migration assays were performed using uncoated Trans-well multiwell-plates with permeable polycarbonate membrane inserts with a pore size of 3 µm (Thermo Fisher, Catalog No.07-200-148). Purified 1 × 10^6^ mouse bone-marrow neutrophils resuspended in RPMI containing 0.5% BSA were applied onto the upper chamber. The indicated chemoattractants (CXCL1 or CXCL2) were added to the lower chambers in a volume of 500 µl at a concentration of 50 ng/mL and cells were incubated for 1 h at 37 °C in a tissue culture incubator. The cells that migrated to the bottom chamber were fixed using 2% PFA and quantified by flow cytometry.

### LC-MS/MS analysis

Proteomics analysis was performed at the Scripps Research Institute proteomics core facility. Briefly, proteins were solubilized in 0.2% Rapigest (Waters Corportation) and reduced in 5 mM D,L-dithiothrietol (Sigma) for 30 minutes followed by alkylation with 15 mM iodoacetamide (Sigma) for 30 minutes in the dark. Proteins were digested with trypsin at 37 °C using a 1:30 (w/w) enzyme to substrate ratio. Peptides were analyzed by reverse-phase chromatography before mass spectrometry analysis using the following method. Nanoelectrospray capillary column tips were made in-house by using a P-2000 laser puller (Sutter Instruments). The columns were packed with Zorbax SB-C18 stationary phase (Agilent) purchased in bulk (5-Micron particles, with a 15-cm length and a 75-mm inner diameter). The reverse-phase gradient separation was performed by using water and acetonitrile (0.1% formic acid) as the mobile phases. The gradient consisted of 5% acetonitrile for 10 min followed by a gradient to 8% acetonitrile for 5 min, 35% acetonitrile for 113 min, 55% acetonitrile for 12 min, 95% acetonitrile for 15 min, and re-equilibrated with 5% acetonitrile for 15 min. Data-dependent MS/MS data were obtained with an LTQ linear ion trap mass spectrometer using a home-built nanoelectrospray source at 2 kV at the tip. One MS spectrum was followed by 3 MS/MS scans on the most abundant ions after the application of the dynamic exclusion list. All MS/MS data samples were searched against the NCBI Mus musculus (house mouse) database using Mascot (version 2.3.02; Matrix Science, London, United Kingdom). Mascot searches were conducted using a peptide mass tolerance of 2.0 Da, a fragment ion mass tolerance of 0.80 Da, fixed modifications of carbamidomethylation (C), variable modifications of oxidation (M), an enzyme of trypsin and a maximum of one missed cleavage. Proteins with a *P*  <  0.05 (corresponding to a Mascot ion score greater than 45) were identified with two or more peptides and considered at the 95% confidence level.

### Statistical analysis and reproducibility

Data are presented as mean, and error bars correspond to standard errors of the means (SEM) unless otherwise indicated. Statistical significance was determined using the unpaired Student’s *t-*test, nor parametric Mann–Whitney test, one-way ANOVA Tukey’s multiple comparisons test or Kruskal-Wallis test, using GraphPad InStat (version 3) or Excel software, or Wilcoxon signed rank test, and graphs were made using GraphPad Prism (version 4) software. Each method used is specified in its corresponding figure legend. All measurements were taken from distinct samples. All methods were analyzed using two-tailed analyses unless indicated. For hematological, cytokine and some MPO ELISA studies, the investigators were blinded to group allocation. A *p* value < 0.05 was considered statistically significant. Assays were repeated at least three times including those shown as representative data unless otherwise stated in the figure legend. The number of samples, cells or mice per group is indicated in Figure Legends. Our research includes predetermination of sample size based on power analysis, blinding of experimentalists to genotype and vehicle or drug when possible; data reporting including all animals; randomization of littermates to the drug or placebo groups; and reporting of positive and negative results.

### Reagents and resource sharing

Information and reasonable requests for resources and reagents should be directed to and will be fulfilled by the lead contact, Sergio D. Catz (scatz@scripps.edu)

### Reporting summary

Further information on research design is available in the Nature Research Reporting Summary linked to this article.

## Supplementary information

Supplementary Information

Description of Additional Supplementary Files

Supplementary Data 1

Supplementary Data 2

Supplementary Movie 1

Supplementary Movie 2

Supplementary Movie 3

Supplementary Movie 4

Reporting Summary

## Data Availability

Information on antibodies used is provided in the antibody list in Supplementary Tables 2-4 and in the Methods section. Uncropped gels are provided as Source Data and in the Supplementary Information file. The proteomics data generated in this are available via ProteomeXchange with identifier PXD036270, and also included in Supplemetary Data 1 and 2. The immunoblotting data generated in this study are provided in the Supplementary Information and Source Data files. The statistical raw data are provided in the Source data file. Source data are provided with this paper. All data that support the findings of this study are available within the article, its Supplementary Information, or from the corresponding author upon reasonable request. NCBI Mus musculus (house mouse) database accessible link: https://www.ncbi.nlm.nih.gov/protein?term=(Mus+musculus+genome)+AND+%22Mus+musculus%22%5Bporgn%5D&cmd=DetailsSearch. Source data are provided with this paper.

## Source data

Source Data
