# Supplementary material for: Differential dysregulation of granule subsets in WASH-deficient neutrophil leukocytes resulting in inflammation
Source: Nat Commun. 2022 Sep 21;13:5529. doi: 10.1038/s41467-022-33230-y (PMC9492659; doi:10.1038/s41467-022-33230-y)
Supplement: Supplementary file 1 — Supplementary Information [file 41467_2022_33230_MOESM1_ESM.pdf]

Differential dysregulation of granule subsets in WASH-deficient neutrophil leukocytes resulting in inflammation

Johnson et al. Supplementary Information

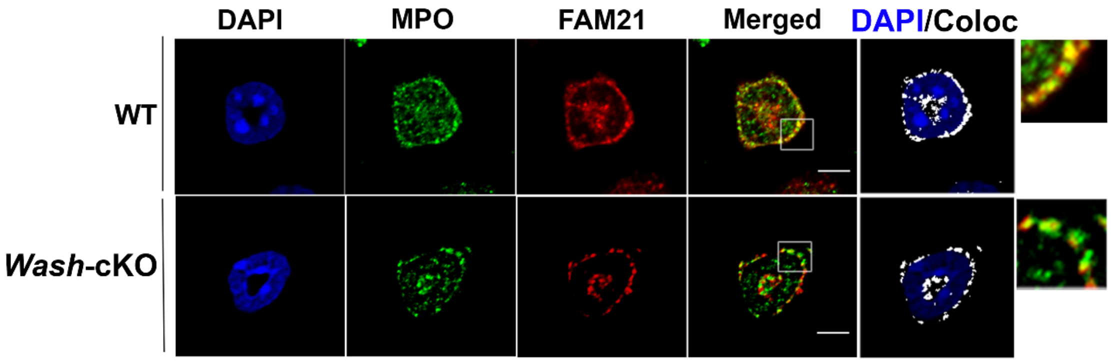

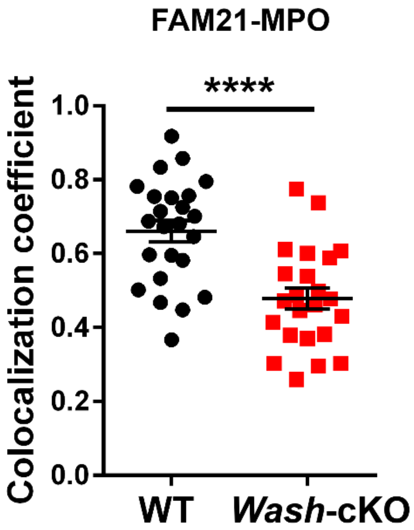

Supplementary Figure 1
FAM21 localizes at azurophilic granules
Immunofluorescence analysis of the colocalization of endogenous FAM21 at MPO-positive puncta in WT and *Wash*-cKO *Washc1*^{haemo} neutrophils. Colocalization shown in white. Lower panel, Colocalization coefficient, mean ± SEM. 24 WT and 23 *Wash*-cKO analyzed from 3 independent mice. Scale bar = 3 μm. ****, p<0.0001. Two-tailed unpaired Student's t-test.

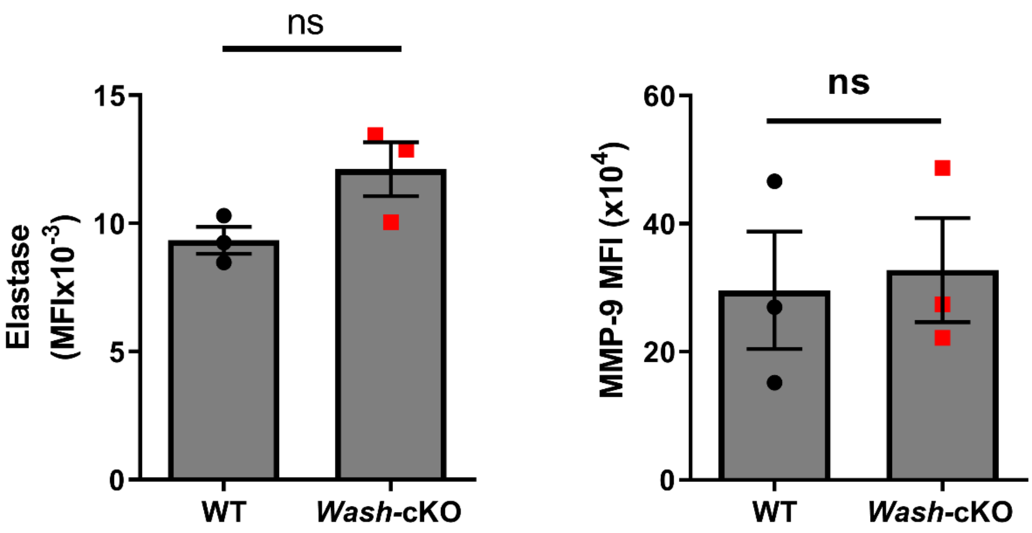

Supplementary Figure 2
Wild type and *Wash*-cKO *Washc1*^{haemo} neutrophils present similar levels of expression of azurophilic granule cargoes and gelatinase granule cargoes
Flow cytometry analysis of total elastase and MMP-9 expression in permeabilized cells. n=3 independent mice. Mean ± SD. ns, not significant. Two-tailed Mann-Whitney test.

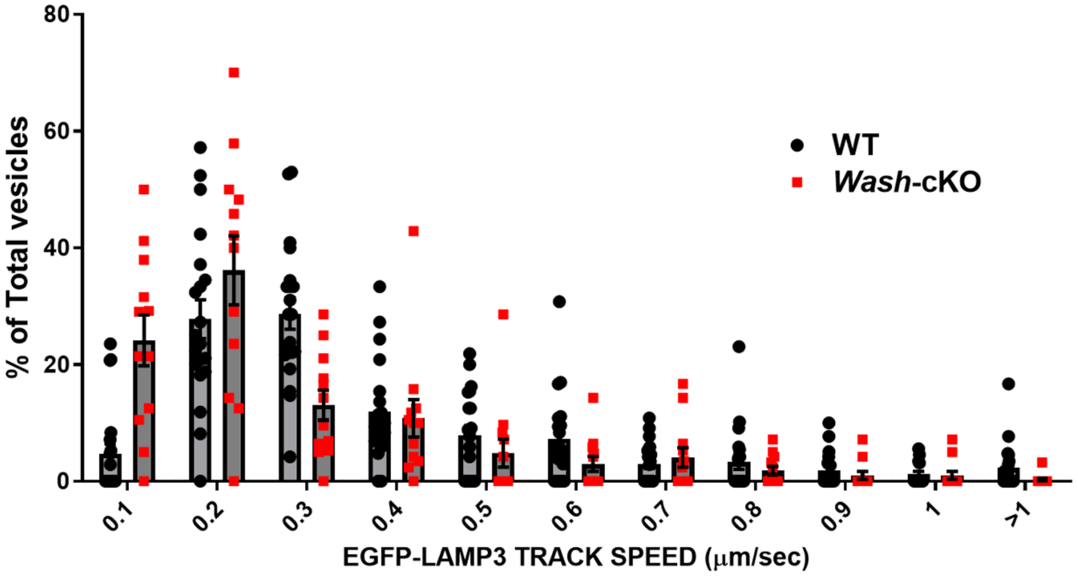

Supplementary Figure 3

Analysis of the vesicular dynamics of neutrophil granules expressing EGFP-LAMP3, analyzed at the exocytosis active zone using TIRF microscopy

Analysis complementary to Figure 2i. Analysis of vesicular dynamics of neutrophil granules expressing EGFP-LAMP3, analyzed at the exocytosis active zone using TIRF microscopy. The speeds for the independent vesicles were binned in 0.1 µm/s increments and plotted as a percentage of total vesicles for a given cell. Results are represented as mean ± SEM from 20 WT cells and 12 *Wash*-cKO cells from two independent experiments displaying numbers of non-motile (docked) and motile azurophilic granules in wild-type and *Wash*-cKO cells.

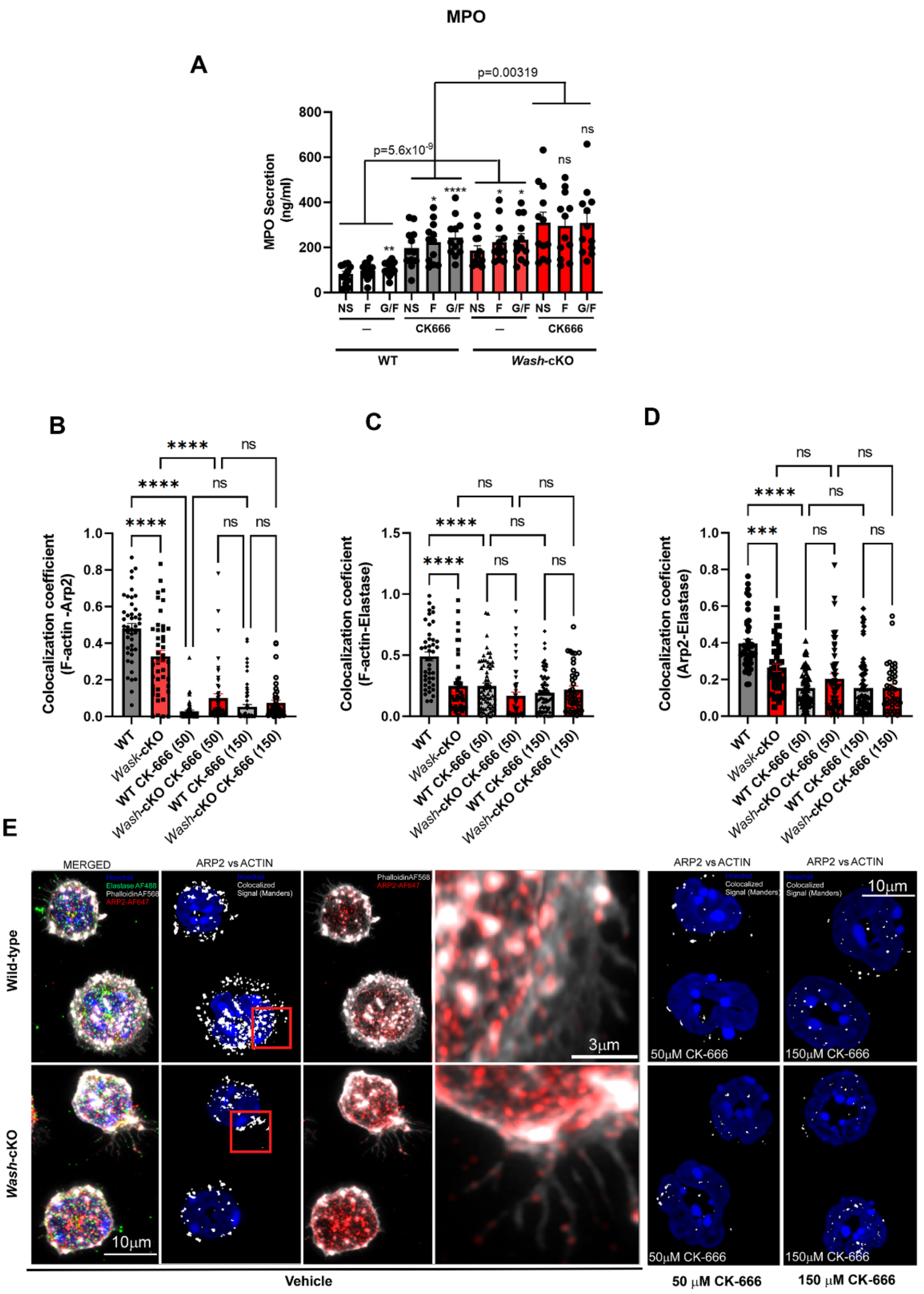

Supplementary Figure 4

Effect of the Arp2/3 inhibitor CK666 on azurophilic granule exocytosis

A, Wild-type and *Wash*-cKO (*Washc1$^{\Delta haemo}$*) neutrophils were treated with CK666 (50μM) or vehicle for 1 hour and subsequently stimulated with fMLF (F). Where indicated, the cells were primed with GM-CSF before stimulation (G/F). n=12 independent mice analyzed in four independent experiments. Mean ± SEM. The compared groups are indicated with brackets in the

figure. Two-tailed paired t-test across samples and conditions (NS, F, and G/F). The analysis to compare unstimulated and stimulated conditions within each group were performed by one-tailed paired t-test and indicated with asterisks; *, $p<0.05$; **, $p<0.01$; ****, $p<0.0001$; ns, not significant. B-D, Analysis of the effect of CK666 on the localization of Arp2 and azurophilic granule (elastase) related to F-actin (phalloidin). Cells were treated with 50 or 150 μM CK666 (indicated in the x-axis) or vehicle. B-D, The colocalization between F-actin (phalloidin)-Arp2 (B); F-actin and the azurophilic granule marker elastase (C) and Arp2-elastase (D) was analyzed by Airyscan enhance resolution fluorescent confocal microscopy. At least 40 cells from 3 independent mice were analyzed per condition. B-D, The data are represented as mean ± SEM. One-way ANOVA Tukey's multiple comparison test. ***, $p=0.0002$; ****, $p<0.0001$; ns, not significant. E, Representative Airyscan images and colocalization analysis of Arp2-F-actin whose quantification is presented in (B). At least 40 cells from 3 independent mice were analyzed per condition.

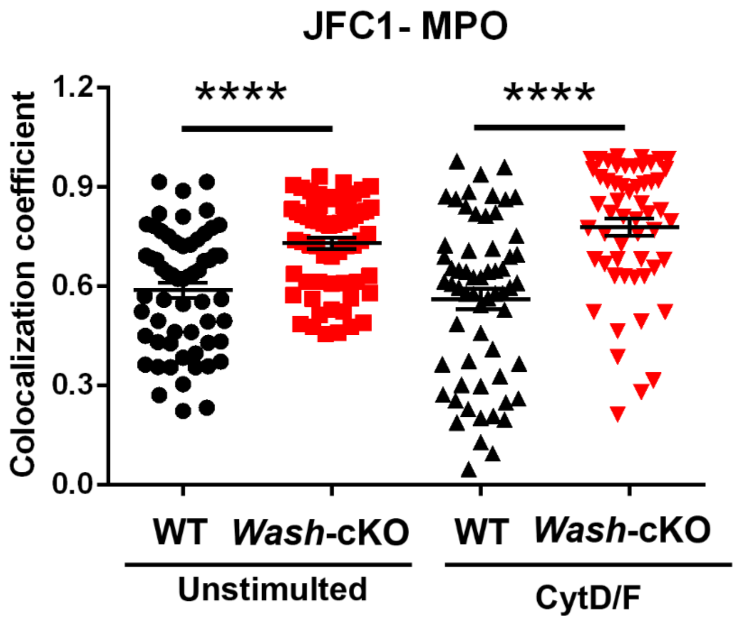

Supplementary Figure 5

Increased recruitment of the Rab27a-effector JFC1 at azurophilic granules in *Wash*-deficiency

Immunofluorescence analysis of the recruitment of endogenous JFC1 at azurophilic granules (MPO) in wild-type and *Wash*-cKO neutrophils that were either left unstimulated or stimulated with cytochalasin D and fMLF (CytD/F). Mean ± SEM. Each symbol represents an independent cell measurement. A total of 59 unstimulated WT cells, 62 unstimulated *Wash*-cKO cells, 60 stimulated WT cells and 54 stimulated *Wash*-cKO cells were analyzed in 3 independent experiments. ****, $p<0.0001$, two-tailed unpaired t-test.

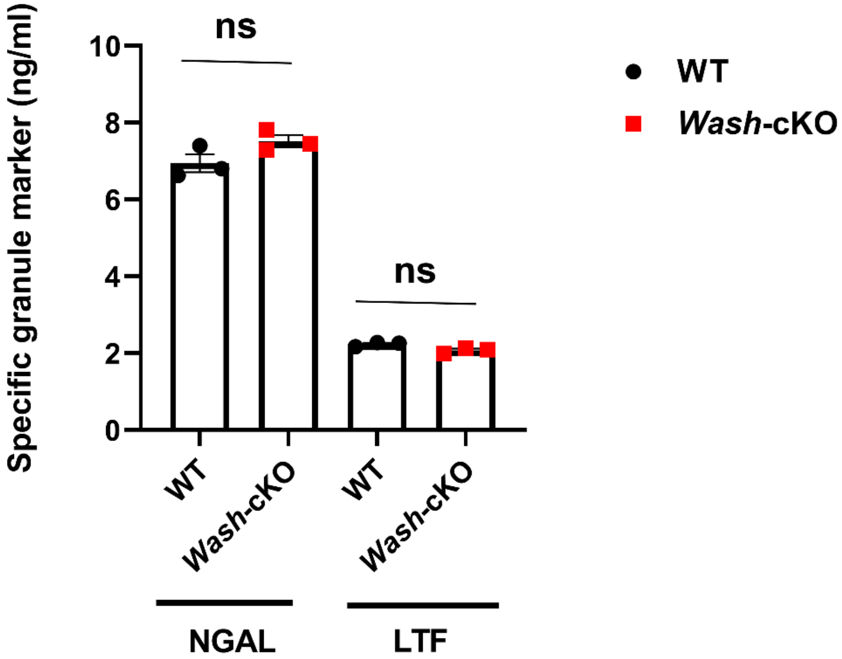

Supplementary Figure 6

Analysis of the basal secretion of Specific granules in *Wash*-cKO neutrophils

The concentration of the Specific granule cargoes NGAL and lactoferrin (LTF) secreted from unstimulated WT and *Wash-cKO Washc1*^{haemo}) neutrophil was determined by ELISA. Mean ± SEM (n=3 independent mice). ns, not significant. Two-tailed Mann-Whitney test.

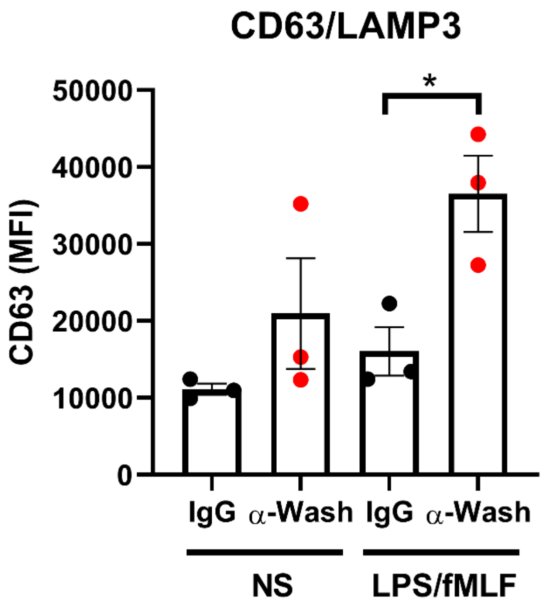

Supplementary Figure 7
Azurophilic granule exocytosis is increased by anti-WASH inhibitory antibodies
Human neutrophils were permeabilized using SLO and incubated in the presence of anti-WASH inhibitory antibodies or IgG control as described under Methods. Exocytosis of azurophilic granules was monitored by the upregulation of the marker CD63 (LAMP3) at the plasma membrane by flow cytometry analysis. n=3 independent donors. Mean ± SEM. *, p=0.025, Two-tailed unpaired Student's t-test.

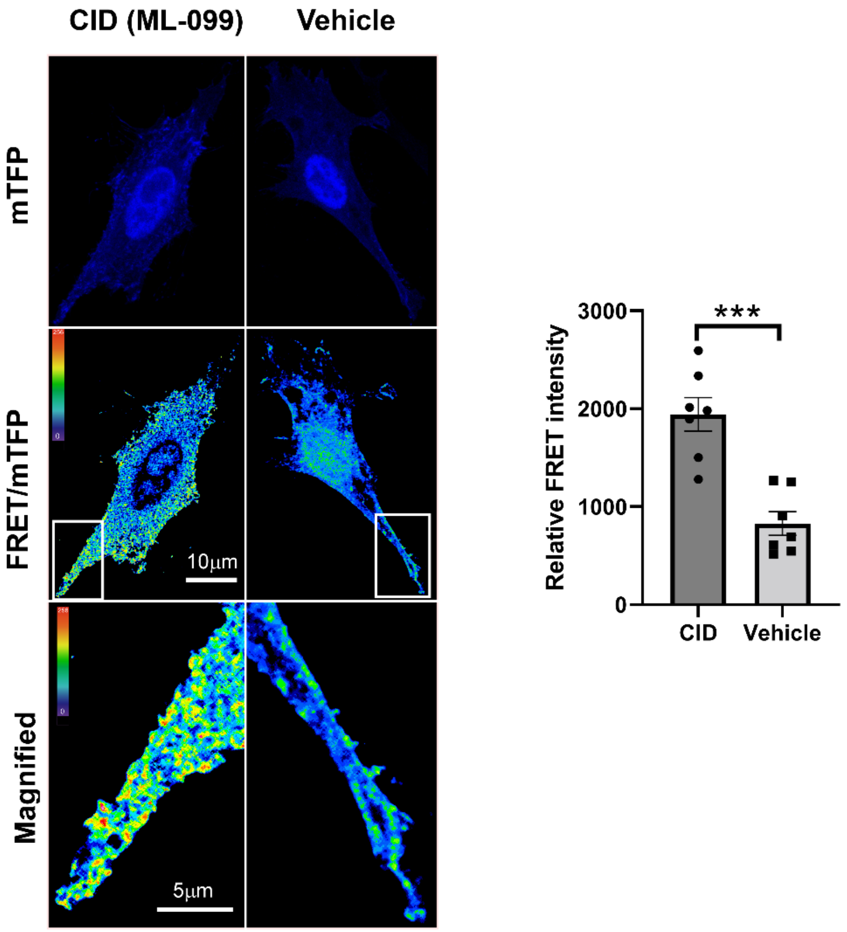

Supplementary Figure 8

Activation of Rac1 by CID888706 (ML-099) using a Rac1 biosensor

Mouse embryonic fibroblasts were transfected with the Rac1-2G biosensor (mTFP-PBD-Venus-Rac1)[1]. Forty-eight hours after transfection, the cells were treated with the Rac1 activator CID888706 (CID)[2] (10 μM) or vehicle for 1 hour, fixed and analyzed using Airyscan enhance resolution fluorescent confocal microscopy. Quantification of FRET signal was performed using the Airyscan integrated FRET module in a Zeiss LSM 880 laser scanning confocal microscope. A, Representative images and magnifications of CID-treated and untreated cells. B, Mean ± SEM of the Relative FRET intensity in CID-treated and untreated cells. Twelve CID-treated and 12 cells untreated cells from seven independent fields were included in the analysis. ***, p=0.0006. Two-tailed Mann-Whitney test.

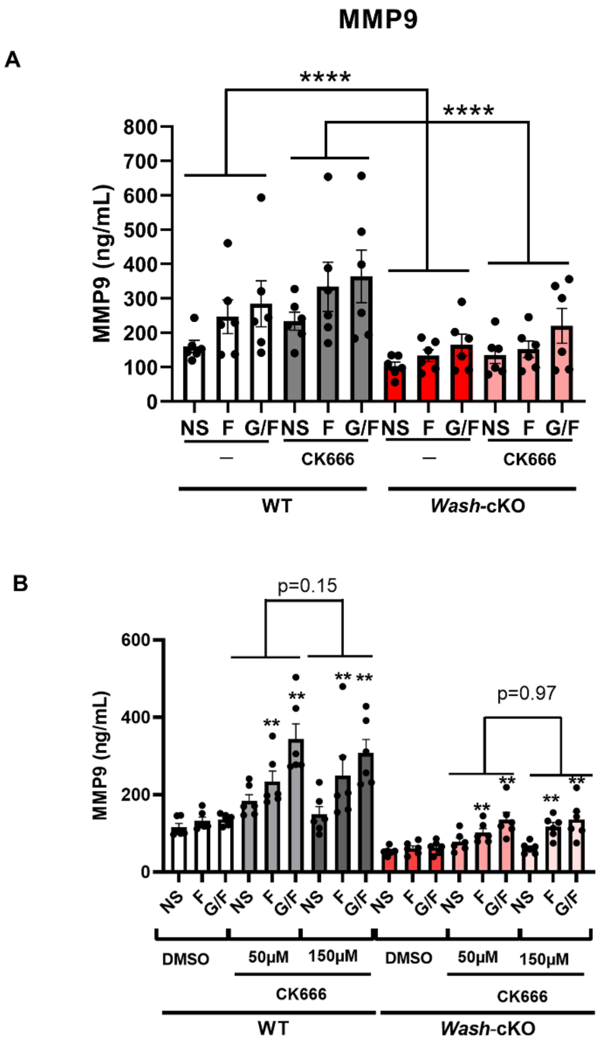

Supplementary Figure 9
The Arp2/3 inhibitor CK666 does not inhibit Gelatinase granule exocytosis
Wild type and *Wash*-cKO (*Washc1^{haemo}*) neutrophils were treated with CK666 (50 µM) or vehicle for 1 hour and subsequently stimulated with fMLF (F) and gelatinase (MMP-9) in the supernatants was analyzed by ELISA. Where indicated, the cells were primed with GM-CSF before stimulation (G/F). A, n=12 independent mice analyzed in two independent experiments. Mean ± SEM. ****, WT vs *Wash*-cKO: $p=2.9\times10^{-11}$, for both CK666 ($p=7.6\times10^{-6}$) and vehicle ($p=7.6\times10^{-6}$) and all each stimulated group within (all p=0.03). CK666 positive *vs* vehicle: $p=2.1\times10^{-6}$. True for both WT ($p=3.3\times10^{-4}$) and *Wash*-cKO ($p=4.0\times10^{-3}$). Two-tailed Wilcoxon signed rank test. B, Comparative analysis of the effects of 50 and 150 µM CK666 on MMP9 secretion. Experiments were performed as in A, except neutrophils were pre-incubated with vehicle or either 50 or 150 µM CK666 before stimulation. No significant differences were observed between treatments with 50 or 150 µM CK-666, two-tailed paired t-test across samples and conditions (NS, F, and G/F), n=6 independent mice. Mean ± SEM. **, p<0.01 between the indicated CK666 condition and its vehicle control in each group (WT or *Wash*-cKO).

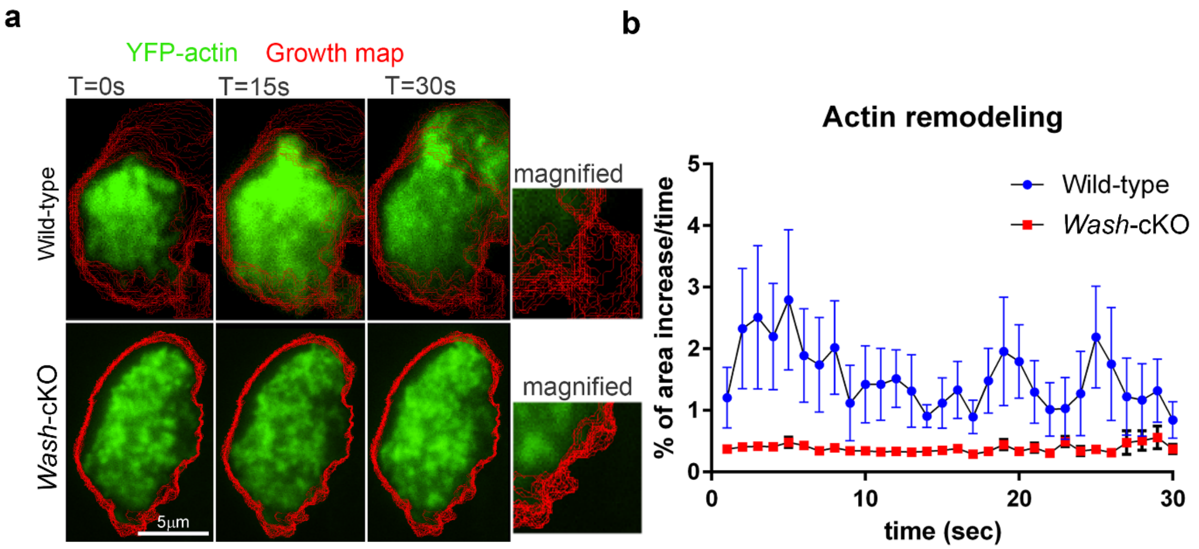

Supplementary Figure 10
***Wash*-cKO neutrophils present altered cell spreading**
a. Analysis of actin (green) remodeling in wild-type and *Wash*-cKO neutrophils expressing YFP-actin, by TIRFM. The red lines represent the growth maps of laminar actin profiles at the indicated time of analysis. The magnified images show that actin remodeling is significantly more active in WT cells. b. Quantification of the cell spreading based on actin labeled growth map as a function of time. The dynamics of YFP-actin remodeling was continuously monitored over the 30 sec in WT (blue symbols) and *Wash*-cKO (red symbols) neutrophils. Each time point on the plot represents the average area increase (%) from n=32 WT and 12 *Wash*-cKO neutrophils analyzed in 2 independent experiments. Mean ± SEM.

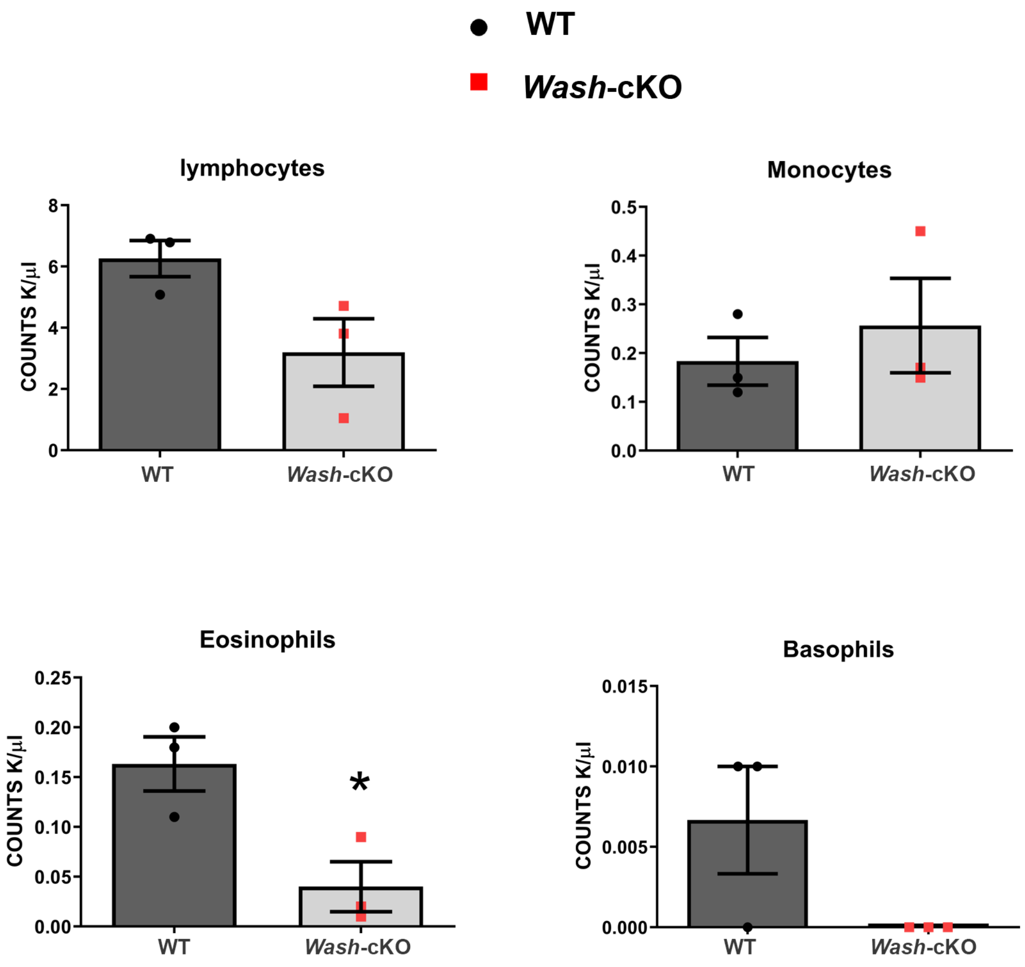

Supplementary Figure 11

Hematology analysis of wild-type and *Wash*-cKO (*Washc1*^{haemo} mice

Quantification of the absolute number of the indicated WBC was performed as detailed under Methods. Mean ± SEM, n=3 independent mice; *, p= 0.0293. Two-tailed, unpaired Student's t-test.

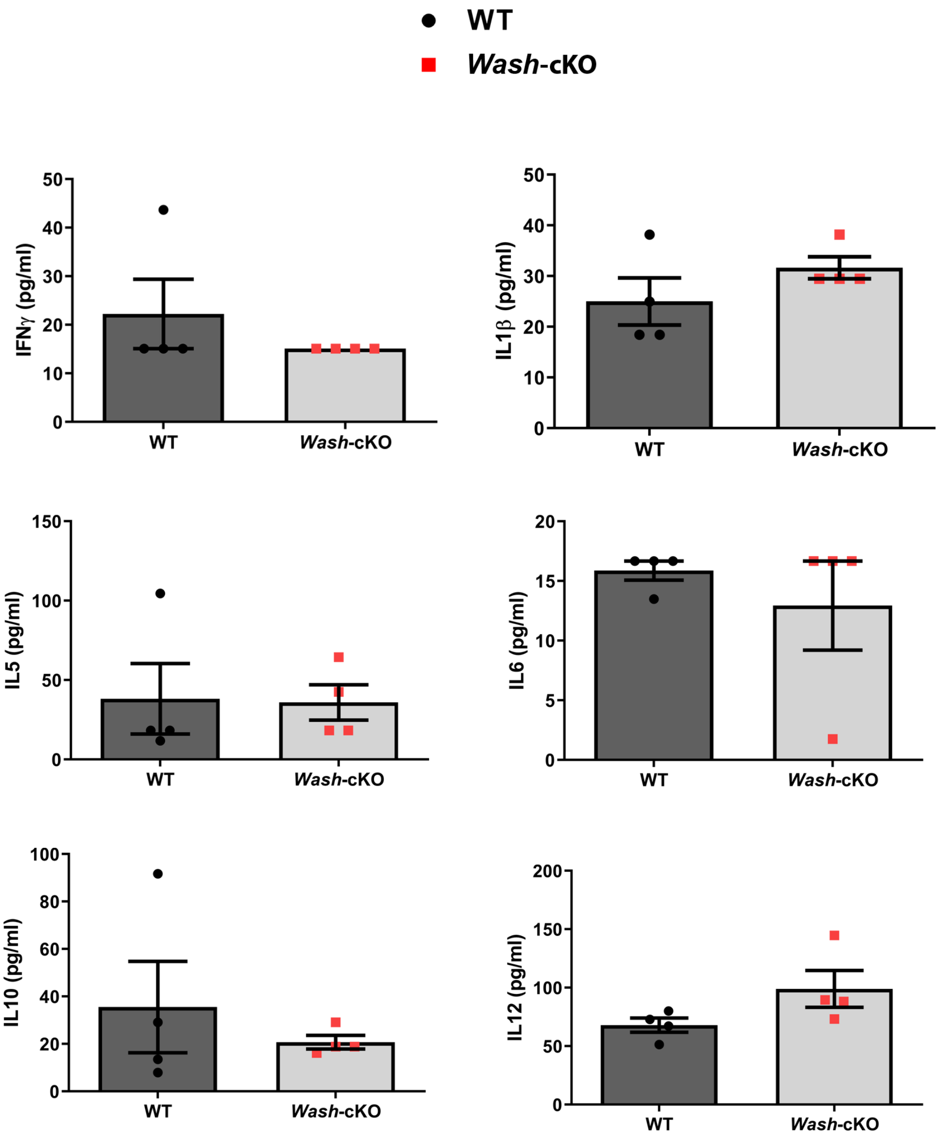

Supplementary Figure 12
Inflammatory profile of unchallenged *Wash*-cKO (*Washc1^{haemo}*) mice
The plasma levels of the indicated cytokines were determined using Multiplex technology (Millipore). Mean ± SEM. n=4 independent mice. All analyses: not significant, two-tailed unpaired Student's t-test.

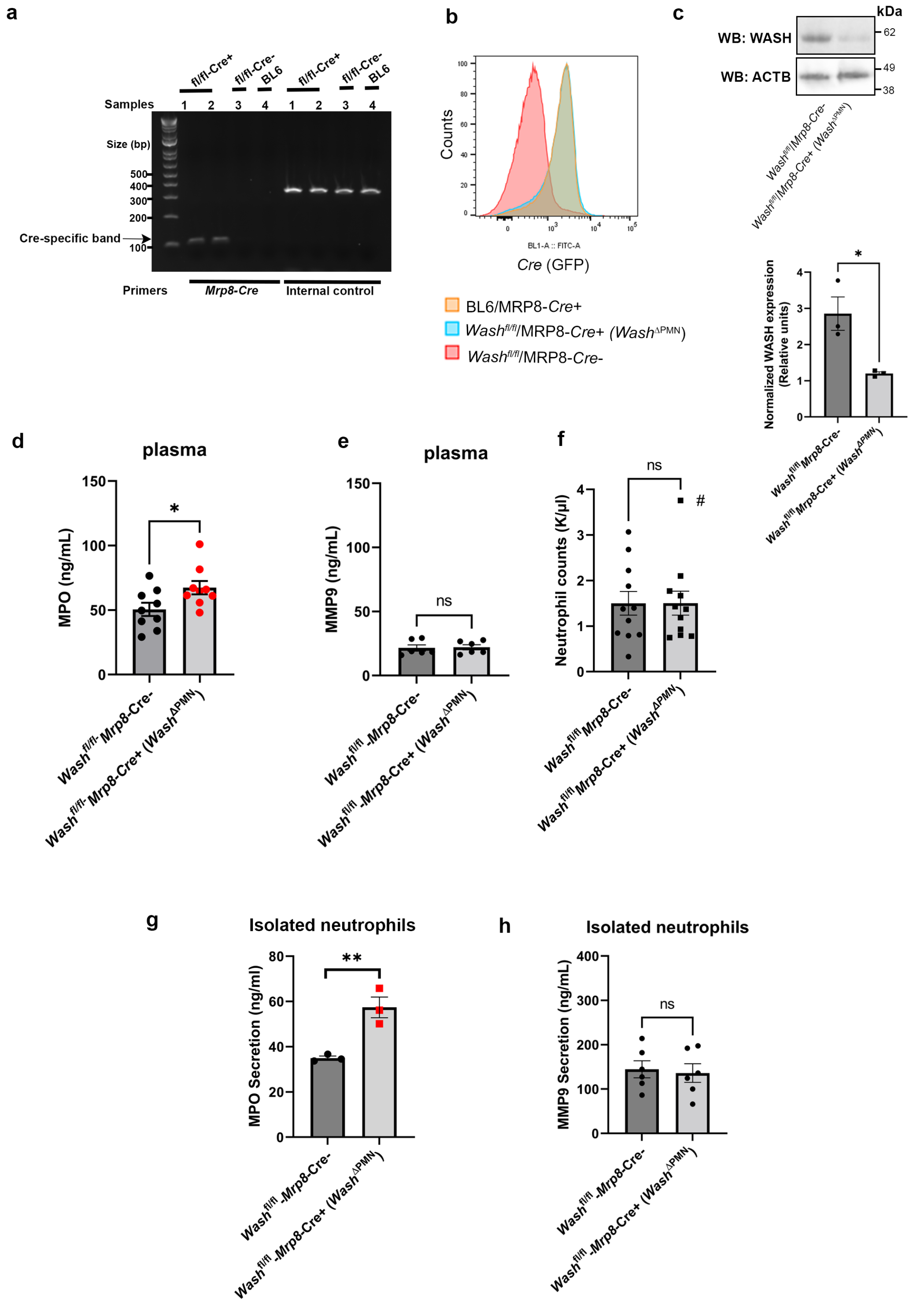

Supplementary Figure 13

Characterization of the *Wash$^{fl/fl}$/Mrp8-Cre*+ mouse model

a, Mice that were genotyped by Transnetyx technology, were further validated for *Cre* integration using the following primers: oIMR1084 (Cre F); oIMR1085 (Cre R); oIMR7338 (internal control F); oIMR7339 (Internal control R) (The Jacksons Laboratory), which produce the expected fragments of ~100 bp (Transgene, *Cre*-specific band) and 324 bp (Internal positive control). fl/fl indicates

Wash$^{fl/fl}$. n=3. **b**, Flow cytometry analysis of GFP (*Mrp8-Cre*-ires/GFP) expression in mature neutrophils isolated from the indicated mouse model by positive selection (Ly6G+). **c**, Western blot analysis of WASH expression in isolated Ly6G+ neutrophils. Upper panel, representative immunoblots of 3 independent mice. Lower panel, quantification of 3 independent experiments. Mean ± SEM, *, p=0.0236, two-tailed unpaired t-test. **d**, Analysis of plasma MPO in *Wash*$^{fl/fl}$-*Mrp8-Cre+* and *Wash*$^{fl/fl}$-*Mrp8-Cre-* neutrophils. n=9. Mean ± SEM. *, p=0.0351 two-tailed unpaired Student's t-test. **e**, Analysis of plasma MMP-9 in *Wash*$^{fl/fl}$-*Mrp8-Cre+* and *Wash*$^{fl/fl}$-*Mrp8-Cre-* neutrophils. n=6. Mean ± SEM. ns, not significant. Two-tailed Student's t-test**.** **f**, Quantitative analysis of neutrophil counts in blood. n=11, 3 independent experiments. Mean ± SEM. ns, not significant with or without outlier (#). Two-tailed unpaired Student's t-test. **g**, Analysis of azurophilic granule exocytosis in isolated unstimulated neutrophils measured as the secreted myeloperoxidase (MPO). n=3 independent mice. Mean ± SEM.**, p=0.0084, two-tailed Student's t-test. **h**, Analysis of gelatinase granule exocytosis in isolated unstimulated neutrophils measured as the secreted gelatinase B (MMP-9). n=6 independent mice. Mean ± SEM. ns, not significant. Two-tailed Student's t-test.

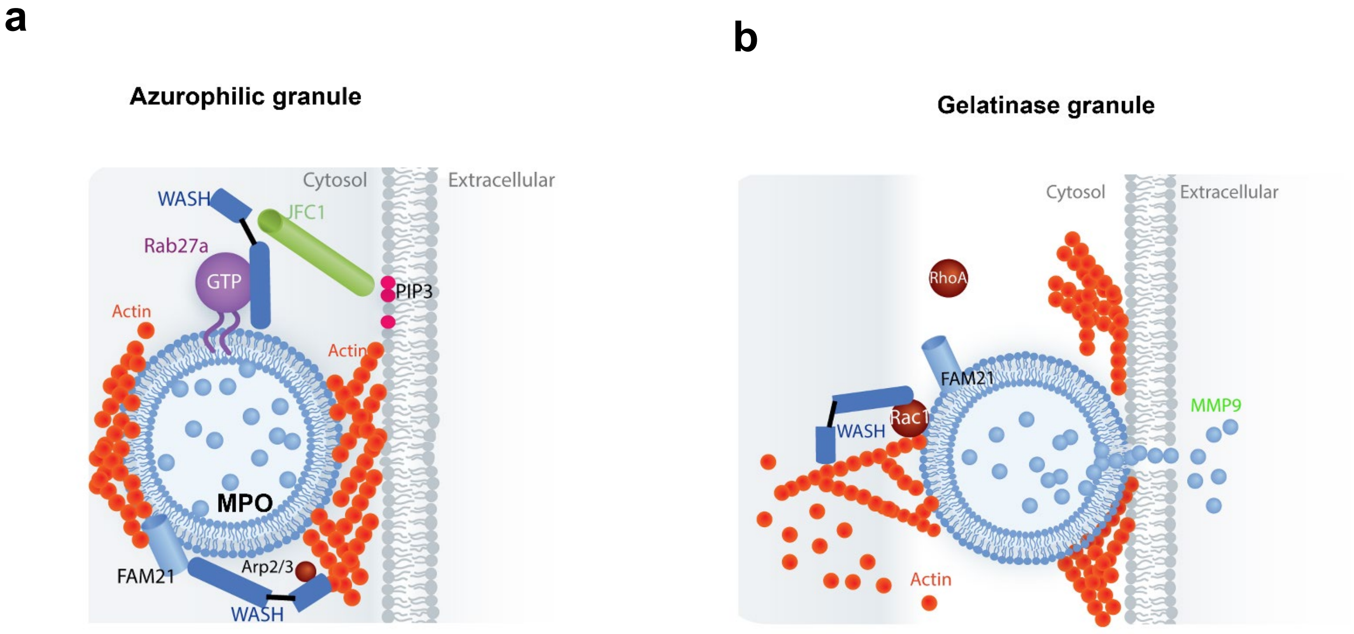

Supplementary Figure 14
Schematic models showing differential control of neutrophil granule subtype exocytosis by WASH
a. Dual role of WASH in the regulation of azurophilic granule exocytosis. WASH interferes with the binding of the small GTPase Rab27a to its effector molecule JFC1 inhibiting docking and preventing unwanted secretion. Actin entrapment of azurophilic granules mediated by WASH contributes to the repression of azurophilic granule exocytosis in an Arp2/3-dependent manner.
b. Role of WASH in the regulation of gelatinase granule exocytosis. WASH contributes to the formation of actin comets that help position gelatinase granules in the exocytic actin zone. Rac1 localization at gelatinase granule requires WASH. In the absence of WASH, FAM21 accumulates at gelatinase granules while Rac1 recruitment is defective leading to impaired actin comet formation, and defective trafficking and exocytosis of gelatinase granules. Adapted with permission from Reference [3].

S14c

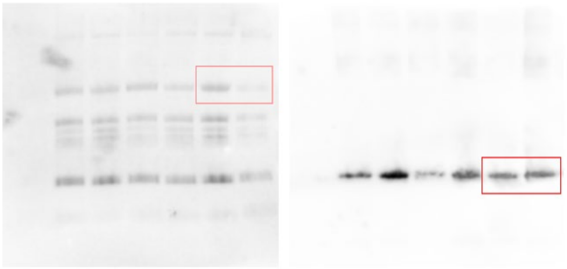

Supplementary Figure 15
Original immunoblots from Supplementary Figure 13c

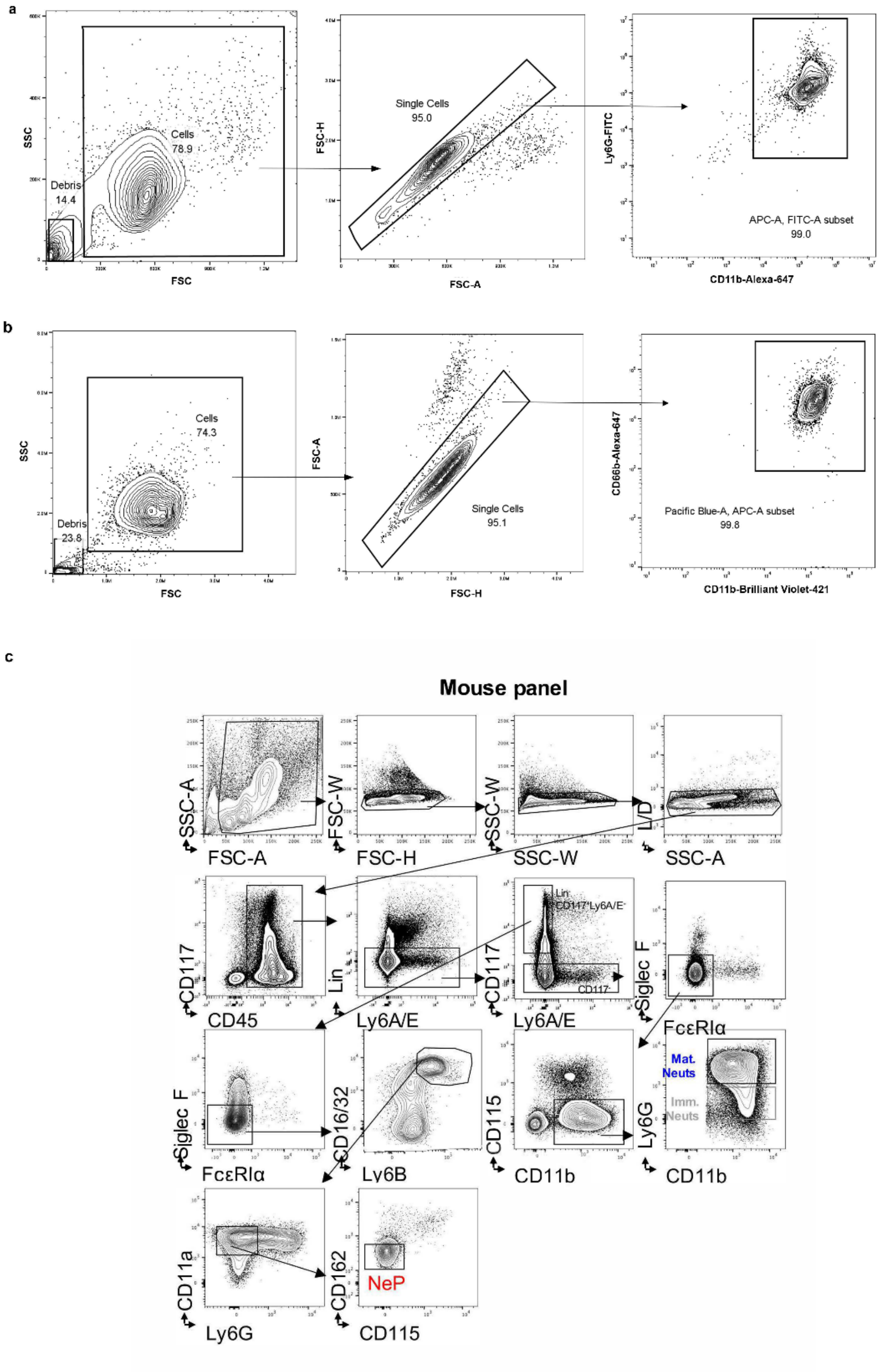

Supplementary Figure 16

Flow cytometry gating strategies for neutrophils and their precursors

a. Gating strategy for mouse mature neutrophils after isolation by positive selection**. b.** Gating strategy for human mature neutrophils after isolation**. c.** Automated single-cell analysis of healthy mouse BM cells using conventional flow cytometry identifies the neutrophil mature, immature and progenitor populations. Figure exemplifying the gating strategy for the identification and sorting of mature and immature neutrophils. (NeP, committed unipotent early-stage neutrophil progenitor).

Supplementary Table 1
Mass spectrometry analysis of the neutrophil secretome (Gelatinase/Specific granules)

Mass spectrometry analysis of gelatinase/secondary granule secretome

lactotransferrin precursor
chitinase-related protein MCRP, partial
alpha-cardiac actin
neutrophil gelatinase-associated lipocalin precursor
myeloid secondary granule protein (bactenecin)
protein S100-A9
14-3-3 zeta
rho GDP-dissociation inhibitor 2 isoform 1
preprocomplement component C3
Epithelin 1 & 2
matrix metalloproteinase-9 precursor
S100-A8 (MRP8 protein)
malate dehydrogenase
coronin-1
CD177 antigen precursor
mCG22220 (G protein-coupled receptor 165)
Phospholipase B-like 1 precursor

Proteins identified in the secretome of both WT and *Wash*-cKO
Proteins identified in the secretome of *Wash*-cKO but not WT
Proteins identified in the secretome of WT but not *Wash*-cKO

Mass spectrometry analysis of the secretome of wild-type and *Wash*-cKO *Washc1*^{haemo}) neutrophils treated with fMLF that favors Gelatinase/Specific granule secretion over azurophilic granule secretion. Grey, proteins detected in both WT and *Wash*-cKO neutrophils secretory supernatant; Red, proteins identified in WT but not in *Wash*-cKO neutrophil supernatants; Blue, proteins identified in *Wash*-cKO neutrophils but not in WT neutrophil's supernatants. 3 independent mice. See associated Supplementary Data 1 and 2.

Supplementary Table 2. Reagents, Vendors and Catalogue numbers

Reagent	Vendor	Catalogue Number	Working dilution (if applicable)
Ly6G MicroBeads UltraPure	Miltenyi Biotec Inc	130-120-337	
Ly6G MicroBead Kit (discontinued)	Miltenyi Biotec Inc	130-092-332	
LS Columns	Miltenyi Biotec Inc	130-042-401	
MACS smart strainers	Miltenyi Biotec Inc	130-110-915	
Alexa Fluor 488 Phalloidin	ThermoFisher Scientific	A12379	
Alexa Fluor 568 Phalloidin	ThermoFisher Scientific	A12380	
Alexa Fluor 594 Phalloidin	ThermoFisher Scientific	A12381	
Alexa Fluor 647 Phalloidin	ThermoFisher Scientific	A22287	
Atto-488 anti-mouse	ThermoFisher Scientific	41051	1:400
Alexa Fluor 488 goat anti-mouse	ThermoFisher Scientific	A32723	1:400
Alexa Fluor 488 goat anti-rabbit	ThermoFisher Scientific	A-11029	1:400
Alexa Fluor 568 donkey anti-rabbit	ThermoFisher Scientific	A10042	1:400
Alexa Fluor 568 donkey anti-goat	ThermoFisher Scientific	A11057	1:400
Alexa Fluor 568 donkey anti-mouse	ThermoFisher Scientific	A10037	1:400
Alexa Fluor 647 donkey anti-goat	Life Technologies	A21447	1:400
Alexa Fluor 647 donkey anti-mouse	Life Technologies	A-31571	1:400
Alexa Fluor 647 donkey anti-rabbit	Life Technologies	A31573	1:400
Alexa Fluor 647 goat anti-rat	Life Technologies	A21247	1:400
Anti-CD63 (LAMP3) (NVG-2) Fluor 647	Biolegend	143921	1:50
Anti-CD11b (clone M1/70) Fluor 647	BD Biosciences	557686	1:50
Anti-Ly6G (clone 1A8)	BD Biosciences	127610	1:50
Anti-neutrophil elastase	Abcam	Ab68672	1:50
Anti-Rac1	Proteintech	24072-1-AP	1:1000
Anti-Rac1-GTP	NewEast Biosciences	26903	1:100
Anti-RhoA	Santa Cruz Biotechnology	SC-418	1:1000
Anti-Rab21	Novus Biologicals	NBP1-81544	1:200
Anti-Lamp1	Santa Cruz Biotechnology	sc-19992	1:200
Anti-Arp2	Abcam	ab49674	1:200
Anti–mouse-Ly6G-FITC (clone 1A8)	Tonbo	35-1276	1:50
Anti-mouse MPO clone 8F4	HycultBiotech	HM1051-100UG	1:200
Anti-MPO	R&D	AF3667	1:400
Anti-mouse MMP-9	R&D	AF909	1:1000
Terbium-conjugated anti-Myc Antibody	Cisbio US Inc	61MYCTAB	1:1000
Anti-mCherry	Abcam	ab213511	1:1000
Mouse MPO Duoset ELISA	R&D Systems	DY3667	
Mouse MMP-9 Duoset ELISA	R&D Systems	DY6718	
Cytochalasin D	Enzo Life Sciences	BMLT1090001	
N-Formyl-Met-Leu-Phe (fMLF)	Sigma-Aldrich	F3506	
LPS from E. coli, Serotype O111:B4 (TLRGRAD)	Enzo Life Sciences	ALX-581-012-L002	
Recombinant Mouse GM-CSF	Shenandoah Biotechnology	200-15-20UG	
Rac1 activator (CID888706, ML-099)	Cayman Chemical	15176	
Arp2/3 inhibitor CK666	Sigma-Aldrich	SML0006	
RhoA inhibitor (C3 Transferase)	Cytoskeleton	CT04	
CXCL1 Protein, mouse recombinant	Sino Biological	50150-MNCE	
CXCL2 Protein, mouse recombinant	Sino Biological	50070-M08Y	
Hypodermic Needle, 30G, 0.5”	Fisher Scientific	1484102	
Plastipak 3-Piece Luer-Lok Syringes	BD Biosciences	300912	
RPMI 1640 Medium, no phenol red	Life Technologies	11835-030	
Fetal Bovine Serum	Corning Cellgro	10,082–147	
Bolt Bis-Tris Plus gels (,)	Life Technologies	NW00122BOX	
Nitrocellulose membranes	GVS Filter Technology	1,215,484	
SuperSignal West Pico	Thermo scientific	34,580	
Revert 700 Total Protein Stain	VWR	103,546–314	
Paraformaldehyde	Electron Microscopy Science	15,710	
Fluoromount-G reagent	SouthernBiotech	0100–01	
Mercaptoethanolamine	Sigma-Aldrich	M3148	
Glucose oxidase	Sigma-Aldrich	G2133-250KU	
4-chamber 35-mm glass-bottom dish	In Vitro Scientific	D35 C4-20-1.5-N	

Supplementary Table 3. All antibodies used in CyTOF and their conjugated labels

HSPC Lineage

Isotope	Metal	Specificity
89	Y	CD45
146	Nd	CD43
151	Eu	CD16/32
156	Gd	CD48
158	Gd	CD34
166	Er	CD117
167	Er	CD150
169	Tm	Sca1

Maturation

Isotope	Metal	Specificity
141	Pr	CD101
145	Nd	Ly6G
155	Gd	Ly6B

Myeloid Lineage

Isotope	Metal	Specificity
142	Nd	CD11c
148	Nd	CD11b
159	Tb	CD169
159	Tb	CD64
159	Tb	CD68
159	Tb	F4/80
160	Gd	CD115
161	Dy	SiglecF
163	Dy	FceRIa

Heterogeneity (activation)

Isotope	Metal	Specificity
149	Sm	62L
152	Sm	CD162

Lymphoid/Erythroid Lineage

Isotope	Metal	Specificity
115	In	CD41
115	In	Ter119
115	In	CD127
115	In	CD3
153	Eu	CD335
147	Sm	CD105
176	Yb	B220

Heterogeneity (function)

Isotope	Metal	Specificity
143	Nd	GPR-32
165	Ho	VEGFR2
173	Yb	TLR4
174	Yb	MHCII

Heterogeneity (migration)

Isotope	Metal	Specificity
144	Nd	CXCR4
154	Sm	CXCR2
164	Dy	CXCR3

Heterogeneity (other)

Isotope	Metal	Specificity
150	Nd	CD24
162	Dy	Ly6C
168	Er	CD79b
170	Er	CD35
171	Yb	LFA-1
172	Yb	CD38
175	Lu	CD16.2

Supplementary Table 4. Antibodies used for CyTOF and dilution factors

Mouse Antigen	Clone	Metal conjugate	Manufacturer	Conjugation	Dilution Factor
CD45	30F11	89Y	Fluidigm	DVS	400
CD43	S11	146Nd	Fluidigm	DVS	600
CD16/32	93	151Eu	Biolegend	In-house	100
CD48	HM48.1	156Gd	Fluidigm	DVS	400
CD34	MEC14.7	158Gd	Biolegend	In-house	100
CD117	2B8	166Er	Fluidigm	DVS	100
CD150	TC1512F12.2	167Er	Fluidigm	DVS	100
Ly-6A/E	D7	169Tm	Fluidigm	DVS	400
CD101	307707	141Pr	Invitrogen	In-house	100
Ly-6G	1A8	145Nd	Biolegend	In-house	100
Ly6B	7/4	155Gd	abcam	In-house	400
CD11c	N418	142Nd	Fluidigm	DVS	400
CD11b	M1/70	148Nd	Fluidigm	DVS	400
CD169	3D6.112	159Tb	Biolegend	In-house	400
CD64	X54-5/7.1	159Tb	Biolegend	In-house	200
F4/80	BM8	159Tb	Fluidigm	DVS	400
CD115	AFS98	160Gd	Biolegend	In-house	100
Siglec F	E50-2440 (RUO)	161Dy	BD Biosciences	In-house	100
FceRIa	Mar-1	163Dy	Biolegend	In-house	400
CD62L	MEL-14	149Sm	Biolegend	In-house	400
CD162	4RA10	152Sm	BD Biosciences	In-house	400
CD41	MWReg30	115In	Biolegend	In-house	800
Ter119	TER-119	115In	Biolegend	In-house	800
CD127	A7R34	115In	Biolegend	In-house	400
CD3	145-2C11	115In	Biolegend	In-house	100
CD335	29A1.4	153Eu	BD Biosciences	In-house	100
CD105	MJ7/18	147Sm	Biolegend	In-house	400
B220	RA36B2	176Yb	Fluidigm	DVS	400
GPR-32	Polyclonal	143Nd	Abcam	In-house	10
CD309 (VEGFR2)	89B3A5	165Ho	Biolegend	In-house	10
TLR4	MTS510	173Yb	Biolegend	In-house	100
MHC class II	M5/114.15.2	174Yb	Fluidigm	DVS	400
CD184(CXCR4)	L276F12	144Nd	Biolegend	In-house	10
CD182(CXCR2)	SA044G4	154Sm	Biolegend	In-house	100
CD183 (CXCR3)	CXCR3-173	164Dy	Biolegend	In-house	400
CD24	M1/69	150Nd	Fluidigm	DVS	200
Ly-6C	HK1.4	162Dy	Biolegend	In-house	400
CD79b	HM79-12	168Er	Biolegend	In-house	100
CD35	11-5/CRTAM	170Er	Biolegend	In-house	200
CD11a (LFA-1)	M17/4	171Yb	Biolegend	In-house	200
CD38	90	172Yb	Biolegend	In-house	200
CD16.2	9E9	175Lu	Biolegend	In-house	200

References

1. Fritz, R.D. *et al.* SrGAP2-Dependent Integration of Membrane Geometry and Slit-Robo-Repulsive Cues Regulates Fibroblast Contact Inhibition of Locomotion. *Dev Cell* **35**, 78-92 (2015).
2. Palsuledesai, C.C. *et al.* Activation of Rho Family GTPases by Small Molecules. *ACS Chem Biol* **13**, 1514-1524 (2018).
3. Johnson, J.L. *et al.* Identification of Neutrophil Exocytosis Inhibitors (Nexinhibs), Small Molecule Inhibitors of Neutrophil Exocytosis and Inflammation: DRUGGABILITY OF THE SMALL GTPase Rab27a. *The Journal of biological chemistry* **291**, 25965-25982 (2016).
