# Supplementary material for: Differential dysregulation of granule subsets in WASH-deficient neutrophil leukocytes resulting in inflammation
Source: Nat Commun. 2022 Sep 21;13:5529. doi: 10.1038/s41467-022-33230-y (PMC9492659; doi:10.1038/s41467-022-33230-y)
Supplement: Supplementary file 2 — Description of Additional Supplementary Files [file 41467_2022_33230_MOESM2_ESM.pdf]

Description of Additional Supplementary Files

File Name: Supplementary Data 1
Description: **Related to Table 1**
Supplementary Data wild type neutrophil secretome proteomics

File Name: Supplementary Data 2
Description: **Related to Table 1**
Supplementary Data Wash-KO neutrophil secretome proteomics

File Name: Supplementary Movie 1
Description: **TIRF microscopy analysis of azurophilic granule docking in WT neutrophils**
WT neutrophils were transfected with EGFP-LAMP3 by nucleofection and analyzed by TIRFM. The video was recorded for 150 seconds, 150 frames. Playback is 10 fps.

File Name: Supplementary Movie 2
Description: **TIRF microscopy analysis of azurophilic granule docking in *Wash*-cKO neutrophils**
Wash-cKO neutrophils were transfected with EGFP-LAMP3 by nucleofection and analyzed by TIRFM. The video was recorded for 150 seconds, 150 frames. Playback is 10 fps.

File Name: Supplementary Movie 3
Description: **TIRF microscopy analysis of actin remodeling in WT neutrophils**
WT neutrophils were transfected with YFP-actin by nucleofection and analyzed by TIRFM. The video was recorded for 60 seconds, 60 frames and 300 ms exposure. Playback is 7 fps.

File Name: Supplementary Movie 4
Description: **TIRF microscopy analysis of actin remodeling in *Wash*-cKO neutrophils**
Wash-cKO neutrophils were transfected with YFP-actin by nucleofection and analyzed by TIRFM. The video was recorded for 60 seconds, 60 frames and 300 ms exposure. Playback is 7 fps.
