# Supplementary material for: Differential dysregulation of granule subsets in WASH-deficient neutrophil leukocytes resulting in inflammation
Source: Nat Commun. 2022 Sep 21;13:5529. doi: 10.1038/s41467-022-33230-y (PMC9492659; doi:10.1038/s41467-022-33230-y)
Supplement: Supplementary file 9 — Reporting Summary [file 41467_2022_33230_MOESM9_ESM.pdf]

nature portfolio

Corresponding author(s): Sergio D. Catz

Last updated by author(s): Jul 13, 2022

Reporting Summary

Nature Portfolio wishes to improve the reproducibility of the work that we publish. This form provides structure for consistency and transparency in reporting. For further information on Nature Portfolio policies, see our Editorial Policies and the Editorial Policy Checklist.

Statistics

For all statistical analyses, confirm that the following items are present in the figure legend, table legend, main text, or Methods section.

n/a	Confirmed	
☐	☒	The exact sample size (*n*) for each experimental group/condition, given as a discrete number and unit of measurement
☐	☒	A statement on whether measurements were taken from distinct samples or whether the same sample was measured repeatedly
☐	☒	The statistical test(s) used AND whether they are one- or two-sided<br>*Only common tests should be described solely by name; describe more complex techniques in the Methods section.*
☐	☒	A description of all covariates tested
☐	☒	A description of any assumptions or corrections, such as tests of normality and adjustment for multiple comparisons
☐	☒	A full description of the statistical parameters including central tendency (e.g. means) or other basic estimates (e.g. regression coefficient) AND variation (e.g. standard deviation) or associated estimates of uncertainty (e.g. confidence intervals)
☐	☒	For null hypothesis testing, the test statistic (e.g. *F*, *t*, *r*) with confidence intervals, effect sizes, degrees of freedom and *P* value noted<br>*Give P values as exact values whenever suitable.*
☒	☐	For Bayesian analysis, information on the choice of priors and Markov chain Monte Carlo settings
☒	☐	For hierarchical and complex designs, identification of the appropriate level for tests and full reporting of outcomes
☒	☐	Estimates of effect sizes (e.g. Cohen's *d*, Pearson's *r*), indicating how they were calculated

Our web collection on statistics for biologists contains articles on many of the points above.

Software and code

Policy information about availability of computer code

Data collection	ZEN-LSM software - Zen 2.3 SP1 FP3 (Black) (64 bit) Release version 14.0.25.201<br>Imaris software - Imaris (x64) version 9.9.0 build 60688; includes Imaris Cell module (version 9.9)<br>NIS-Elements software - NIS-Elements version 5.21.03 (build 1489) (64 bit)<br>Nikon software - same as above<br>Nikon STORM module in Elements software - same as above<br>Nikon off-line analysis - NIS-Elements AR Analysis version 5.20.02 (build 1453) (64 bit)<br>BD FACS Diva 6 software (version 9.0)<br>Cytobank analysis software (https://premium.cytobank.org)<br>Excel software – Microsoft 365<br>Fiji ImageJ Version 1.53s
Data analysis	GraphPad Prism (version 4) software or Excel Microsoft 365

For manuscripts utilizing custom algorithms or software that are central to the research but not yet described in published literature, software must be made available to editors and reviewers. We strongly encourage code deposition in a community repository (e.g. GitHub). See the Nature Portfolio guidelines for submitting code & software for further information.

Data

Policy information about availability of data

All manuscripts must include a data availability statement. This statement should provide the following information, where applicable:

- Accession codes, unique identifiers, or web links for publicly available datasets
- A description of any restrictions on data availability
- For clinical datasets or third party data, please ensure that the statement adheres to our policy

Information on antibodies used is provided in the antibody list in Supplementary Table 1 and in the Methods section. Uncropped gels are provided as Source Data and in the Supplementary Information file. The proteomics data generated in this study have been deposited in PRIME database under accession code XXXX. The immunoblotting data generated in this study are provided in the Supplementary information and Source Data files. The statistical raw data are provided in the Source data file. Source data are provided with this paper. All data that support the findings of this study are available within the article, its Supplementary Information, or from the corresponding author upon reasonable request. NCBI Mus musculus (house mouse) database accessible link: https://www.ncbi.nlm.nih.gov/protein?term=(Mus+musculus+genome)+AND+%22Mus+musculus%22%5Borgn%5D&cmd=DetailsSearch

Field-specific reporting

Please select the one below that is the best fit for your research. If you are not sure, read the appropriate sections before making your selection.

☒ Life sciences ☐ Behavioural & social sciences ☐ Ecological, evolutionary & environmental sciences

For a reference copy of the document with all sections, see nature.com/documents/nr-reporting-summary-flat.pdf

Life sciences study design

All studies must disclose on these points even when the disclosure is negative.

Sample size

Sample size was determined by power analysis. For in vivo studies, by power analyses, we predicted that 15 mice/group will allow for an incidence decrease of 50% in the protected groups to be statistically significant, where the α and desired statistical power levels are 0.05 and 0.88, respectively. Based on our previous studies of neutrophil function using similar mouse models we determined the sample size for colocalization, secretion and ROS assays. For example, as described in references:
The atypical small GTPase GEM/Kir is a negative regulator of the NADPH oxidase and NETs production through macroautophagy.
Johnson JL, Ramadass M, Rahman F, Meneses-Salas E, Zgajnar NR, Carvalho Gontijo R, Zhang J, Kiosses WB, Zhu YP, Hedrick CC, Perego M, Gunton JE, Pestonjamasp K, Napolitano G, Catz SD.
J Leukoc Biol. 2021 Oct;110(4):629-649. doi: 10.1002/JLB.2HI0421-123R.
N-GSDMD trafficking to neutrophil organelles facilitates IL-1β release independently of plasma membrane pores and pyroptosis.
Karmakar M, Minns M, Greenberg EN, Diaz-Aponte J, Pestonjamasp K, Johnson JL, Rathkey JK, Abbott DW, Wang K, Shao F, Catz SD, Dubyak GR, Pearlman E.
Nat Commun. 2020 May 5;11(1):2212. doi: 10.1038/s41467-020-16043-9.
PMID: 32371889
Increased Neutrophil Secretion Induced by NLRP3 Mutation Links the Inflammasome to Azurophilic Granule Exocytosis.
Johnson JL, Ramadass M, Haimovich A, McGeough MD, Zhang J, Hoffman HM, Catz SD.
Front Cell Infect Microbiol. 2017 Dec 11;7:507. doi: 10.3389/fcimb.2017.00507. eCollection 2017.

Data exclusions

All data were included. For statistical outliers we used Grubb's test (alpha=0.05). If present, outliers were included and indicated in the figures. None of the animals were excluded from the analysis.

Replication

Experiments were performed utilizing distinct samples (3 or more independent mice) and experiments were repeated accordingly.
For experiments other than those involving mice, i.e. secretion studies using human neutrophils, TR-FRET studies and pull-down assays, all attempts were successful. Reproducibility was determined using appropriate statistical methods which are described in detail for each of these methods, in the manuscript. A p value <0.05 was considered statistically significant.

Randomization

Most experiments in this manuscript compare cells from wild type and KO mice. All samples were processed and analyzed simultaneously. All sample are split between vehicle or drug/stimuli groups. For in vivo studies , animals in each litter are randomly assigned to the drug or placebo group. For secretion analysis using human neutrophils, the sample was split in two and neutrophils from the same donor were either treated with experimental or control conditions/stimuli, so covarieates are not relevant in these experiments. For other studies not involving mice, for example TR-FRET assays and pull-down assays, the same cell line was split and treated either under control or experimental conditions.

Blinding

For hematological, cytokine and some MPO ELISA studies, the investigators were blinded to group allocation. Because Wash-cKo mice are neutropenic and this is very evident during cell isolation, experiments comparing WT and Wash-cKO isolated neutrophils were not blinded to group allocation. For other experiments, including pull-down assays and TR-FRET the experimentalist was not blinded to group allocation, however, the experiments were repeated independently by different investigators with the same outcome.

Reporting for specific materials, systems and methods

We require information from authors about some types of materials, experimental systems and methods used in many studies. Here, indicate whether each material, system or method listed is relevant to your study. If you are not sure if a list item applies to your research, read the appropriate section before selecting a response.

Materials & experimental systems

n/a	Involved in the study
☐	☒ Antibodies
☐	☒ Eukaryotic cell lines
☒	☐ Palaeontology and archaeology
☐	☒ Animals and other organisms
☐	☒ Human research participants
☒	☐ Clinical data
☒	☐ Dual use research of concern

Methods

n/a	Involved in the study
☒	☐ ChIP-seq
☐	☒ Flow cytometry
☒	☐ MRI-based neuroimaging

Antibodies

Antibodies used

All antibodies are appropriately described in the Method section and in Supplementary table 1. We have now included additional antibody information in Table 2 as well as experimental conditions for these antibodies (i.e. dilutions) in the Method section and here:
Primary antibodies used for Western blotting (WB), immunofluorescence (IF) staining and flow cytometry (FC) were as follows with dilutions inticated in parenthesis: MPO, clone 8F4 (HyCult) (IF: 1:200); MPO, AF3667, R&D (1:400); MMP9, AF909, R&D (WB 1:1000) ; the anti-WASH (WB 1:1000) and anti-FAM21 (IF 1:200) antibodies were described before 25, 28. The anti-CD63 (clone NVG-2), anti-CD11b (clone M1/70) and anti-Ly6G (clone 1A8, 127610) (flow cytometry) antibodies were from Biolegend. (FC 1:50). The anti-neutrophil elastase, Ab68672, was from Abcam (FC 1:50) and phalloidin was from Thermo Fischer. The anti-Rac1 antibody was from Proteintech (24072-1-AP) (WB 1:1000) and the anti-Rac1-GTP from NewEast Biosciences (26903) (IF 1:100); anti-RhoA was from Santa Cruz Biotechnology (SC-418) (WB 1:1000; IF 1:100), anti-Rab21 from Novus Biologicals (NBP1-81544) (IF 1:200) and anti-Arp2 (ab49674) (IF 1:200) was from Abcam. Myc-Tag (9B11) Mouse mAb #2276 (WB:1:1000). anti-myc-(Terbium-conjugated), Cisbio (61MYCTAB) TR-FRET: 1:100. Anti-mCherry antibody Abcam, [EPR20579] (ab213511), WB, 1:1000. All dilutions for the antibodies used in CyTOF are now provided in table 3.

Validation

Our in-house-developed anti-WASH antibody is routinely validated against cells from our Wash-cKO and control models. We routinely verify that new antibody batches are active against the specific target. Antibodies against mouse myeloperoxidase, elastase and MMP-9 are obtained from commercial sources, have been used in my and other labs for years and are routinely validated by WB and IF studies. The anti-MPO antibody 8F4 was betted against the MPO-/- mice (Matthijsen et al. The American Journal of Pathology Volume 171, Issue 6, December 2007, Pages 1743-1752). The anti-MPO antibody MPO, AF3667, R&D has been tested against human and mouse neutrophils as well as against HL-60 promyelocytic cells by the manufacturer. In our hands, it provides specific signal for both MPO chains at 55kDa and 14 kDa in murine neutrophils. The anti-MMP9 antibody has been vetted against the MM9-/- samples , distinguishes between conjugated and unconjugated MMP9 forms (Kim et al, PlosOne https://doi.org/10.1371/journal.pone.0033664). Specificty of the the anti-Rac1-GTP antibody was shown by my group by demonstrating that it pulls-down the Rac1-GTP but not the Rac1-GDP from (Ramadas et al, Journal of Leukocyte biology February 2019 https://doi.org/10.1002/JLB.1VMA0818-320R). The anti-RhoA antibody has been cited in 1,234 publication and extensively validated using several knockdown models. For example, see Nature Cell Biology volume 12, pages477–483 (2010).

Eukaryotic cell lines

Policy information about cell lines

Cell line source(s)

293T cells (ATCC, CRL-3216)

Authentication

STR profiling
CSF1PO: 11,12
D13S317: 12,14
D16S539: 9,13
D5S818: 8,9
D7S820: 11
TH01: 7, 9.3
TPOX: 11
vWA: 16,19
Amelogenin: X

Mycoplasma contamination

Cells are routinely check to ensure they are free of contamination. All test were negative for mycoplasma.

Commonly misidentified lines
(See ICLAC register)

No commonly misidentified cell lines were used in the study.

Animals and other organisms

Policy information about studies involving animals; ARRIVE guidelines recommended for reporting animal research

Laboratory animals

C57BL/6 Washc1flox/flox/Vav-cre+ mice (Wash-cKO) and C57BL/6+/flox/Vav-cre- (WT) or C57BL/6 controls were used in this work.

Laboratory animals	The Washc1flox/flox /Vav-cre+ mouse conditional knockout model, lacking WASH expression in the haemopoietic linage Washc1haemo, hereon Wash-cKO) was generated as previously described58. Washfl/fl/Mrp8-Cre+ mice (hereon Wash-deltaPMN) were generated by crossing the Washfl/fl mice with the Mrp8-Cre+ (Tg-S100A8-cre,ires-EGFP)1Ilw mice which expresses Cre recombinase under the promoter of the neutrophil specific cargo S100A8 (Mrp8), and directs bicistronic Cre and EGFP protein expression to CD11b+, Ly6G+, granulocytes (The Jackson Laboratory). The characterization of this model was performed by genotyping using Transnetyx technology for the detection of both the the presence of the deleted allele and the Cre allele, PCR for confirmation, flow cytometry and immunoblotting analysis of WASH expression in Washfl/fl/Mrp8-Cre+ mice (Supplemental Fig. 14a-c). Experimental males and females, 6 to 10-week old mice and sex and age-matched control mice were used in this study.
Wild animals	No wild animals were used in the study.
Field-collected samples	No field collected samples were used in the study.
Ethics oversight	All animal studies were performed in compliance with the Department of Health and Human Services Guide for the Care and Use of Laboratory Animals. All studies were conducted according to National Institutes of Health and institutional guidelines and with approval of the animal review boards at The Scripps Research Institute.

Note that full information on the approval of the study protocol must also be provided in the manuscript.

Human research participants

Policy information about studies involving human research participants

Population characteristics	The experiments interrogate mechanisms using human neutrophils isolated from peripheral blood. This was the only involvement of human subjects in the proposed research. Donors for phlebotomy were selected by the staff of the Normal Blood Donor Services (NBDS) at The Scripps Research Institute. Representative racial and gender composition was drawn from the normal donor pool of the NBDS. The Principal Investigator has no contact with the blood donor and receives number-coded vials. Samples from adult volunteers (18 to 55-year old) of both sexes and any race were requested and used for these studies. The NBDS blood pool is derived from the employees of The Scripps Research Institute and includes the following individuals: 70% female, 30% male, 8% Hispanic, 5% Asians, 3% Afro-American, 0% Native Americans. This project seeks to answer basic cellular and molecular questions concerning neutrophil exocytosis. Therefore the issue of women, children and minority subjects is not germane to this basic research project. Consent was sought by NBDS staff or (for pheresis) staff at the San Diego Blood Bank.
Recruitment	Recruitment was performed by by the staff of the Normal Blood Donor Services (NBDS) at The Scripps Research Institute. Representative racial and gender composition was drawn from the normal donor pool of the NBDS. The Principal Investigator has no contact with the blood donor and receives number-coded vials. Volunteers of both sexes and any race were requested for these studies.<br><br>The Normal Blood Donor Services welcomes all healthy individuals in the community who are readily available during blood drawing hours of 6:00am - 11:00am.<br>Individuals volunteers either find the on-line call by themselves or respond to notices and fliers which are regularly sent out to recruit donors from the community.<br>The recruitment of donors is not limited to Scripps Research employees, but to others in the community.<br>All eligible donors are initially screened for infectious diseases prior to being enrolled in the program, and on a yearly basis after the initial screen to remain active in the program. The pool of donors is comprehensive and therefore there are no biases identified in this pool.<br><br>The Annual Donor Screen consists of the following:
Ethics oversight	Human neutrophils were isolated from normal donor's blood by Ficoll density centrifugation as previously described (Ref. 59). All procedures regarding human subjects have been reviewed and approved by the Human Subjects Committee at The Scripps Research Institute and were conducted in accordance with the requirements set forth by the mentioned Human Subjects Committee and in accordance to NIH guidelines. Informed consent was provided by the donors.

Note that full information on the approval of the study protocol must also be provided in the manuscript.

Flow Cytometry

Plots

Confirm that:

☒ The axis labels state the marker and fluorochrome used (e.g. CD4-FITC).

☒ The axis scales are clearly visible. Include numbers along axes only for bottom left plot of group (a 'group' is an analysis of identical markers).

☒ All plots are contour plots with outliers or pseudocolor plots.

☒ A numerical value for number of cells or percentage (with statistics) is provided.

Methodology

Sample preparation	Human and murine neutrophils were isolated by purification methods that provide >98.5% purity.

Instrument	NovoCyte 3000 with NovoSampler Pro, ACEA Biosciences, Inc.
Software	BD FACS Diva 6
Cell population abundance	>98.5 %. Neutrophil preparations are routinely analyzed for purity using appropriate markers (CD11b/CD66b for human and CD11b/Ly6G for mouse).
Gating strategy	For mature neutrophil analyses, only freshly isolated highly-purified cells (>98.5%) are used and therefore only singlet gate is used. For immature neutrophil populations, the gating strategy for flow cytometry analysis of neutrophil precursors has been previously published by our group (Ref. 66 and 67) . In addition, a figure exemplifying the gating strategy can be found in the Supplementary Information (Supplementary Fig. 17).

☒ Tick this box to confirm that a figure exemplifying the gating strategy is provided in the Supplementary Information.
